# Supplementary material for: COVID-19 impacts equine welfare: Policy implications for laminitis and obesity
Source: PLoS One. 2021 May 28;16(5):e0252340. doi: 10.1371/journal.pone.0252340 (PMC8162578; doi:10.1371/journal.pone.0252340)
Supplement: S1 Table — The inclusion and exclusion criteria used for sampling appropriate sub-populations to gain insight into laminitis and obesity concerns during the COVID-19 pandemic. (DOCX) [file pone.0252340.s001.docx]

**S1 Table. Inclusion and exclusion criteria**

| **Group** | **Inclusion criteria** | **Exclusion criteria** |
| --- | --- | --- |
| **Horse owner with horse at home** | - Horse owner within Aberdeenshire region. - Owner of ≥ 1 native type horse/pony kept on their home residence. | - Horse owners with non-native breed type horses / ponies. - Horse owners with all horses / ponies at livery. |
| **Horse owner with horse at livery** | - Horse owner within Aberdeenshire region. - Owner of ≥ 1 native type horse/pony kept at an establishment or area outside of their home residence, for which they pay an additional fee for housing their horse. | - Horse owners with non-native breed type horses / ponies. - Horse owners with all horses / ponies at home. |
| **Farriers** | - Registered farriers in the Aberdeenshire region | - Unregistered farrier |
| **Equine veterinarian** | - Registered veterinarian practicing in the Aberdeenshire region. - Specialism in equine or mixed large animal practice. | - Specialist small or farm animal veterinarian. |
| **Welfare Centre Manager** | - Manager of an equine welfare centre registered charity in the UK. | - Manager of welfare centre that is not a registered charity - Manager of welfare centres excluding equids. |

The inclusion and exclusion criteria used for sampling appropriate sub-populations to gain insight into laminitis and obesity concerns during the COVID-19 pandemic.
