# Supplementary material for: COVID-19 impacts equine welfare: Policy implications for laminitis and obesity
Source: PLoS One. 2021 May 28;16(5):e0252340. doi: 10.1371/journal.pone.0252340 (PMC8162578; doi:10.1371/journal.pone.0252340)
Supplement: S2 Table — Units of study. The characteristics of each participant are presented in S1 Table. This concise and purposefully selected sample was agreed upon as providing a wealth of experience on the subject of managing laminitis, and as being directly impacted by the pandemic. (DOCX) [file pone.0252340.s002.docx]

**S2 Table: Classification of interviewees, additional details and length of interviews**

| **PARTICIPANT** | **ADDITIONAL INFORMATION** | **LABEL** | **INTERVIEW LENGTH (MINUTES)** |
| --- | --- | --- | --- |
| **Farrier category (F) n=4** | | | |
| **Local farrier** | >20 years’ experience | **F1** | 36.14 |
| **Local farrier** | >20 years’ experience | **F2** | 27.07 |
| **Local farrier** | >20 years’ experience | **F3** | 30.09 |
| **Local farrier** | <10 years’ experience | **F4** | 40.12 |
| **Horse owner with horses at home category (HH) n=6** | | | |
| **Horse owner at home** | Three ponies. Retiree. Regularly competes in showing classes. | **HH1** | 42.15 |
| **Horse owner at home** | Two ponies. Leisure riding. Shetland with laminitis | **HH2** | 30.51 |
| **Horse owner at home** | One Highland pony competes in showing classes and dressage. Horse previously had laminitis and (now resolved) obesity. | **HH3** | 41.52 |
| **Horse owner at home** | Retiree. Welsh riding pony with EMS + companion pony. Previously evented +dressage on Welsh pony. | **HH4** | 35.08 |
| **Horse owner at home** | Professional freelance groom. Three horses. Self-employed – out of work since lockdown. Competes in eventing. Clydesdale young horse. | **HH5** | 37.12 |
| **Horse owner at home** | One Connemara and children’s ponies. Connemara had laminitis. Health care worker. | **HH6** |  |
| **Horse owner with horses at livery category (HL) n=5** | | | |
| **Horse owner at livery yard** | One horse, cares for other horses as volunteer on the yard. Limited yard / pasture maintenance. Outdoor arena. | **HL1** | 34.19 |
| **Horse owner at livery yard** | Owned livery yard themselves. Facilities include indoor / outdoor arena. Three of their own horses cared for by yard staff. Closed yard to owners. | **HL2** | 33.20 |
| **Horse owner at livery yard** | Rented field with shelter. Shetland pony with laminitis and Arab cross riding horse. Takes Shetland out in hand. | **HL3** | 37.09 |
| **Horse owner at livery yard** | One riding pony. Large livery yard / riding school. Extensive facilities on site. | **HL4** | 35.29 |
| **Horse owner at livery yard** | Livery yard / riding school. Works part time at livery yard- furloughed. Two horses, one with chronic EMS. | **HL5** | 30.13 |
| **Veterinarian in equine practice category (V) n=5** | | | |
| **Equine veterinarian** | Large practice. Equine specialism. | **V1** | 43.14 |
| **Mixed large animal veterinarian** | Freelance mixed large animal with equine interest. | **V2** | 33.2 |
| **Mixed practice veterinarian** | Small animal, farm and equine with equine interest. | **V3** | 24.39 |
| **Equine veterinarian** | Large practice. Equine specialism. | **V4** | 34.44 |
| **Mixed large animal veterinarian** | Large practice. Equine specialism. | **V5** | 21.03 |
| **Welfare centre manager category (WCM) n=4** | | | |
| **Welfare centre manager (Aberdeenshire)** | International charity. | **WCM1** | 45.00 |
| **Welfare centre manager (Aberdeenshire)** | Local charity. | **WCM2** | 26.38 |
| **Welfare centre manager (Aberdeenshire)** | International charity | **WCM3** | 18.48 |
| **Welfare centre manager (Aberdeenshire)** | International charity | **WCM4** | 17.32 |

Units of study. The characteristics of each participant are presented in Table S1. This concise and purposefully selected sample was agreed upon as providing a wealth of experience on the subject of managing laminitis, and as being directly impacted by the pandemic.
