# Supplementary material for: COVID-19 impacts equine welfare: Policy implications for laminitis and obesity
Source: PLoS One. 2021 May 28;16(5):e0252340. doi: 10.1371/journal.pone.0252340 (PMC8162578; doi:10.1371/journal.pone.0252340)
Supplement: S3 Table — Examples of direct interview text and the categorisation of data. Meaning units were condensed, and theoretical codes assigned, and the underlying meaning of the quote was interpreted utilising annotations taken during interviews. Sub-themes and themes then emerged as the frequency and importance of concepts arose. (DOCX) [file pone.0252340.s003.docx]

**S3 Table. An example of the coding process used to determine appropriate themes**

| **Meaning unit** | **Theoretical code** | **Contextualisation**  **(utilising annotations)** | **Subtheme** | **Theme** |
| --- | --- | --- | --- | --- |
| “…I think because people are busier managing their own horses, and farriers and vets, those that should have been checked more regularly are not being checked. Also, to be fair, people want to go out and ride, the laminitics are not a priority as much as they have been in previous years.”- HL*2* | Laminitis lower priority due to increased biosecurity measures increasing yard workload | Increased yard workload reduced frequency of field checks for laminitis | Turning horses away | Implications of public health measures on preventative care for obesity and laminitis |
| “their solution to not having liveries up was just to chuck all the horses out in the field.” *–* V4 | Biosecurity requirements led to horses being turned away | Increased workloads on yards due to owners not caring for their animals led to horses being turned away | Turning horses away |  |
| “Actually, quickly after I stopped riding, my highland developed a very severe bout of laminitis…I think it was a combination of things, yes, the reduction of exercise, as well as a bit of taking my eye off the ball.” HH3 | Stopped riding, adjusted priorities, and horse developed laminitis | More important to protect the NHS than to continue riding during pandemic. | Stopped riding | Outcomes of minimising the risk of physical injury |

Examples of direct interview text and the categorisation of data. Meaning units were condensed, and theoretical codes assigned, and the underlying meaning of the quote was interpreted utilising annotations taken during interviews. Sub-themes and themes then emerged as the frequency and importance of concepts arose.
