# Supplementary figures and images for: COVID-19 impacts equine welfare: Policy implications for laminitis and obesity
Source: PLoS One. 2021 May 28;16(5):e0252340. doi: 10.1371/journal.pone.0252340 (PMC8162578; doi:10.1371/journal.pone.0252340)

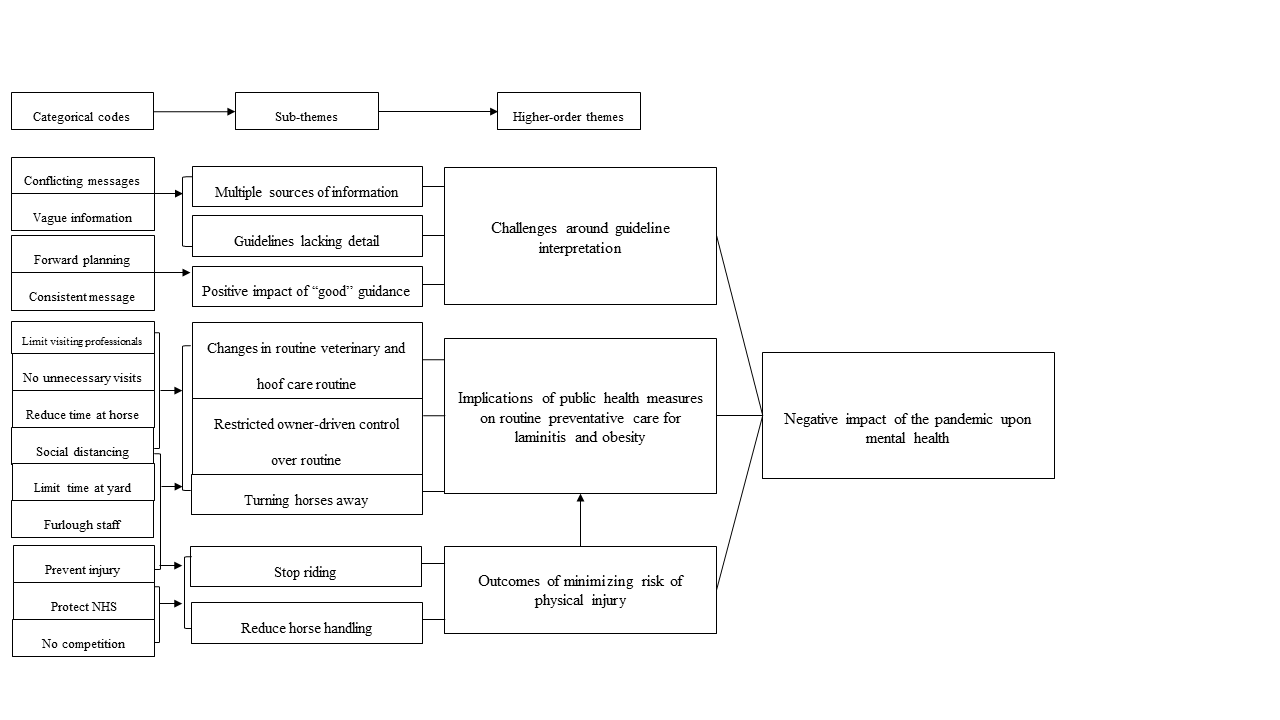

Supplement: S1 Fig — (TIF) [file pone.0252340.s004.tif]
