# Supplementary material for: COVID-19 impacts equine welfare: Policy implications for laminitis and obesity
Source: PLoS One. 2021 May 28;16(5):e0252340. doi: 10.1371/journal.pone.0252340 (PMC8162578; doi:10.1371/journal.pone.0252340)
Supplement: S1 File — (PDF) [file pone.0252340.s005.pdf]

Interview 1: F1

AW: Can I ask you to describe the main challenges you have faced during the pandemic in regards to carrying out your work?

F1: Being perfectly honest we haven't had many challenges. We were deemed critical workers from the start, so in terms of access and moving around the country, we had no real limitations. It has been fairly normal. Challenges would be managing clients who had the fear- and what we did with that was that we had PPE with us, we explained what we were doing to protect them, and we adjusted. For some it was adjusting scheduling which could be challenging, Yards with a rota system could be difficult to organise- where people maybe couldn't bring the horse in for a certain time because it was out-with their time. For home kept horses, it was challenging if they had a vulnerable person in their family. For example, a client whose daughter has cystic fibrosis. But through communication we were able to come up with a plan that protected them as best we could. And the risks, at least I felt, were limited. We weren't overly restricted; time was the biggest restriction. Actually, some of it was quite nice, because we saw less clients. The yards were quieter, which was nice to be honest. We could be more efficient, and just not have to talk to people. We were able to focus on that. But obviously, the conversation and communication are a big part of farriery, it is managing client's expectations and needs. That would be another challenge. The clients were all super worried when we went into lockdown because they didn't know if their horses would continue to be seen. I managed that through social media. Especially the clients with horse with issues. Having spent a long time and a lot of money getting their horses right, to suddenly think that we weren't' going to see their horses- so they were worried. Practically, the impact hasn't been that much. In terms of business, I thought we were quieter, but I don't think we were. We appear to have maintained the same amount of work- specifically to my business, because I have an apprentice, we split into two vehicles. So, we increased efficiency- if there was a lost shoe, one of us could go and do it. So, we felt quieter and we were getting things done quicker through splitting up and spreading out. Even that said, it felt a lot quieter than the cashflow would indicate. So that is one stress that I didn't have. Our stress levels have even relatively low- in business and psychologically- I was glad I was able to go and shoe horses.

AW: You mentioned that people were worried about missing shoeing's- would you say you have missed any shoeing slots?

F1: In every case we have managed to shoe our horses. As long as your flexible and you communicate, these things are always solvable. It wasn't difficult. We had one client who took a lot of shoes off, the big riding school we do, because they are such a good client- they said they weren't going to shoe anything- so we have just shod them and not charged them for this cycle. But we will recoup that, again, it is about us all working together to minimise the impact. I could afford to take that hit. So, you know, these things do come back, in karma, hopefully, and it will be recognised in the future.

AW: That is interesting, as it is the first instance I have noticed of two (areas) in the industry cooperating to get through things. So obviously your job is intense- in terms of taking care of yourself, have you been impacted?

F1: Actually, an advantage was that I have been more aware of not going into shops. And so, for the first month I was definitely better at planning. My normal maybe would have been to stop somewhere for lunch, but the impact was actually positive as I was being more organised. And it is better for you, you save money and it is better nutritionally. But in actual fact, that wasn't a big issue. It was a relatively simple adjustment to make. What we did notice, was that clients who did tend to feed and water us- we weren't accepting it. We altered our behaviour, but it wasn't a hardship.

AW: In terms of the horses you have been seeing, have you had any concerns for welfare?

F1: Mostly everything is as usual. We had a couple of yards who had stopped owners going up to them, and there had been a psychological impact on the horses- with many becoming a little feral. In one case, there was a horse which had been out in a field for four weeks, and it was an absolute nightmare to shoe. There have been cases where people have maybe altered their contact with the horses and so the horse's psychological welfare was maybe impacted. The world just carried on as normal in the horsey bubble for us. People are largely still riding, some are doing more varied exercise- with more hacking, people were maybe working from home and were more flexible with their time. Our competition horses, we are definitely not working at the rate we would normally be, and we are not putting stud holes in and things like that. But in terms of welfare, I am aware anecdotally of welfare issues where yards imposed full livery and then didn't do a very good job of it, but most of the welfare would have been for the horse owners where they were restricted from seeing their horses. I felt quite strongly that people who have horses really needed their horses right now, and they were restricted when they needed them most. But that was down to individual yards and I think it is different if your yard is on your home. And if you are fearful, then that could be challenging. Personally, I haven't seen any worse welfare cases than we come across anyway.

AW: That is great. And with laminitis specifically, have you seen the usual amount of cases that you would be seeing?

F1: Probably, Definitely less than last year. Last year was a crop year, it was a bit of a perfect storm. Laminitis has definitely been round and about, it's probably – you know we don't write down each case- but also, I am quite pro-active on the laminitis, I'm not frightened to tell you that your horse is fat. So, I feel that we have been instrumental in averting some laminitic episodes by being pro-active and talking about weight and management -muzzles, strip grazing. For me muzzles are the gold standard. But getting people to get their horses off the grass and do basics like weighing their hay nets. That's fine in the winter not to, but at this time in the year, we all overestimate what we think our horses or ourselves need in terms of nutrition. So, we have always worked quite hard to be part of the advice behind management =- not just footcare, but field management and stuff like that.

AW: Do you find people quite receptive to you when you are offering this advice?

F1: Yeah, I think some of them do and you get good at working out the ones where you aren't going to make a difference. You try it twice, and nothing changes, so you say I can't save this horse. They are always the ones to lament the most when their horse actually crashes. I think one of the challenges we have is that we have a lot of first-time horse owners now, who have never seen laminitis. So, until you see it, you just have to see their eyes to wish that you could eradicate it. So I will always start the conversation, and I am pretty confident that I know which ones are worth having it with- and others where you thin- I'll say what I need to say but I know nothing will change. If you push it with those ones, they almost become resentful and they will almost go out of their way to not help the horse. We have had ones where we have been worried that they maybe have PPID or metabolic issues, and they are almost jubilant when they find out that it doesn't have Cushing's… So then why does it have sore feet. That is more of a worry if than if it did have Cushing's, because then you don't know why you have these fat patches. Instead of seeing that there is still a problem, they will always have excuses. I can't use a muzzle because, or I can't strip graze because. I can't do this, or I can't do that.

AW: Usually if you have a laminitis case, are you managing this with the vet?

F1: Well once they are in a laminitic state, I hand over to the vets. I believe that the start of laminitis is a vet job- we can help in the initial first aid and comfort- we are talking a vet emergency. If they are just a bit off, and at the risk of overstepping our remit as farriers, I think I would tend to just give them my advice. That would be just keep them in the box and get them off the grass and immobilise them for a few days. Especially with the ones that have already had a laminitic episode and you are just managing flare ups. I would class those as critical. I would look at the horse and, beyond that subclinical case- there are no questions asked just to call the vet. Then I don't expect to be involved in that for another three weeks. I think they need to be allowed to settle before we try to do anything in terms of foot care.

AW: Would you say that the social distancing has changed anything in the way you would manage a case? For example, if you were going to see a horse more frequently, or if you needed collaboration from more than one person, has social distancing changed how you would approach that?

F1: The majority of people were nice enough that they wouldn't be too arsey about it. If we have to get close, then we can't eliminate that. It doesn't mean that it is terrible, it is just that we have increased the risk. The BFBA at the start of the lockdown put out a traffic light system for us. So, red was- it has to be shod, amber was they could maybe go a little while, and green was it doesn't need anything done. Now most horses were amber, as they are on a schedule for a reason. If they are on a 6-week schedule, there will be a problem by 8 weeks. The ones in the red were youngsters, because you had to have people holding them so that was contradictory. If we are sensible, and you know, we are not an urban population. I think there was still a bigger risk in going to Tesco. It would be other people's worries that would dictate the precautions we put in place.

AW: So, it sounds as though you had a nicely structured piece of information from the BFBA, would you say that what they put out was sufficient to keep you confident that you were adhering to appropriate standards?

F1: Yeah, it didn't change anything I would have done anyway, because I feel strongly that we mustn't compromise horse welfare over acceptable risk. So, at no point would it have been acceptable for us not to carry out hoof care, is my opinion, because the risk of contamination or of spreading disease is low. We are not breathing on people; we would stay off work if we have symptoms. As long as we were following those things. The animals were unable to do anything about this, and so they shouldn't suffer. There were some animals that didn't need done- the trims especially, if we are doing them every 6 weeks, it is for the owner's benefit. The shod ones, once you go a week over a shoeing schedule, it has been shown that you do harm and there is evidence that supports that. So, the shod ones had to be managed. I think the information that came out from the BFBS and the registry was all quite good. What was also good was that the registration council put out a document that we could show the police if we were stopped, saying that we were designated critical workers by government. As long as we had our card with us, we could prove that we were farriers going about our business with the approval of government. I think that was a good thing to have in the truck.

AW: Do you think that horse owners were issued the same level of guidance?

F1: They were left high and dry. There was no cohesion in the ideas between yards- each one was making up its own rules. I think a lot of yards overstepped their remit, I think there was a lot of confusion and a lot of worry and stress. Again, it is difficult because each yard has unique characteristics to it. I felt that the slot systems worked fairly well, but it was done on each yard, and each yard proprietor set up their own rules. And then almost ruined them then by wearing gloves all the time- for example. So, they would take bits of information and not others. Being completely brutal I feel that Coronavirus has shown be that my bar of human intelligence was set relatively high. But what I've learned is that we are thick as mince. There was no logic in some yards. I wasn't frightened to have a conversation with them, but you couldn't change them. Some of it was fear driven, some was power driven. The psychological impact has been interesting. I was aware it was affecting horse owners. Certainly, recently with the coaching confusion that has been going around, competition rules, the split between England and Scotland in what we can do- that's all adding to the confusion. Our considerations have been pretty simple because it is about welfare. But the horse community as a whole has been let down in terms of good leadership.

AW: So it sounds as though you are saying that with your work being scientifically backed as being essential to welfare, it was relatively easy to justify decisions, whereas the things that horse owners do on a day to day basis is not necessarily considered as being essential to welfare- making it more difficult to justify.

F1: Exactly. It's like the owners of livery yards who were farmers who said, just turn it out. You can't just turn it out. Others offered either full livery of grass livery with no in-between. So, if you had a laminitic, obese or even sweet itch pony- those were your only two options. And it was beyond the yard's skill set or capabilities. No one thought about this when they took on a livery yard. For lots of businesses it

has been hard. For us we have been lucky. We haven't had to change that much. We run two vehicles now, we have PPE- but before we were washing our hands after every horse anyway. The only thing I added into my routine was alcohol gel that I used on arrival- partly to make sure I'm not taking anything out of my truck, but also partly for perception. To show the clients that I am your side, because if the client is relaxed, life is a lot easier.

AW: In terms of your apprentice, have you felt as though they were affected badly by this in his progression?

F1: Overall no, the worry just now is that the finals are in 5 months' time. He is meant to be at college in two and a half weeks' time, and they still don't know if they are going to college- and that is causing some stress- so no one is making a decision yet about whether or not it is going to go ahead. Certainly, there was meant to be an exam in May for guys doing their finals, and that has been pushed back to September. That's a massive hit and must have a big effect on their plans and how they are working towards their finals. They at least had 8 weeks' notice that it was cancelled, and they were able to adjust. Overall, its from our point of view – I had a chat with him about what he wanted from me over the next 5 months, what support he needed. What he has been missing- because we have been running the two vehicles is the random conversations in the truck – shoeing conversations/ farriery conversations. If we have just done an interesting job we will talk about that, and that will move on to what if we do that, and things that he might not need for the exams, but which is a good foundation to him being a good farrier in ten years' time. So he has benefitted from being out on his own in gaining independence in gaining independence and self-confidence, but he has missed out on the more obscure conversations you have which help to concrete technical, business and communication skills that make you a good farrier. So, he feels slightly remote. But there are fringe benefits that have come from it. Confidence and independence levels are higher for example.

AW: Do you have anything else that you would add that you think is important to highlight?

F1: The only thing I would add is that I think I have noticed fractionally fewer lame horses due to lockdown. For me, the really interesting thing has been the comment from a number of clients saying that their horse is going much better. I think that is due to them not competing, or not being able to compete. The pressure has come off them to get ready for shows, so they are less stressed when they are riding, and they are training less. I don't think many people will notice the combination, but I do think that at amateur and professional levels we can be guilty of overtraining horses to the point of physical or psychological discomfort- and then we try to do quick fixes. But if we trained less and better- and I think that has been shown by how horses have reacted to lockdown. The ones that have been able to carry on working, universally, horses are going better now than they were this time last year. They have been doing more varied things and less on the skills training. Because they aren't competing, they aren't doing the same training. They all do fast work as though they are going to Burghley. They don't have to be 5 stars fit. And the problem with 5 stars fit, we start to see horses breaking down. Even humans need to manage their schedule, and I think people fail to take that over from human condition, and they get hung up on getting that half-pass nailed- and they do it again and again. The horse fatigues, is resistant and, is probably setting up a repetitive strain injury. I think we will see less lameness's at the

end of the season, because of lockdown. But I do think the interesting bit is that people are commenting that their horses are going better. It was the same back in 2001- everyone was worried about fitness, but nothing broke. If anything, there were probably less injuries, because they had to modify the way they fattened their horses. We forget that. It is only noteworthy because so many people have commented in conversation. So, I think there is something in that for someone to measure- how people feel their horses were going.

Interviewer: Ashley Ward (AW)

Interviewee: HH1

Date and time: May 26th, 2020 19:00

Location: Aberdeenshire. Remote

AW: Our recording has started now, so to confirm that I have given you background to the study, we have designed this experiment to collect accounts of real day to day experiences of individuals that are managing horses, in particular horses that are prone to laminitis, during quite difficult times. So, I will analyse the results of these interviews and will identify the most significant factors that have made horse, pasture and laminitis management difficult. Eventually we hope to use these findings to inform policies developed in the future designed to inform of how to care for horses during a lockdown scenario. I have your signed consent form, and to reiterate, the recording of this interview will be stored securely and all the information you give will be completely anonymised. The results from this study are likely to be published in thesis form, and potentially in scientific journals. WALTHAM, the Roland Sutton trust and world horse welfare will also have access to the data derived from the study.

HH1: Yep, I am happy with that.

AW: Can I ask what your background with horses is?

HH1: Irish grandfather. My grandfather was a coal man, and for as long as I can remember he had what he described as ponies and traps. Used to have a cart horse which he took the coal out with and my uncles went out with a little pony and a cart with the kindling. So, I was around them all the time. Never thought to actually ride them, because to me they pulled carts. But that's basically where it came from. Also, he was very keen on a ___ ___, so he would tell me stories about the racehorses as they lined up, "That one's name is Trigger" or whatever, really he had 50p each way on something and he just wanted to watch the race. So that is where it all came from.

AW: So, did you then get your first horse, or did you ever work with horses?

HH1: Much later on. I was brought up in Edinburgh right in the city, and I had been trekking, and on outings and gatherings- I had been on horses and my uncle had horses, but he was in Inverness so wasn't handy. So, I was still kind of aware of them but because I was living in the city it wasn't really an option at that time. It wasn't until my husband got a job in Aberdeen and we moved up here, and horses are more common up here. You've got countryside and they are easier to access, so that's where I really fired up the idea about riding them. I must have been about 30 by then, so really riding them didn't come to me until I was riding them.

AW: That is really interesting that you felt that bond with horses before really riding them.

HH1: I suppose I had a lot of information about them from my grandfather, but I never really thought about putting it into practice. Sometimes, now things he said will come back to me so I suppose some of it must have stuck in my head. He was fabulous with horses, typical Irish, you know?

AW: Yes, that sounds like invaluable experience. Would you mind just walking me through the typical structure of your day, before the pandemic, and how you would normally incorporate caring for your horses in amongst your day to day life?

HH1: Well my life actually changed before covid because I retired from work, I was delighted! So, my routine had actually changed from getting up in the morning, seeing to the horses, going to work,

come back seeing to the horse's ride, whatever- typical of anyone who works and has horses. I keep them at home, which is one reason we bought this house. When I took to riding, I rode for a lot of other people. One lady in particular, she had 7 horses and did endurance and she wanted them kept fit. So, I did lessons and all the rest, and I said I'm not going to buy one (a horse) until I've got a house with land, and that's where all that came from. So, the routine that I had living with the horses, as it were, changed in as much as when I retired- I could then dedicate more time through the week, weather permitting -to riding. So, when COVID came along and the shut down happened, my life actually wasn't changed that much because my routine was basically still the same. I ride out by myself, I'm a kind of one-man outfit. I've got three horses and one person, so I'll take a different one out each day or sometimes two per day. So that is basically how I was filling my time, between that and socialising - going out socialising and meeting friends, because a lot of my friends don't work or are self-employed. The only aspect of my life that really changes was the social one. I wasn't able to go out and meet my friends, our conversations were via text or email or on the phone. So that was the only significant change. Until, of course, I lost my 27-year-old mare 2 and a half weeks ago, and that slightly was impacted by the lock down. It was an emergency, so vet came out, they've got strict rules about the owner has to handle the horse and you've got to stay a safe distance away and things like that- but nothing major. So, there was a slight impact there, but largely I haven't been badly affected by the lockdown.

AW: Do you think that the pandemic occurring when it has, e.g. in MAY, has made things easier?

HH1: I suppose it has, because I am able to spend more time outside and enjoy the weather, I suppose the timing is better. If we had been in lockdown during the winter, I would have been climbing the walls more than I am. I wouldn't have been able to go out riding, I would have been in and out feeding if it had been in winter- so I suppose the timing of it is probably beneficial.

AW: Could you walk me through your horse's daily routine and if that has changed with COVID?

HH1: They are out 24/7 and have a field shelter they can access as and when they want. I go out in the morning and feed them; they get a feed bucket twice a day everyday which I have always done. When I was working and seeing them in the pitch black, I knew they would come to me. What goes in the bucket varies depending on what they need, but they always get a bucket twice a day. So, if I was going out during my working life in the dark, they would always come to me and I wouldn't have to go searching for them to see that they are okay. I still do it, and it varies what goes in the bucket. Sometimes its just a carrot and sometimes it's a proper feed, which is mostly chaff.

So, they get fed, tidy up outside, check everyone is okay, go in, have my breakfast and then after I have walked the dogs, I'll take a horse out for a bit of exercise. I do my riding first=, then clean up outside. Teatime is about 6pm and again I give them a bucket and a slice of hay if they need it. But I can see them from the house and can look out the window at any time to check them. We are quite fortunate. The way the house is, its in a n L shape, and the long bit has windows in the corridor. From there if they are in the back pasture you can see their heads looking at you…

AW: With regards to the more heavy-duty tasks, like pasture management or maintenance, how are you finding keeping on top of those things?

HH1: Absolutely fine, as I said, I have always been a one-man band. We have four and a half acres which are split into three small paddocks, so I just manage them a little bit every day, a bit like gardening. Yesterday I was trimming dockens for example. Of the three paddocks, one is the good paddock, one is full of dockens and one is what I call the winter paddock because it is 50/50, it's not brilliant but its perfect for winter. The smallest paddock is the one with two fields shelters and the

dockens, I was going to get that reseeded and the farrier said don't you dare. That was many years ago and I am so glad that I never did. All I do is shut down the paddock… At the moment they have the winter paddock ad the rubbish paddock, but eventually as the grass comes in and they get fatter, they get less and less and less. That is the way I manage them.

**AW: Do you have a muck heap onsite you need to manage?**

HH1: Yes, I actually have three and I rotate them. When they rot down, the neighbours have polytunnels and all that rubbish, so I donate some of the rotted down stuff. So, I can set one pile aside until it is basically soil and it is fantastic for the plants.

**AW: So, in relation to your horses' exercise and feeding, do you tend to keep them in a lot of work and has this changed recently?**

HH1: yeah, it is basically the same. The other reason I bought this house was that it backs on to a private estate of 1200 acres of hills, and my house is on a similar sixed estate. The private estate my house sits on has a massive public walk, but it is too far away from the village for anyone to bother with it, until lockdown. I'll tell you a funny story about that, the walk goes along the river don, and for 20 years I've been riding both estates ad hardly ever see anybody. However, two days ago, I saw it all, quite literally. I was riding along, and I thought, is that man wearing a lycra body suit. As I got loser, my horses don't have shoes so often they are quiet. And as I got closer, I realised it wasn't' a body suit. A 65-70-year-old gentleman turns around and shows off his credentials. I said to him, "Are you trying to frighten my horse!?". Since lockdown, we now have naked ramblers.

The other estate is a bit more sedate- it has big hills and is a private estate with big hills and peace and quiet. This is fantastic for fattening horses. In fact, the owner of that estate had polo ponies, and that's how he fattened his horses – on the hills. It is very, very good for fattening. I have a wee programme in my head that we can do short fast work on one estate, or if I want to do more intense stuff it is more like a trek, and the up and down and change of terrain. The hill ponies love it, they are fantastic when you get them on the hill. Their sat nav is incredible. I have 2 Cumbrian fell ponies; the young one mother was fell father was warmblood, so he is a black and white cob. I tested his satnav the other day and he was very good. If you give them a loose rein they take off on a walk on a mission. If you are brave enough to trust them, they will take you where you want to go, they will take you home. But they don't allow for a field with a bull in it, or trees that are overgrown. It is really fascinating how they know instinctively where they are.

**AW: It sounds as though you have the fitness aspect all under control?**

HH1: Yes, that is why we bought the house, because of the riding.

**AW: Do you have any ongoing or new health concerns over your horses due to COVID?**

HH1: No. Nothing whatsoever. I am very fortunate, the minute you say that they come down with all sorts. I had Dolly for 22 years of 27, the other one is 16 and I got her as a two-year-old off the hill in Cumbria, and the third one I bred him He is 9 this year, he has been with me from birth. Having said that, the only thing they ever had, one had seedy toe and nothing else at all. No other health issues at all and that was up until I lost her at 27.

**AW: When you were speaking about your horse who was put to sleep earlier, and you said this did become difficult at times. Would you mind describing how social distancing was managed during this?**

HH1: it was actually very slick. The vets had a plan and had clearly thought about it. The owner had to handle the horse, which was fair enough, and we just had to maintain distance- and be aware of the

distance instead of standing next to the vet when they gave an injection. So, you were aware of it. When I phoned the vet, it was an emergency and they were out within an hour which was brilliant. So, I wasn't troubled by it, but I was aware of it.

**AW: Is this a similar case with the farrier?**

HH1: yes, the farrier is the same. My friend said it is they same, I haven't had the farrier since the social distancing, but she said you just have to keep your distance and you can't have a cup of tea.

**AW: None of yours have had laminitis?**

HH1: No, none.

**AW: You have told me how you have set up your paddocks to minimise grass intake, are you quite comfortable that everything is under control or are you quite vigilant over changes at this time of year**?

HH1: I am vigilant all year around for (laminitis). I have never thought of laminitis as being a summer thing. I think it is a summer thing because people take their eye off the ball. I have always liked to keep them with a level of hay as well as grass. If I feel they are getting chunky, or the grass is too green or there is more in the grass, I would rather give them less grass and supply hay, because I figure they will go for the grass every time, and if there is not enough grass they will eat they hay. Then I can monitor how much grass they are getting by how much hay they are getting. So, if they don't eat their hay at all, and their poo is green I would then watch out for how much they are taking in and think about cutting them back a bit. So that's kind of how I monitor it. I'd rather give them hay and it not be eaten to be able to know how much grass they are getting, because they eat the grass as soon as it comes in – so its hard to know how much they are getting. So that's how I manage it. I like to keep them on some hay. Obviously more in the winter if they need it.

**AW: have you had any difficulties getting hold of hay?**

HH1: I've got two good suppliers that I've had for years and they are local. When Dolly was unwell, I asked for the super-duper stuff and so that's what my lot are getting. They are now on rations of hay.

**AW: Could you tell me about any sources that you have received information from: regarding caring for you horses during he pandemic.**

HH1: None. I get emails from riding clubs and BHS send letters. But other than general information, I wouldn't say they has been any specific information -e.g. be mindful of the fact your horses may not be getting as much exercise- which doesn't affect me but my friend at livery was told that she could only be at the livery yard for an hour and that is restricting her to about 20 minutes of exercise. You are only allowed o be there for an hour and you have your tasks to do, so out of an hour I think you would be tight to get 20 minutes of riding, But that seems to be the general policy that most livery yards are adopting. In that scenario I can see why the horses would get fat. Because they are not getting the exercise. Plus, there are no competitions this year, and I know a lot of people who were fittening horses for endurance or cross country or whatever and I don't think they will get to the same level because they're isn't the same goal. So, I am lucky in that respect that my maintenance and exercise programme hasn't been affected so I haven't really needed any real input, But I think people like that would have benefitted form BE or whatever.

**AW: Is that who you would say the information should have come from- these organisations- or do you think there should have been something included in information from the government.**

HH1: I don't think the Government would be on point enough. I think the discipline you participate in would be better placed to give you helpful tips to break up the monotony- even playing games with your horses to get them away from eating for a while. Because in a lot of cases horses will have been parked in the field and will have been left there. I THINK THE endurance lot or eventing or whoever could have been a wee bit more forward in coming up with some ideas. – But there has been a lot on the internet- a lot of the forums are good for tips and things.

AW: would you say you have any concerns for the equestrian industry as a whole?

HH1: I don't think so, but I think I am in a good situation, because I have everything close at hand- so there is nothing that worries me because I am not really affected by someone else dictating what I can and can't do.

AW: Is there anything else you think is important that we haven't touched on yet?

HH1: I mentioned a third pony I got in from my friend. Basically I needed a third horse when I lost Dolly to break up the group, so I got her , a fell pony, which has been in a field for three years, She had been put into a field with sheep and lambs, which was a fantastic field for fattening up the sheep and the lambs. My friend lost her father to covid, he was in his 80's and the family had been nursing the father and supporting the mother at home before he was taken into hospital where he subsequently passed away from the virus. But she had acute asthma and had to self-isolate herself, and the horse had been in the field -lush green field wit the sheep. I had been having a discussion with her and she was worried about the horse and I lost my pony as well within weeks, so I said I am happy to take your pony indefinitely and stick her in with mine At least you wont have to worry about looking after or exercising her or whatever. So that was a result of the epidemic. – It was in a field mile away and she would have to drive to see it- she was worried about her horse and with the way it turned out I took her. When I got her home, she was like and elephant, it was like someone had pumped her up. She was getting scoffed in about the sheep's feed, and they were getting barley and all sorts. So, she moved in with me, and she now has zero. She doesn't like the lack of grass. She is quite funny, because she is the same breed- they are identical- they are girly and squeal – like peas in a pod. The nature and behaviours are exactly the same. The only thing is the male gets very jealous- of me- so I can't make too much of a fuss of her. She is very cuddly, a nice pony, but he gets very jealous.

AW: Do you think that your friend will take up horses again?

HH1: I really don't know at all. We have left it as an open loan at the moment. I am into showing and her pony is not a show pony, if I was to buy one, it wouldn't be that one, so I don't know. But she is fitting in quite well with my two, so I'm just going to keep an open mind. Once the lock down is over and we are back to normal then we will cross that bridge. By then I'll know what she wants to do about the pony, because she won't get another one. But I'm not going to look to the future at the moment.

AW: have you been disappointed by the showing cancellations?

HH1: yes, because they were looking their best. My youngest is looking cracking at the moment because he has been up the hills like no one's business and is superbly fit at the moment. But it means we get to keep the trophies we won last year without doing anything. And he can keep his whiskers!

AW: Is there anything else you think we should discuss before we finish up.

HH1: Would you like to come out and see the place for your study? …

END---------------------------------

Interview 1

HL 1

**AW: Could you lay out how you normally incorporate looking after the horse – before the limitations of the pandemic?**

**HL1**: Okay, for me what I do is first thing in the morning I would do a, "drive-by", before the pandemic there was no need to feed him more than once a day. So, I would get up early, get in the car, (his) field is conveniently located so I could drive past, check from a distance that they are okay. Then I would finish work at 4pm and go straight out to the yard, depending on what we were doing that day, dependent on his mood- riding depended on lots of things. But the standard routine is in, all over check, looking for anything abnormal or different. In, fed, exercised and then his main feed, and then turned out again. He is not quite ready to be stabled, probably in winter but that depends. That is my Monday to Friday routine. Then at the weekends I am there all-day sorting things out and doing extra bits and pieces. The only things I am strict with is I do weight out his feed and Wednesday is weigh in day- to keep an eye- being that he is such a big horse, things tend to happen quite quickly and everything is exaggerated- he can lose 20 kg in a week- so I like to keep an eye on him just in case.

**AW: How do you monitor his weight?**

**HL1**: So, I have a weigh tape which goes up to 1100kg, so I do it by the weigh tape. I do a little bit of body condition scoring as well, I just recently had a chat with a nutritionist just to make sure I was doing the right thing and checking the right places. He got a score of 5 so I was quite happy with that. So, I just check, I know because I touch him every day- so I am checking loins and neck and withers to see what is different about him. So that is on a Wednesday. I also use my lunge line with little bits of tape on it so that I can see if there are little differences. Also – the same thing with his girth- because his girth is non-elastic and it isn't leather- so I know when I am tacking up too. Just last week or the week before I had a session with a nutritionist, and I had the phone set up in the stable and we looked over him as best we could. I do feed Spillers as well.

**AW: Was there anything before lockdown- that was a medical concern in particular?**

**HL1**: Well this horse is quite new to me, so from previous experience I am looking for lameness which he has had on and off since I got him. I am looking at how he is stepping, are strides shorter, is he taking smaller steps, or is he footy? I am also checking his eyes as I have had issues with eyes in the past. He has naturally got a worried look; I'LL do a capillary refill on his gums to check that he is hydrated. I have had experience of heart problems in the past, so I am always assessing his groans- are they complaints or is something wrong? I do listen to his heart- I wouldn't be able to say it was irregular, but I have a ballpark of what it normal for him. The main thing is to know what is normal to be able to say what is different. Lumps and bumps as well, I am always checking to see if there is a reaction for something. It's very quick- it's a routine. We do hoof oil maybe 3 times per week. I'm a little obsessive, but from experience when you have something go wrong. Also, in the yard, the things that go on with other horses- you start checking, "Is that normal for my horse? ". It is natural when things are going on you start to look for it in your horse.

AW: That all sounds very thorough and sensible. Since lockdown has happened, can you tell me in what ways your routine has changed?

HL1: Yeah, so I don't do the morning checks Monday to Friday, so another person is there 3 days a week in the morning- so I ask her to cast an eye over so that I don't have to do that journey. I would go if required, but I have reduced the number of times I go to the yard. I am a creature of habit, and usually at the same time most days, people know I will be at the yard. We haven't set any time structured rules, for time at the yard, but I would say I am the sole party doing the disinfecting of all of the gates.

Aw: I see. So, the yard hasn't put any measure in place that liveries have to adhere to specifically?

HL1: Well yes, we did put out a note to say, "No extra people", so one person per horse for liveries. And where you can, share. So, a couple of people will maybe come in the morning and do two horses, and the other will come at night. Those are quite natural rules. The main thing is not having extra people at the yard. We have also said no to the sponsors coming, because the liveries and the "helpers", we would always get the horses in for the sponsors, so we have asked them not to come because we didn't want to have to be doing that- and dealing with people on top of what we were doing already. So, a few rules, with sensible exceptions and adjustments as and when we can.

AW: That sounds quite sensible. In terms of the health checks that you like to perform, do you feel like you are still able to do what you would like to do with your horse in a day?

HL1: I am feeling restricted, so I am still able to do all of my health checks. For me, not irrespective of the rules, but that is top priority. For me the restriction has been on riding, purely because our forest, the forest in which we can ride, has become very busy with people who are not used to horses. Do yeah, I think there is a definite restriction with what I am able to do exercise wise, with my horse. But taking the positive from it- it has forced me to do more schooling and things which is good. But that would be the main restriction. Also, people coming onto our land, due to our direct access into the forest. That's the main impact for me, is other people.

AW: Have you been able to access the services for your horse that you would have been able to before?

HL1: Not without a fight. I haven't been able to access my saddle fitter- but she is on maternity leave anyway. So that is a mild concern. I am okay with gullet changing but flocking and things, I am a little concerned with the saddle. There is not a lot I can do about it. My saddle fitter is not available although some are. My physiotherapist is also pregnant, so she has categorically said no until August. She is very much- only if a vet says that your horse needs it will she come out. I am very respectful of people's choices and what they need to do. With the vet, the horse was due vaccinations, and I didn't want my annual vaccinations to be in December, so I had schedules it al out to make it earlier in the year. So, with battle from the vets, I had to ask a number of times- I need you to come out and do vaccinations, you're coming out anyway. I was able to work with them eventually, through coordinating with the other horses that needed to be seen. Then by that time, he needed his teeth done, he was overdue, and the poor vets eventually said we will just do it, to save arguing with me really. The farrier as well, so both the vet and the farrier have set their own- please social distance in the yards. But sometimes people want to come and see what is happening- so they have said that you are responsible for keeping the two metres for yourself and everyone else who wants to come and

have a nosy. The farrier said could you pay me by bank transfer instead, and just tie up the horse and leave me to it to keep the two-metre distance. So, it has not been terribly inconvenient really. It is just a bit different.

AW: Have you found accessing information from each separate provider easily, regarding how they want you to handle social distancing?

HL1: For the farrier it has been intuitive, but for the vet is has been unclear. They were putting up public posts saying this is what we are doing. They eventually sent out a letter to everyone, but in the meantime, I was discussing with them vaccinations and lameness, but then things like, the vets would post "OH this puppy came in to the practice for its vaccinations", I'm thinking you have said no to me about coming to do my horses vaccinations, how is it different to come and give my horse his vaccinations. They weren't consistently clear, and their updates weren't very timely. We get the government announcements, and everyone deciphers them as they want. And the time between that and putting out information was just too long in my opinion.

AW: In terms of other places that you might have got information for, how to look after your horse during this time, has there been any other organisation that ahs stood out as providing useful information?

HL1: I think I have had to seek things out. I am not a big lover of the BHS, but they made signs or something. I didn't put a lot into looking at what they had to say because I think the differences between England and Scotland add an extra level of complexity. I looked at the government website, but as far as our government bodies, I can't really think. I am thinking of who is in my feed that I get updates from, it seems that everyone has interpreted it in their own way. Some riding schools are saying no lessons, others were doing one on one lessons. It felt like it was every man for themselves and whatever someone declared they were doing, was okay.

AW: What, in your opinion, would have been the best way to share information regarding how to manage horses during the pandemic?

HL1: Well, I think probably on a social network of some description for all of these companies. Even the feed stores and things, to say this is what we are doing. But with clarity in the message, and the timeliness of that message- in relation to as and when we got information from the powers that be. I would be impractical to phone or send out letters, but I think a social media platform is a good way to put out a clear message- and the same message across the platforms. That would have been helpful.

It was quite interesting that the farriery council, it was very clear that they had written something for all of the farriers to post on their farrier pages. So for our farriers, they all had the exact same wording. So I am guessing that somebody from some governing body has said to them, here is an example for something you could put up, it would be great if we could all do the same. So that was good because it gave the same message across the board.

AW: That sounds great, do you think that gave more credibility to the message, knowing that it came from a place of authority?

HL1: Yeah definitely.

**AW: In terms of laminitis specifically, has that been something that you have worried about with your horse?**

**HL1**: I don't see it as a something that would impact him, however I will always treat him as if he could get it. Low sugar diet, checking his feet, monitoring his grass intake where possible. Same for ulcers- I always feed just in case they had them, so that whatever I am feeding wouldn't contribute to the problem. Suffolk Punches are not prone to it, but that doesn't mean he isn't going to get it.

Aw: Would you say that your preventative strategies have been impacted by the pandemic?

**HL1**: In a round about way, yes .It has been a little difficult to get feed, so I have had to make some changes to his diet. I don't like to make drastic changes, but for the 3-4 places available to us saw panic buying and not having stock, which meant I had to make a change from one brand to another- essentially the same product so it is not a big concern. But it is something I would choose to avoid normally.

**Aw: For the overall equine population, would you say that you can see that the pandemic will have impacted laminitis management?**

**HL1**: Yes, people's availability to get to their horses. So, for yards, so I have read on yard groups, some yards have banned the liveries completely- so you are absolutely at the mercy of the yard owner to manage your horse and however many others in a way that is acceptable. Especially if you had one that was prone to laminitis, and your strategy was to keep them in. We have one in at the minute for laminitis, but we are fortunate that we have enough people around to make sure that she is watched over, she isn't standing in a dirty stall, and that we aren't causing more issues. But for people who don't have that luxury, I think it would be a nightmare to manage laminitis. Feed restriction, and what are they getting, and how much are they getting. If it was me, I would be weighing every gram of hay and I would ensure the hay was soaked on a timer, and I would have it regimented. So, for me, it would be an absolute nightmare.

**Aw: Are you worried for the industry as whole, in terms of recovering from the pandemic and the way that situations have been dealt with?**

**HL1**: I think on a feed store front I think people have damaged their reputation by inflating prices, because they know people are desperate. I think reputationally, there are a couple of stores that I have written off- I'm sorry but if you're going to behave like that, I am not going to shop with you. I think the people who are self-employed, saddle fitters, physios, I worry that all of a sudden, they will have no income and will have to look at doing other things. Maybe selfishly- but them I am out of a saddler and a physio. It is worrying. People have got to adapt. I think with the vets and our authority's that are available to us- some have just not painted themselves in a very good light because of the ambiguous information they have been sharing, in my opinion.

**AW: With regards to management of pastures and turnout, do you think that the pandemic had an effect on your ability to enact any procedures that you would have normally?**

**HL1**: That's easy because we have no procedures, so there is no impact. But I can understand perhaps if horses are turned out more, because they owners cannot come up and deal with them every-day, then there will be an impact on the fields because of that. There is just no process for us, so it makes no difference whatsoever. But I can see how it could for others.

AW: You take quite an active role in the sanctuary side of things. Have you seen an impact of the pandemic on sanctuary horses and they way that it is run?

HL1: Yes, we have a few key laminitis every year group, that I think because people are busier managing their own horses, and farriers and vets, those that should have been checked more regularly are not being checked. Also, to be fair, people want to go out and ride, the laminitics are not a priority as much as they have been in previous years.

AW: Do you think it is achievable to sustain the management of social distancing and increased hygiene in addition to all of the yard duties when things are busy anyway?

HL1: I think it is achievable within reason for a period of time. But I have definitely noticed that at our yard, people have sort of given out. People used to wait until you had come out of the feed store before they went in, and now it has changed to, just come in we are fed up of it now. For cleaning and disinfecting, that is just my personal mission on the yard because I feel better that things have been cleaned. But it is not feasible to sustain it- that is my entire Thursday night every week. You get quicker but it is an additional thing to do. The social distancing is an additional thing to do which has been glazed over. In theory you think it shouldn't be a problem, but in practice it is very inconvenient and leads to people rushing and getting frustration. It is not unachievable, but it is very difficult.

AW: I think that is an interesting point. You have provided a lot of really useful information. Is there anything else that you think is important for us to talk about?

HL1: I can't think of anything just now, but I'm sure I could if I thought about it more!

END---------------------------------

Interviewer: Ashley Ward (AW)

Interviewee: V1

Date and time: June 3rd, 2020, 15:07.

Location: Aberdeenshire. Remote

**Informed consent was confirmed; however, the recording was mistakenly not started.

AW: Can I ask you to describe any challenges that you face in your delivery of veterinary care during the pandemic?

V1: I guess it has been quite hard trying to stay 2m away, I say that's probably been the trickiest thing. In terms of emergency calls, not so much on the routine stuff, for colic's or euthanasia's- that has been quite hard. Purely because often you end up in a situation where you need someone to hold the horses, and you feel like you would rather take your chances with coronavirus than get crushed or kicked at that precise moment in time. So that has been hard, although it has definitely not been for minutes at a time.

AW: Job satisfaction is such an important thing in your profession, would you feel that you've been able to provide the standard of care you wanted to, or have you been left feeling that you couldn't do what you wanted to because of the restrictions.

V1: Yeah, I'm fairly happy, there has certainly been a few cases where normally we wouldn't prescribe medication without having seen the horses, but there have been a few situation where people have rung up , but they have been owners that I have known already and they are quite sensible. You can trust their judgement on it. There have been a few situations where I've ended up over the phone, and they have maybe sent in videos or photos and I've prescribed on the back of that. Then I have obviously followed it up a few days later or a week later to see how things are going. There has certainly been quite a bit of that. In the past they would have been automatically booked in for a visit/ But touch wood, most of them have been okay with that. It's always seen if things aren't improving. Just in very select situations.

AW: Would you say that you have been able to take care of yourself, and access everything you need to be able to take care of yourself?

V1: I've had to be more organised, and I am definitely having caffeine withdrawals. Normally I would just stop and get a coffee somewhere, but obviously I can't. There have been some occasions where I have had to go into client's homes to use the toilet – that is probably the worst one! Whereas normally you could stop somewhere are get a coffee and nip to the toilet- or go in and out of the practice. But that hasn't been possible- which is less than ideal. I guess I could have gone on the verge… although I have been wearing gloves!

AW: It appears in human medicine, the way that people are managing their patients has definitely changed. As we come out of this, they may change their practices with regards to telephone conversations- which have been quite effective in some cases. Do you think that could be similar for vets?

**V1:** No, I don't. The problem is that our company is really keen for us to do phone consults and videos. The problem is that we already do a lot of that in that people will quite often be on the phone for advice at least two or three times a day. We don't charge for that, so it is kind of hard, because it is the same situation as before the lockdown- you feel like you couldn't really charge for that. Obviously in those situations when I prescribed drugs, and it has been okay- in an ideal world we would do that. To be honest we probably won't because financially the company will want visits. We do have a charge on the computer, we were supposed to charge, I don't think anybody has actually charged for a phone or video consult because we do it all the time anyway. I do think that if it wasn't a business, I totally do think that we would probably prescribe things like, maybe some antibiotics, but more things like ventipulmin for coughing horses and things like that where normally we wouldn't.

AW: In terms of laminitis, have you been receiving more calls than you usually would have?

**V1:** Yeah. We have had heaps of laminitis this year. I'm not convinced that it has been linked to coronavirus- I'm not sure that it is that people have suddenly turned their horses out for longer because they can't get up to the yard. I don't think that is the case, but certainly this year we have had heaps of laminitis and heaps of colic. Normally we would expect to see lots of colic's in the spring and autumn, and obviously laminitis in spring and autumn. I would say that this year we have seen way more than normal. Both of those things could be linked to management, laminitis I guess more in relation to grass growth and it could be management changes. Certainly, as a practice we have definitely seen way more of both of those two things. We have actually been going out to them.

AW: Has the way that you treat these cases changed since before lockdown, or are you still using the same guidance as before?

**V1:** At the beginning of lockdown, I probably went out and visited less cases. We were getting phone calls about it, and in a normal situation I would have gone out and visited them, but there were a couple that were on the cusp, and I just advised them over the phone and dispensed danilon. There was one ongoing one that I had seen and x-rayed before the lockdown, that one I just kept in touch with the owner weekly- whereas before lockdown it would have been booked in for visits. But I am happy that because I had previously seen it a few times and it had been x-rayed before the lockdown. I probably would have booked it in for re-examination to gauge things myself, and that one didn't get that. But the owner was pretty on the ball, so that was okay.

**AW:** Have clients been adhering to what you have asked them to do for the most part?

V1: Yeah, I think they pretty much have taken what I have said. The ones that have had issues with the amount of turnout or bringing the horse back in, are ones that would have had that same issue even if coronavirus hadn't happened. It is people that for whatever reason can only get up once a day and are not willing to pay for full livery.

AW: Do you have any concerns overall with the horses in the area that appear to be associated with the pandemic?

**V1:** No, I don't think so. I heard a farrier saying that he was more concerned for the owners, because some yards had done a complete lock down and he was worried that the owners mental health would suffer! I think in terms of the horses in the area we have been quite lucky- with our clients. To be honest it is more the other way- you would go to yards in the first three weeks, and you're like- are you guys aware that there is a pandemic on? Everybody and their mother are up at the yard, "Oh great!"

**AW:** That is interesting- I was going to ask if you think that owners have been aware of how to manage vet visits and how to manage social distancing.

**V1:** It has been tricky. I', probably not the best in that I could have done more. I have been trying to wear gloves, and I will make a comment to remind people about the 2m distance. I could have personally done more; in that I could have worn a mask- and that could have been more obvious to people that I have been taking it seriously. But then that is difficult – when you get in a situation- like when you are doing teeth and you need someone to hold the horses head up. So, I think I am a bit lax on those sorts of things. At the beginning of lock down I was certainly surprised as how many people were there. Some yards have taken it very seriously and no owners are up, then other yards where everyone was using it as an excuse to come up and visit their horse. So yeah. I guess that is probably similar across the whole population.

**AW:** Do you think that aspect of equine practice was acknowledged by the organisations that gave the instructions on how to manage cases?

**V1**: I think the problem they were in is that we couldn't have said no to doing emergency work. You're just basically going to have to get on and do it. I think there were probably ways that we should have protected ourselves more- like we are going to have to put this horse down, or it is violently colicking and I am going to need someone to take hold of it- and because of that we are all going to need to wear masks to reduce the risk slightly. To be honest I have had some horses react just to me wearing gloves- so I haven't actually tried it with a mask. You know, usually the ones where you are going to need someone to hold them, and you walk in and they are already suspicious. My feeling is -some of them I have had to take my gloves off to get near them. So, I think that is just quite difficult. To be honest probably BEVA and the RCVS have had to glaze over the top of that and not really address it in too much depth. I've certainly found it hard to say- yes, we will come and do emergency work- and not to do routine stuff when you're there- because the routine stuff is easier to distance yourself. One of the ways around the emergency stuff was to bring a nurse with us. My personal opinion was that you're basically just adding another person into the mix- so you are potentially going to just expose another person. Or that person might be carrying coronavirus- so who knows. If you are in the middle of the night and you get called out, you're not going to wake up and pick up a nurse to go to the call.

**AW:** With regards to laminitis specifically and the way that you manage acute cases, have you felt that that it has been manageable to stay within the guidelines?

**V1:** Yeah, I have probably not booked in as many for re-examines. I have relied more on ringing them to see how they are getting on. Otherwise, I guess the other thing I noticed was one of the horses I saw for laminitis, his shoeing was overdue by two weeks because the farrier was trying to lengthen the time. So, there was a question mark over that as something that was contributing. We also had a horse here for colic, but its feet were bad, and it was a slight concern. It never actually got laminitis, but her farrier was shielding, and she couldn't get a farrier, so we had to get a farrier to come in and do it at the hospital. I think all the others that I have seen, the farriers have still been coming out. There has been one farrier that has been shielding locally. I have seen two horses from him, one was coming up for shoeing and obviously they have had to use a different farrier now. That has had a bit of a knock-on effect, but the other farriers have all still been managing.

**AW:** Do you think it would have been more helpful for the farriers to be working from the same guidelines as the vets?

V1: No, they have been okay. For the most part of the farriers we have seen, one has been shielding, one has lengthened the times between shoeing's- which has maybe contributed to one case I have seen. The rest of the farriers have been carrying on – but have been keeping the two metres and carrying on. So, I think they have probably been, well I have probably been too lax, and I would say have been on par with us All of the ones I have chatted to have been pretty sensible.

AW: Is there anything else you think would like to add?

V1: Not really.

AW: Are you coping personally?

**V1:** Yes, with our work it has been frustrating because some people have been furloughed and so on call has increased but we are obviously not being payed as much. Silly things like that. It is fine because we have still got jobs. Everybody knew the turnover would go down, of course it is picking up – but I'm sure they won't tell us that!

Interview 1

WCM 1

AW: Could you give me an overview of how your role has changed during the pandemic?

WCM1: Well, to be perfectly honest, at lockdown, we had just taken in quite a few large cases, so we were well and truly overstocked. Our normal, happy, stocking rate that we can deal with -with the main aim of rehoming them as soon as we can- at the start of lockdown we kicked off with 98 horses. So, a little bit overstocked. The types of horses that had to come in were your piebald cobs, that were either abandoned or were on rough grazing, and I have to say this was South of the border, it was just that Belwade were not able to take that number at that time. Many of the two groups there we, 11 foals were due to be born, so we had mares heavily pregnant, and we had colts as well, which we just took 10 of. Now, the main is the rehoming, so the others that we were able to rehome, we found rehoming has actually gone very well. Although we had folk come in, and some returns, but not as many as we though we might have due to the pandemic so far, it has only been one or two, where people have been quite upfront and have either lost their job and they want to be able to act then, rather than getting themselves into difficulty. So, we had a few of them, but you can count them on one hand really. So, our stocking level now is sitting at 85, with two foals still to be born. Of those big cases, they are all more or less semi-feral- so that has all the implications that come with that. Because we have kept the yard staff on, of which there are 10, we started off in smaller groups- where we isolated girls into different group and each group kept to their own little nucleus. So, they could all see the horses on the farm- they were all on basic care. As long as they were standing, breathing, eating, drinking, that was all- at the beginning- we thought was a good way forward. However, about a month and a half – in May really, we decided that some of the semi-feral ones and some that were actually handled, were turning into semi-feral. They needed to be worked, they needed to be handled. So, then we decided to open up the whole group to work in their normal routine. A lot of them have come around and they have been able to get the handling that they needed, which was great, because we were then able to open up the rehoming side of things for companions and youngsters.

AW: Okay, that is really comprehensive. You mentioned that your group of colts needed gelded- was that organised easily enough?

WCM1: Well, luckily, we had organised a team of vets to come in and do the group castration before the lockdown. Along with the vets, we saw that as a welfare issue, and we saw that as essential. So, all 10-11 were castrated all in the one day, which was good fun, but the job really needed done before more mares came into season. And my though was that if foals began to appear, we would have lots of mares in foaling season and those poor boys would be driven insane. The one thing we were able to do at the beginning of lockdown was getting as many of them out into the fields. As you can imagine I didn't really want stallions running around with colts. So, as far as the horses were concerned, they were relatively easy, however you had to do a blanket form – look at the big picture, see how they were doing, and if there were any individuals then deal with them as such. But luckily, we were okay.

AW: It sounds as though you were well organised, how did you prioritise handling?

WCM1: well, only very few had to be individually picked out. A lot of them are black and white up at belwade, and they were, as far as weight wise, some of them were pretty ropey, but they were okay to transition on to hay, not haylage, later on we introduced the haylage. We didn't have much grass at the

time, which was good. So, we were able to control the amount that they ate at the beginning. Naturally, we very rarely hard feed horses here, because we find so many that are either worm burdened, or they have never been wormed, so we had to get handling procedures into place so that we could do that- whilst leaving them based in the field. We have a big barn that splits into four, so we can actually deal with individual herds at any one time. We would have up to 30, depends on the size, but they can be sedated and dealt with in a group- and then we can pick the ones out that we need to look at more specifically. So yeah, we had to deal with the worming requirements, the dietary requirements. They came in without having too much anyway, and emaciated horses are easier to deal with than the obese horses, so actually it is easier to help them put on weight. But we just keep into the low fibre diets. There were some skinny mares, but they were feeding foals in-vitro so that is totally natural for them. So, then the foaling- it is a lot easier to foal a thinner mare than one that is obese. And all of the foals have been born outside, we don't pay any particular attention to them. We just let them get on with it and don't interfere unless it is necessary. And that is the way I do it here anyway. With the others, it, they all came, the ones that were already here, we have had such a mild winter, so they have been living off rough grazing. There were only, about two fields that we actually hayed up during the winter- and they were for the thoroughbred types.

AW: Have you had any issues with accessing your farrier during the pandemic?

WCM1: Well, the first week, whilst we were figuring this pandemic out. But after that we see it as essential, so our farrier has been out as usual.

AW: Have you noted any changes in the welfare calls and concerns during the pandemic?

WCM1: Well, most of our calls go through the head office, but the last meeting I was in said our calls have risen significantly. But I couldn't tell you the numbers. From what I have been told, and yes, I have had conversations with people on the phone here, I wouldn't say it has gone up, but certainly the big picture- UK wide- it has. People looking for advice. We did put out, along with the BHS and the BEF and all the other people NEUK, you know, help and guidance on the internet about how to deal with your horse, with the pandemic. We suggested that if you can rough off your horses, do so. Turn it out, get it back to the basics, rather than trying to make yourself go to your horse twice or three times a day. Try to have a buddy system. That went down well for some but didn't go down well for others. Because the individual horse owner- as you can guess- were saying, oh but my horse needs this that and the other. Maybe I am being cynical. But I suppose you can understand that, especially if you have a horse at livery. That did settle down, but it was interesting how many people were against that turning out. People were saying, oh my gosh, my pony had Cushing's, it has laminitis, it has this that or the other. And I thought, that is interesting to know how many people have ponies with medication for these things. It would be interesting to see how many people are actually coping with this. When we got that information out, we said- if it is possible, do this. You know, you are trying to save your own life first. Not the ponies because they are a bit more resilient that we give them credit for. But then it is individuals.

AW: And from the cases that you usually see in Scotland, did you have any particular concerns when you heard the news about lockdown and with the pandemic?

WCM1: Well, I wondered how people were going to cope. I thought, are people going to go away and forget about them. But then, they wouldn't be any different to any other year. I mean, talking to the two field officers in Scotland, they were only going out for what we call P1's- so that was emergency cases only. Not P2's, which is advice and concerns. Once the initial few weeks had passed, then we did get more concerned about fat ponies – but is it more than any other year? And in this area- yes. Right across the section from skinny horses, right over to the obesity. And the obesity people are the ones who do not want to take advice- they cannot see any problem, and that is the key issue.

AW: And in making the decisions that you have made during the pandemic, is there any particular guidance that you have been following?

WCM1: Well, I mean we have obviously had to follow the government guidelines, as such. But the organisations have clubbed together over the time. So, you have NEWC, the National Equine Welfare Council, of which we are part of. That is basically equine charities which have all come together. So, they can put out blanket comments and advice, for registered charities trying to encourage people who are collecting horses and they are also trying to get a reward for doing it, then they should be encouraged to become a charitable entity. So, you have got the likes of the Blue Cross, Redwings, the horse trust, up here in Scotland you have the SSPCA and us, and redwings. So, they can all come under the one banner. So, although we all work individually, we all know what each other are doing at one time. Obviously, the British Equestrian Federation, and BEVA, the veterinary advisory groups as well. So, our head office keeps abreast of exactly what they are going to issue, and we have to follow their guidelines.

AW: Many people have highlighted the BHS as the primary source of information, is that an organisation that you would also be looking to?

WCM1: Oh yes, the BHS is part of NEWC as well yes. And the BEF. In Scotland we work pretty closely with BHS Scotland. So, we try to sing off the same hymn sheet as much as we can. You know, if BHS Scotland were putting something out, then our guys look at what she is putting out and we play into that. And they asked for our ideas as well before it is all issued. But generally, they are pretty good on their advice. They do a lot more on social media, I mean we do as well- we have lots going on- on Facebook and twitter and everything else, and that is getting more and more because we are all getting very good at this virtual stuff. We are getting out messages a lot easier than we did actually.

AW: Do you think that will continue then- this revitalised social media?

WCM1: I do, I think it is a very good tool that can be used. You've got to watch it, because individuals can get a bit too involved, but when the message is out there, I think more and more people are using it and it is a very good tool for education. I thin for your general horse owner, why would you go to World Horse Welfare? So, it is not top of the list, because they don't have a problem, so they are going to go to the BHS because it is for everybody. Well we are actually for everybody because, what actually constitutes a welfare case? So, yes for the future- that truly can be utilised more, we are looking at a lot more. We have been doing this even with out international work- I mean we are working with 15 different countries across the world, and we would normally have had teams go out there in person for educational visits. Of course, that has stopped, but we jut can't abandon them. So, your virtual stuff,

like webinars and things like that, have been tremendous for those communities. From Cambodia right over to Columbia.

AW: In your opinion, is there any information that you think should have been released to help owners?

WCM1: Quite a lot has been done. With the BHS and ourselves, I mean, we are dealing with something that is the biggest killer of equids in Scotland. People think it is being emaciated, but it is not- it is laminitis. With the laminitics, people don't realise how quickly it can come on in a pony or a horse, or a donkey- and it is not just a typical section A, every single one of them can be subjected to it. Because people think it is just your fat ponies, but there are so many factors. So, I think more education on what triggers it could be done, but it can get very confusing- so you do need to keep that message quite simple. In my opinion. Some people, individual horse owners, have lost the sight of what a horse should look like. You know, they see the likes of the heavy-set Highland pony, big crested, carrying far too much weight- and then they think for the betterment of the pony, they think that is normal. I am critical of them. Although a lot of people at shows etc, have addressed this issue- what is a highland pony and what should it look like? You know? Go back and look at your studs. When you see them coming in, the laminitics, and in this pandemic situation, if we were to bring in laminitics- we would have to make a serious decision on- are we going to continue with this horse or pony or not? And with so many chapping at the doors, more likely than not, we wouldn't have to luxury of pulling everyone through it. And as we know, we can't pull everyone through it, we are not god. And with the triggering effect of obesity on laminitis, can cause so many other issues that we will call it. You know, sorry- you are laminitic and in an ideal world we might be able to give you a chance but with so many at the doors we can't.

AW: Is that a decision you have had to make during the pandemic?

WCM1: Yes, we have. Just the one. We are about to take another return coming in with a history of laminitis, that hasn't been dealt with at the right time. So, although I haven't assessed it yet, but the chances of it staying on the farm are pretty slim.

AW: That is completely understandable. I have heard reports of individuals keeping laminitic ponies for extended periods and you do begin to wonder about how ethical that is.

WCM1: Yeah, well, you're going to get me on my bandwagon. You know, if I was to say- euthanasia to me is actually success- because I have stopped further suffering. We don't euthanise for the sake of it, we only do it for a reason. I am a bit cynical in the way that- that horse doesn't have a diary and it doesn't know what it is going to do next week. It doesn't even know what it is going to be doing tomorrow, it is an animal of habitual nature that has been institutionalised. It knows it is to try to eat for 16 hours a day, so that is what its going to try to do. Take that away and try to manage that… is that really fair on the pony? I have heard and seen people who have been doing this for 6 months, and that pony has hardly moved. And you think, that is so not fair. And you think, then what? It is going to wait for the next bout! For me, and I should be always trying to save everything? No. Euthanasia can be a success- I have stopped it from suffering. In terms of rehoming- can I get that pony to the stage where it is going to be monitored for the rest of its life, and can I ask someone to do that? There are people hwo are happy to do it. And they are aware of the complications. We have managed to get a laminitic through the initial bout and that it great- and quite a lot of them do, but we will have to make sure the

home is absolutely correct. That horse or pony will need monitored, it will need a good intake of the correct feeding and we need them to keep an eye on the weight. And make them aware of that. But as we know, even the best person at dealing with that all, can be caught out.

AW: And you said you took in a lot of horses at the beginning of the pandemic, is there a similar ethical question there in terms of behaviour?

WCM1: No- we all work off of the same hymn sheet- we have to. If we have the luxury- we might give someone a bit more time. But we have to ask ourselves, is the animal going to be rehomed with a good quality of horse.

AW: So, we were definitely concerned about horses and ponies and the impact that the pandemic would have on them, do you have any overarching concerns?

WCM1: Well, I think that concern is probably more South of the border where more people have their horses on livery. You know, lets face it, up in this neck of the woods, more people have their own little plot of land, or their livery yard is quite close. So, I think people, like livery owners, with the guidance they had been given, livery owners were making sure their yard was alright. And I think the BHS did a great job of that. To make sure that livery yard owners, A) realised that they have a duty of care to the animals too- so the buddy system I think helped a lot. But what will be interesting is when we come into August / September- we are expecting trouble then. I think we will start seeing a lot more people not being able to look after their animals. There is a couple of ways of looking at it. More people are aware that there are different ways to keep your horse, you know, you don't need ten million rugs and 10 different supplements, actually they don't need it at all. As long as they have got fibre going through their bellies, they have an inbuild central heating system. They utilise that, and the more rugs you put on an animal, the worse it is because they can't convert all of their carbohydrates and sugars- and they are stuck in their stables. But if they are able to move about, they can convert it a lot easier. So, that is going to be a bit of an education thing- to try to get more people aware that you don't have to think that they always have to come into the fireside- if that makes sense. That is the individual- if you have got your own yard then do whatever. But its for those who don't have the luxury, you know, it is about know that you don't have to (do what others are doing / rug / feed/ stable). And for them, the important thing is to ask for the advice, the proper advice from your vet or people who have the proper credentials, not your friend from down the road. I the other thing, and I don't know if you have looked at our website, we do have things like how to actually body condition score and fat score your horse- trying to split it up into the basic three sections and then how to weight it with the tape measure… it's not to the last kilo but it gives people an idea of how much weight their animal has been putting on- or losing. Another thing is the rider -horse weight ratio. So many people are actually too heavy to ride their horses. Then they put the weight on so that it feels better for them to get on their horse. So then they are asking their horse to carry three times the weight that they should be. We have come across a few people who have said, well I have to feed it up because I weight…12st and it should only be carrying 10 stone, so I have made it put on weight! It's the opposite way, so that went wrong! So now we have got to try to make people aware than muscle weighs more than fat- so work it!! Everyone has lost sight of what the horse should look like.

AW: Is there anything else that you think would be important for us to include in the conversation?

WCM1 Not really, I have a motto which is KIS- keep, it simple. People overthink and they just, your horse is more resilient than you give it credit for so let it do its own thing sometimes… and your best friend is your vet- equine vet. Think about what animal you have got. It has not evolved to stand in the stable. In needs to move and search. If you've got a racehorse in race training, then fine- it will need the energy input. If you have got a happy hacker, it can still do the job with less input. So be realistic about how much work your horse is actually doing. That will help in this pandemic time, and it will also help horses to not have laminitis in November!

Interviewer: Ashley Ward (AW)

Interviewee: F2

Date and time: July 6th, 2020, 08:00.

Location: Aberdeenshire. Remote

Recording was difficult to hear in places on this interview: Notes were taken throughout- direct noted quotes will be used to supplement the audio.

AW: How has the pandemic altered your day to day routine?

F2: I have been extremely lucky that my day to day hasn't been impacted. This is because I work on a very strict 6-week cycle with all of my clients, meaning that I work for 4 weeks and am off for the remaining two. At the beginning I deferred my cycle to 8-weeks for shoeing and 10 weeks for trimming. That pretty much took me through the severe lock-down stages. I was surprised that more people didn't just take their shoes off really.

AW: How did you let your clients know that you had made this decision?

F2: I got all of their email addresses and sent out an email saying this is how I am going to be doing it. I was proactive in telling clients, this is how I want it to be. That included asking clients to maintain distancing, to tie up horses and to leave me to it.

AW: How did you decide on the changes you wanted to make to your practices?

F2: Collective information from various sources really, and just basic common sense and good hygiene. Social distancing- I employed based on the government guidance, and the Farriers Registration Council gave a really good traffic light system approach.

AW: Were your instructions largely adhered to by your clients?

F2: 99% of my clients were really good at putting everything in place. The horses were tied up and I could get on with things. I'm really lucky in that most of my clients keep their horses on their own land, so it was much easier to maintain distancing.

AW: What about the 1% who were not great?

F2: Well there was one bigger yard I went to that was just, "Business as usual", which I wasn't very happy about.

AW: Do you feel as though horse owners were given adequate information to make good judgement calls with regards to managing their farrier visits?

F2: Well, I think its mostly just common sense. I think the BHS gave some advice regarding the farrier.

AW: Do you think there have been any overall changes in the welfare or care of the animals you shoe?

F2: I think with the competitions being off, people stepped away from the stress of competition, and everyone is slightly more relaxed about looking after them. Just enjoying their horses from a leisure perspective.

AW: Would you say that the extra two weeks resulted in a significant difference in your client's horses?

F2: I was certainly surprised at how much extending the time between visits did in a couple of cases. One in particular, an ex-laminitic you could say, I was surprised at how much the growth had spread in that time. We were lucky because it was so dry at the beginning of lock-down we weren't seeing much hoof growth. Then obviously with the wet weather and sun we have seen more at the latter end.

AW: Have you seen an impact on the welfare of any of your client's horses?

F2: In the north east we are fortunate that most clients are higher up the (economic) ladder, very few were furloughed and generally they are better off- so most keep their horses at home on their own properties and have not had any difficulties with paying for farriery.

AW: How have you found enacting your biosecurity protocols?

F2: We had a seminar from a collaboration with the horse trust about biosecurity and things so that was useful. Largely it has all been fine. Some farriers have been shielding, but I think I realised that leaving my horses for any longer I would end up with a backlog, so you just have to get on with it as best you can.

AW: Have you had to take on clients whose farriers have been shielding?

F2: Well yes. I have been conscious not to steal clients, so I have made it clear, you know, that this was just a temporary measure. But it is a fairly small community of farriers here, so we have a WhatsApp group and I put a message on there saying – here's what I'm doing. It was mainly the clients who came to me saying, "Can you shoe my horse?", and my first question is always, "Who is your farrier?", because I know if they are shielding. Some then try to keep you on, and you have to say no.

AW: On the whole, would you say there has been an impact on there region in terms of laminitis management?

F2: 8-10 years ago, you would be seeing client's horses with those turned up toes, and you would say – this is becoming a bit of a welfare case. But you don't see that now. I don't know, maybe it's because I have more choice over who I take on now, where-as back then I would just take on anyone- and they would be more likely to have ponies that you would have concerns over their welfare. But not so much now, I haven't seen anything that has concerned me with regards to equine welfare or laminitis.

AW: What would our say have been the biggest obstacle to the equine industry during the pandemic?

F2: I think just that there needs to be more common sense. You know, some farriers have been very strict- you know, saying I will only see horses that will stand easily and if they don't, they're not getting done. I think if bigger governing bodies could have given information to horse owners, that would have even helpful. There was a lack of cohesive information. I think if there had been a clear authority that said, relax, here's what you are doing, then that would have been better. People seemed to take information from lots of different sources – like the discipline specific bodies were giving information to some, and no overall body made collaboration difficult. I think maybe vets could have stepped in more, but they have had their hands tied behind their backs and have been doing a good job given their situation.

Interviewer: Ashley Ward (AW)

Interviewee: HH2

Date and time: June 8th, 2020 14:00

Location: Aberdeenshire. Remote

AW: First of all, could you take me through your day to day routine before the pandemic, and how you used to deal with incorporated the horses into your routine?

HH2: Emmm, it hasn't changed to be honest; it is still the same. In the morning I check on them. They are outside, they basically live outside as much as possible. So, I go out in the morning and check on them, this time of year I don't feed them because they get plenty from the grass, they have access to. Then I go into work, I 'm still going to work at the moment. Then when I get back from work, I spend a little longer with the horses, picking out the fields, and that type of thing.

AW: Okay so, have your horses experienced laminitis before.

HH2: Yes, my Shetland pony gets it every year. This is the third year of it.

AW: Do you have any methods that you start to put into place to pre-empt that from happening?

HH2: Yeah, the last couple of years he was quite good- usually you start seeing him getting a little foot sore, so that's usually the first thing. This year it was later, but last year it was the middle of February which caught me completely of guard because it was a warmer winter and the grass had started to come through. Its always when the grass comes through that it bothers him. Once the grass is established, and it has grown when he is eating the longer grass roots rather than the shorter, that's when he starts getting sore. So generally, I keep an eye on him when he gets sore, and then I keep him inside for usually about 2 weeks. Then to be honest he usually lets me know; I leave the stable door open when I'm doing other stuff. Either he wants to leave, and he leaves – or he stays inside. I take that as a sign. Once he starts to want out, I take that as a sign, "Okay I'll try you back in the field and see how you get on". Otherwise he just won't leave his stall. He is a good little indicator himself. Then I just have to keep and eye on it. This year he was in, went back out, but then the weather turned cold and we had a frost snap- he got sore and had to come back in again for about another week. He then went back out. This year he wouldn't stay in the same as previous years, he started getting very stressed about being in. The others were calling, and he would start pacing a bit, so I ended up letting him back out because I decided that wasn't helping him much if he was stressed. Even, I watched him when he was outside- he will still spend a lot of time in the field shelter not eating grass- but at least there he can see the other two and can interact with them- I think that's what he is looking for. When he is inside the company is the radio and that's it, I'm afraid.

AW: What time of year was it that he had the first episode?

HH2: This year it was into April, because I was thinking you would have been coming out to do some tests and you would have caught him right at the time, he was just starting some soreness. Whereas last year it was February it started. The year before it was April into may- so it really must, I'm just guessing- it must just be depending on the winter and how quickly the grass comes through. They are eating it down pretty much by January- they are getting fed a lot more hay in the field- January, February, March, so they have eaten it down by then. I think it just starts to come through and you get the little shoots coming through- I think its them that seems to set him on. Because once the grass

is established, because I have a winter and a summer field, the grass will be longer so I find that he seems to cope with that a bit better- the longer grass he is obviously just cutting the top of it- rather than eating it down where obviously the sugars are further down the stalk. As I say, he will just suddenly change and then he is fine. The only thing I have managed to piece together seems to be the grass. When it is longer it doesn't seem to bother him.

AW: Do you usually get a vet to come out?

HH2: I just recognise the signs. On the first couple of days if he is really sore, I give him Danilon in his food, and that usually takes the edge off, but after that I've just left him. Last year I did get the vet out- they tested for EMS-which I thought was the weirdest test to do because he was inside and really sore, and they gave me this whole sugar thing I had to give him – and I'm like – I can tell you he is sore he is off his feet already- why do we have to put more sugar in his system. Then it came back negative for EMS and I was like, really? Then my farrier was out to trim not long after that and she had a vet over from the US that specialises in barefoot trimming and laminitis, and she said do you mind if she comes around- I said no that's fine. She was saying, I don't understand why the vets still do that test, because it doesn't' t actually tell you anything. It doesn't tell you what you want to know- it gives you back a reading that is clearly false because you can tell by looking at him, he is off his feet. It is happening every year; the chances are he does have underlying EMS.

AW: Have you needed to get the vets during covid?

HH2: No, I've been okay, I've managed with the Danilon and keeping him inside. The trimmer has come out every 5 weeks as we normally do- she says look- I'm going to keep coming to you because we can socially distance. She said, your ponies' feet are just going to take off if you don't keep on top of them. I think it would have been different probably because at the end of the 5 weeks they have grown, and you start to get a little bit of a shape- like to turned up toe look. You can see the outline starting to turn. So, I glad that she is able to keep coming and to keep doing that.

AW: That's brilliant. Was that a conversation at the beginning of lockdown?

HH2: No at the beginning of lockdown, she sends out information to all her clients to say that its lockdown and she couldn't come. But she separately texted me and said, you've got this guy and you're on a private yard, so I am happy enough to come out and do it. It would have taken a lot more work to try to get him back after lockdown was lifted and he would have been very uncomfortable too if they had just left his feet for three months.

AW: So, you don't feed your horses at the moment?

HH2: No not in the summer, they've got round enough bellies and they are not in competition. Its not like they are high performing, so they are just your typical hack around horses really. I've got feed, you know the horse nuts you can get, I usually have some of them in my pocket and I'll give them a couple each. So, they're getting something., but not anything big.

AW: Have you still been managing to hack?

HH2: I haven't gone off property- I've got bits round about us, but I haven't been doing nearly as much as I usually do. I decided I wasn't going to go out onto the road, just go with what we are supposed to be doing – staying a t home.

AW: Was that from guidance?

HH2: No that was just a personal decision- following government guidance for us I thought, I can do what I can. I think the horses are probably quite happy with, last month anyway, they weather was nice, and they just enjoyed lying out in the sunshine.

AW: Have you seen a difference in their weight or condition this year in comparison to last year?

HH2: Not really, the pony has been really good with his weight. Obviously, I can't ride him anyway, but his weight has stayed the same. The other two haven't been too bad, the last few weeks I was thinking they had giant bellies. When I look back, usually at the start of the season they get quite round. This winter was the best winter I've had for managing git. I tend to try to work the way nature s. So, they do put on a bit of a covering over the summer, but then they drop over the winter months. So, coming into spring, they're not skinny minnies but they have dropped weight. So, when they get access to the grass again, they don't blow up as badly as they used to. I used to have that probably after they had access to the grass you would then see the really overweight horse. The last few years I've done it through rugs. I've kept just a sheet on as long as I could until it got really cold in winter, then put on their thicker rugs. Then I took it off again and put back on a sheet to protect them if it was wet. That's the only thing I've done differently, and I noticed that they have been better with their weight. I used to be the type of person who would rug heavily over winter. They do have shelters in their fields so, I certainly have noticed that last winter, definitely thins winter, they certainly dropped the weight going into the spring a much better weight.

AW: In terms of moving muck heaps and pasture management, how have you been coping with that during lockdown.

HH2: My neighbour is a farmer, so I just have a big muck pile in the cart, and as I pick out the muck I through it on the cart. I then text him to let him know they its full and he comes and picks it up. They use it in their fields. So, there is no difference there.

AW: do you usually use any trainers that you haven't been able to have?

HH2: No, I don't

AW: Do you have any friends who you feel have been impacted?

HH2: Yeah, I have a colleague at work who has a trainer every 8-10 weeks from Holland, and I know she hasn't bene able to do that. Also, for a start she wasn't getting to the yard either. So, she was on a private yard which was a shame because her mare was expecting a foal. They did get the foal and she had to go in, but I think it is more restricted, there are times they are given when they can go to the yard which was a bit more restrictive. Before that she was able to fit in the horse round about her own work, but now she has to be there at whatever time she has been given- she just has to go to the

yard. You can see that inflexibility is a bit more tying. I wouldn't like to be told, you can be here between six and eight and that's your two-hour window- because you never know what could happen at work. You might get held up and end up arriving there for just half an hour and that's your slot, you have to get out again

AW: Do you think there has been equine specific guidance that you have noticed?

HH2: Uhm, I haven't noticed any, the latest BHS magazine is lying unopened on my table so they might have something. I come from a farming background and my mum is a vulnerable person, I was still going down once a week to her to save her from going to the supermarket. I have noticed going the cross country drive to get there- usually on a Sunday you would come across horses out hacking or you d see horse poo on their road, and I haven't seen much of that in the last 12 weeks, so I don't know that's just an observation.

AW: do you have any concerns for horse welfare?

HH2: I don't know how the livery yards are managing the horses, but on the assumption that they are still turning them out for the length of time they would normally do then that would be fine. But there is always a chance that if they reduce the number of staff on the yard as well to help with social distancing – there is the potential that horses might be locked up longer than they normally are. I was in Canada for ten years. One of my horses I had in Canada, we had an issue there because the winters are so bad there was sometimes- the longest stretch I ever had, was 10 days that my horse didn't get outside at all because the fields were like sheets of ice- due to the snow then the freezing. And they wouldn't let the owners turn the horses out for fear of them getting hurt and for the potential to get sued. Being north America there is always that concern. So that was the longest I ever had was 10 days that he didn't get out at all- he was in his stall other than me taking him to exercise him in the indoor arena. Reflecting on that, there is the potential that if the yards here have to cut down on staff, does that mean they have cut down on the amount of time they have turned the horses out for?

AW: Is there anything that could have been done differently with horses and horse management during the pandemic?

HH2: I'm not sure if there is. I was fortunate that the blacksmith came, but I have heard others that weren't going gout initially- so that has an impact especially on horses that are shod. There would have been more of an impact for them. I haven't used the vet for the horse, but I did have to use it for my dog. The experience there was we just had to explain what was going on, then send a photograph of what we had. He had a sort of funny growth on his anus, and I was concerned it was a cancerous tumour, and they didn't want us to come down, in was just a case of sending a photograph and we will look at it. They said in another couple of weeks if it gets worse comeback. It didn't really ease your mind per say. Whereas if it hadn't been the pandemic you would have just trotted to the vet and they would have looked at it. Instead they said we think it's this, but it could be cancer- already you're thinking about cancer but hold on another two weeks and see. You just think, if there wasn't the pandemic, your first visit would have eased your mind better- a photograph doesn't tell you much.

An equine dentist came out to me at the beginning as well, on the basis that I was on a private yard and she was seeing one client per day on a private yard. She told me a story of a client that she had, that the horse had problems with its teeth but all it really needed was good dentistry, but she said obviously that wasn't happening. The client ended up calling out a vet, and the vet came out and said- I think we are going to have to put your horse down. She said that horse didn't need to be put down.,

that was just the easy answer for the when they didn’t want to have to deal with a horse with an issue. She said a good bit of dentistry work would have solved that issue. I thought, I hope that’s not going to happen if you phone up saying my horse is colicking, and the knee jerk reaction is juts to put the horse down because we are not coming out. It just made me wonder. I haven’t bene in a situation like that, so I don’t know.

AW: Have you been impacted socially?

HH2: No. I haven’t had that issue at all.

END-----------------------------

Interview 2

HL2

**AW: Could you give me an overview of how the pandemic has impacted your day to day running of your business?**

HL2: Well financially it didn't impact the business at all, because the clients were all happy for us to continue looking after the horses. Negative impact financially would be the fact that we had 2 totally DIY ponies and 1 part DIY which we took over the care of. So extra staff hours. On a day to day, we completely changed the horse's regime, because they weren't getting any exercise, so instead of them being in from 3pm to 8 -9 am, we turned that around and had them out by 3pm and in by 8.30 in the morning. So, we could spend time with them and see them during the day, which is what the clients would normally do in the evenings. That gave them lots of walking around, so that when the clients were allowed back on the yard, we didn't have any riding related incidents.

**AW: So, you prioritised the welfare of the horse from a behavioural perspective to make sure the time your staff did spend with them was an interactive time.**

HL2: well I thin kit would have been terrible for them to be in their normal regime and not have any contact with their owners in the evenings, and not really any contact with the staff except from being led in and out and getting checked,. So, it seemed to me that was good for the horses, good for us, and ultimately safer for the clients. And I have to say it has worked so well. The horses are so happy, and now that the clients are back on the yard, they have all agreed that we will continue this regime in the summer months, because it seems to be working for the animals.

**AW: That's brilliant. One of the things people have highlighted is that places who have restricted people coming on to the yard have experienced issues with behaviour due to not getting the same handling. Di you expect that?**

HL2: Yes, I totally expected that. My background prior to dressage is western riding, and western you do an enormous amount of ground work, a lot of bonding, a lot of join up, so my own horses, which are a mixture of welsh cobs, warmbloods Spanish and Portuguese – I just treat them exactly the same as I would the quarter horses. Because the are horses. They like to know who the leader is and who the follower is.

**AW: And all of your clients' have been very receptive to the change?**

HL2: Yes, the change in regime. I would say that the first 6 weeks of lockdown I had a Friday night video call with 3 clients, and I would say -representative of the yard. And for the first 6 weeks they were- do what you need to do to keep the staff and yourself safe. Totally trust you to look after the horses. But on week 7 that had slipped to, why can't you open, other yards are open, some yards didn't close, can't you look at them and find a way through this. I realised that for 2 of the ladies, this was becoming a mental health issue. So, at that point I decided to get the clients' back, but on a timetable where they had little to no contact with the staff. Or with anyone except their riding buddy. Those partnership riding buddies stayed the same throughout the timetable period- which would be around 5-6 weeks.

We are now back to open access, but buckets of disinfectant and sanitiser is everywhere, still social distancing- protect yourself and protect everyone else.

**AW: Great, that sounds really sensible. The mental health aspect is very interesting and is quite important in these conversations.**

**HL2**: Yes, I HAD FOUR CLIETNS that I was seriously concerned about. Bear in mind I have known some of these ladies for decades, and I was very concerned about four of them. That made me accelerate the decision to allow them back on a timetable- against the wishes of one of my staff- but life has to be a balance.

**AW: Having experience in livery yards, I can see from a staff perspective how much easier life could be in some ways.**

**HL2**: Oh yes, I imagine it would be the same for teachers inf they had the children but not the parents.

**AW: Definitely, I can see where the staff's reluctance to get back to normal might come in. Do you think your staff would have happily stayed in the "no client" structure that they were in?**

HH3: Oh forever! Absolutely. I think it is a fine line to walk, because- yes, I am responsible for the health and safety of my staff, which includes safety from coronavirus, but also mental health. But I am also responsible for the safety and well being of the horses and the client. At the end of the day, as I explained in a staff meeting, without the clients – who could at any time come and pack up their horse and go elsewhere- without them there would be no income, and there would be no money to pay staff wages. So, reality has to kick in and you need to find a way through this.

**AW: The decisions that you made, were they guided by any specific governing body?**

**HL2**: Largely from my self, the BHS would be my governing body, but they really focus on riding schools and how to get people back into having riding lessons. The livery side is a very small part of what they do, which is an issue that people who are relatively new in post in the BHS, they are aware of and they are addressing. But really it was based on looking at our situation and saying-the clients need back on, I am the first aider, I am recovering from a broken heel so I can walk but I can't walk in the fields. So, I need to have 2 people here. If one of them has an accident the other can call me or get help. So, I let the liveries in out with staff hours for an hour and a half slot. 2 people in a stable block that we were not using for anything else. So, it was completely separate from the staff, separate toilet, stables 12 feet apart and each client was assigned which stable they could use. So, I had one stable for those who I know have autoimmune disorders and need to be very careful, and the other was used by those who I know to be perfectly healthy. That fortunately worked in terms of the horses that were turned out together and the people who were compatible riding together- that actually worked out really well. The last person who rode tended to by a co-owner of a horse with me, so I was there with her anyway, and if not- the last person leaving had to text me to say I am leaving, and I am safe. So yeah. It just took a little bit of organisation, but it did work very well.

AW: It sounds as though biosecurity was at the forefront of your set up. It sounds as though your facilities could easily accommodate things like strangles.

HL2: Yeah, we are very strangles aware. We quarantine in field and stables. I have never had it and don't want it, but I have had an outbreak of ring worm and gosh that is a devil to get rid of. You find yourself disinfecting fence posts! Also, one of my grooms is the groom for RDA and another one has an animal sanctuary of his own. So, we have to be very careful that they don't bring anything here, and they don't take anything from here to their own yards.

AW: So, you're quite used to handling these sorts of thing on a day to day basis even out with the pandemic?

HL2: I just think that horse owners are the ideal group of people to deal with a human pandemic, because we all know, wash your hands, don't let your horses share buckets, don't let them touch noses and it is just the same with human. Don't crowd someone else's space and attend to your personal hygiene. Hopefully we will come out of this pandemic with people realising they have to wash their hands occasionally.

AW: On your yard, has laminitis been a concern?

HL2: We have one that has EMS, so we need to be very careful. He is PRE, we have a Lusitano who needs to be very careful, I have a welsh cob that would be very prone as it grows fat just looking at grass, and there is a warmblood cross welsh cob who is also inclined to obesity. So, the PRE, welsh cob and the welsh cob x all wear muzzles. We are creep feeding them because we have so much grass. Everyone does this year; the grass is insane. So, every day the fences get moved 2 feet and that's it. The fatties go out with a muzzle so they get some of the grass, but not as much as they would want. Then the muzzle comes off during the day and they get haylage.

AW: Is that something that you would do every year anyway?

HL2: Pretty much, and I have to say I was very impressed yesterday. Two of my more experienced staff came to me and said that one of my horses, the PRE, the left feet, front and back are warm, but her right feet are cold. Now, to me, normally you would expect laminitis in the front or the back- not on one side. It is that attention to detail that I just think my staff are great. To notice that. Most yards don't even pick out feet, so they wouldn't be aware. When we are picking out feet, we are looking under the horse, feeling the legs- it is a proper check. Whereas most places are just changing the rugs and chucking it in the field. Attention to detail I think is very important. Horses are put on this world to commit suicide, slowly and expensively. Our job is to try to slow the process down.

AW: It sounds as though your concerns for laminitis must be mitigated quite a lot by the fact that your staff will notice these slight changes.

HL2: Yes, I have a young warmblood and 6 weeks ago, one front foot wasn't hot, we couldn't see anything, she's not shod, and we didn't know- but at that point vets weren't coming out. Without taking

her somewhere for x-rays there was nothing to be done. So, we just checked her twice a day every day, put her on hay, and kept her off the grass for 2 weeks. She is now completely sound. Was it laminitis? I don't know, but if in doubt, treat it as laminitis. I think that is what makes this yard different. I am in and out of a lot of different yards and have friends on different yards, and they just look chaotic to me. No-one seems to know what anyone else is doing, and there are just whiteboards with lists of what to do. Some yards didn't do anything different. I am led to believe by friends who livery there, that there were no buckets of disinfectant, no sanitizer, the toilet is revolting at the best of times, and clients were allowed to come and go with total freedom. I think that is bonkers. Whether you believe in the pandemic or not, you have to at least protect your staff. So, I don't quite understand the mindset behind that. But they have got away with it, so I'm sure they would see it as a successful way to deal with it.

AW: Yes, it has been highlighted a couple of times that people would like to see sanitisation check points where disinfectant and hand sanitiser is available, and you mentioned that you have that in place but I don't believe many other places have that.

HL2: Yes, that is the feedback I have been having from friends and clients, and it costs nothing to have buckets of disinfectant. All we did was put buckets of disinfectant beside the two lockdown stables that the clients were using. They were only allowed to use a single door to get in and out of the arena- only staff could touch the main doors. We moved all of the tack to a tack room next to the lockdown stable block, so there was disinfectant outside that, outside the toilet. But what does it cost you, a pound? I did get feedback from others from other yards who were astonished that we were providing disinfectant and disinfecting stations. They said at their yard, even if they were on a timetable, there was nothing provided, they were told to bring their own hand sanitiser. Which is okay until the day someone forgets it.

AW: Do you take the stance that it is the responsibility of the yard owner/ manager to put these things in place?

HL2: Totally, this is part of your health and safety assessment. What on my yard could harm a member of staff, a client or a visitor? If you have a door that is about to fall off its hinges, you don't allow anyone near it until you get it fixed. So, if you have a virus, and you can't see it- anyone who is carrying it- it is an invisible threat to health, therefore washing hands and staying apart and disinfecting works. They all has to sign a list of rules and a lockdown timetable, and they all had to sign that they read and accepted and would abide by the rules Which included disinfecting everything in the stable that they were about to touch or had touched. So, when you arrive and when you leave. We took the horses trough and left them in the stable for them. They then had to disinfect the stable, door handles and tack. Everyone did it, I was there quite a lot in the first couple of weeks until everyone settled in and they were absolutely brilliant.

AW: That's great, it sounds as though you took the responsibility of keeping everyone safe and that that allowed the owners to follow a protocol more effectively.

HL2: I think, I have a couple of ladies who have significant autoimmune issues, and it gave them peace of mind that they were safe to come and see their horses. One lady only came to see her horse in the field, but she was taking her bucket of disinfectant and her sponge round to disinfect the gate. I just love scientists- I have so many working and liverying here. You tell them this is the way I want you to do it and they say great! Give them a protocol and they are happy!

**AW: It sounds very positive, I could keep asking more about what you have done as it sounds as though we could learn a lot from the way that you have balanced staying under the guidelines but also keeping liveries happy and managing to prioritise their mental health within all of that.**

HL2: I think I haven't quite stuck to the guidelines. If you look to the BHS there has been all of this talk about- you can't use indoor schools, which is ridiculous. They are not a sports-hall, they are well ventilated. I took the decision that I would much rather clients were getting back on horses that had 7 weeks off, in the indoor school rather than the outdoor. 1 week in they were allowed to use the indoor school only, then after that they could use the outdoor school, and then we made a hacking track outside the 5 acres field. But we didn't allow that in the first week in case the horses found it a bit exciting. Now we allow it, but social distancing and disinfecting are routine.

**AW: So, the only thing you really moved away from the guidelines regarding exercise?**

HL2: From my perspective the exercise was an important priority for the horses- you don't make money out of this, I do it because I love horse. So, you have the issue that you need to look after the horse's mental health, because if you don't, sooner or later, either a member of staff or an owner, is going to have a very nasty accident. And that is just the way it is. So you have to put the horse in the forefront and say, okay, how can we manage this so that these horses can stay in their normal herd, get handled, they are in a routine where they are not cooped up in a stable for 18 hours a day. Once they start being ridden, lets ride them 2 at a time, because they have become a little institutionalised. So, I allowed two people to lunge at the same time- I don't allow lunging whilst someone is riding because I think it is an accident waiting to happen. But I suggested that for the first week, you might want to lunge, at least before you get on. And I felt that was safest in the indoor. So, a little bit of divergence there, but I regard my yard as an independent state, and as though its not illegal we can do what we want.

**AW: That has been fantastic, is there anything else that you would say is important for our conversation with regards to equine welfare in the area during the pandemic?**

HL2; I think it's just, the amount of grass sickness, colic's and laminitic was horrific- and that is partly due to the weather we have been having, but I think largely due to the fact that a lot of clients weren't seeing their horses. They weren't seeing their horses, they weren't monitoring, or they were in such a tizzy to do their horse in the allotted time span, that things were being missed. One thing I did do- every Tuesday we pulled out every single horse, we pulled out all of the horses and took pictures from every angle. And that was our condition score day. So our clients could see their horses and know how their condition was, and also so that we had a record to look back and compare, and to be able to say- oh this horse is getting tubby we need to put a muzzle on. But of course, that takes a bit of time and not every yard is able to do that. The owners needed the WhatsApp group, I created it in the first week of

lockdown because emails weren’t hitting it for them. They wanted something on their phone, a cute photo or a little video or the condition scores. It made everyone feel that they were still part of the yard. And that is what it should be. Everybody should be happy on a yard and enjoy their time. Particularly the horses.

END-----------------------------

Interview 2

V2

**AW: With the limited face-to-face interactions and consultations, are you finding yourself performing more telephone consultations?**

V2: Personally, I like to do that, because I been doing that for a long time anyway. Just because for many years I have had WhatsApp, so my phone is filled with stuff from my work. I find it so useful that I just can't do without it. I think it actually creates a better, more relaxed, relationship between vets and clients. I think there is a general fear that, "I'm not going to take my animal to the vet because that is going to cost me £35, so I have been encouraging WhatsApping for years. So, I haven't noticed any change over the last 10 weeks with COVID, other than just doing what I was doing before. I will get to see animals, especially production animals, that I have never seen before. Because in production animals it is a very different world, a lot to do with economics, and you don't get to see the animal unless you have to, unless the farmer thinks there is going to be a cost benefit. So I have found that just by having more chats with the production animal farmer, then I will get more chance to visit, or to encourage the animal to be brought in to the clinic. So for sure, I will personally continue to do this. The counter to this is that the RCVS code of conduct is that we should, wherever possible, we should be seeing animals before prescribing medicines. This has been an interesting period because, through necessity, we haven't been able to do that. Especially in the early days of COVID, so we were given a derogation such that, you are not allowed to see the animals, so do your best to prescribe over the phone.

**AW: That is interesting, would you say that with regards to laminitis, the management has changed in line with this?**

V2: Absolutely, yeah. It is, obviously it would be nice to physically visit, but I like to encourage the long term bond between me and clients, and would like them to feel relaxed about calling. You know, if you go back and rebandage a horse for 6 months, you work up a big bill. So from that point of view, I try to limit visits as much as possible- but I continue communicating and providing advice through the phone.

**AW: Have there been any cases when you have visited an animal, but rehabilitation would have been more intense than restrictions would have allowed?**

V2: Well, small animal really has been lock door. That has been a very different scenario to production animal. And the equine situation has been somewhere in between. The assumption is that the equine can be tied up to a stable and the owner can distance themselves while the vet treats the animal. So, there is a spectrum of interaction which is very polarised between production animal (and the other groups). Because with production animal, there has been a general feeling of, "We have to keep the production animals healthy and productive." So, we have been carrying on as normal, with very few limitations. Small animals are at the other end of the spectrum and equines are in between.

**AW: Have you noticed any alteration in the horses behaviour that you treat?**

V2; I guess, if you are visiting big livery units then that would be a big issue. But here in the north east we tend to visit individual units, single units where you have one owner, with one or three horses. This is quite different to other practices visiting stables with 30 horses, and for those situations I do think that (difficult behaviour) for sure will have been an issue. When I used to work with, visiting these big units, it was a huge social event. There was a posse of people there, almost too many people. If you have a horse with a cut leg, everyone wanted to be there watching so that was a difficulty which I presume the liveries have sorted out in COVID by keeping liveries away as much as they can. So, I don't really have any experience of that, but I can get the feeling of that.

AW: That is interesting, and I suppose the two types of set up are quite incomparible in many ways.

V2: Yes, and with the doctors it has been very clear what their path has been, but with us, especially with myself working with all species, I have to think,"Right, what scenario am I getting in to here". With all the best will in the world, I have been pretty close to some people through the nature of my work through the lockdown period. What we did to try to mitigate that during the lockdown period was, when the client phones in, the receptionist would pose the question, "Does the household have any contact with the virus as far as you are aware". That is recorded, so that if we had any issue down the line then we could back track it. But we are in a fortunate position that in this corner, we have been relatively unscathed by COVID.

AW: And the clients that you have been to see, have they been quite pro-active in employing good practices?

V2: Yeah, the equine owners are fine. The biggest challenge we have had are the farmers. The average age is 60, so there is a graph of risk awareness, and once you get into the over 60 farmers they just don't want to be 2m away! I will be reversing and they just approach.

AW; Do you think that the nature of farmers and animal owners, was that considered in the guidance?

V2: I don't know because I didn't really get it. If we were getting given guidance I wasn't really studying every bit of guidance I was getting. I was just following the simple biosecurity measures that we are always aware of, I have no doubt that the RC and BVA have been setting guidance, and also my employer who is a corporate employer, they would give out instructions every day.

AW: You mentioned that you have been following fairly established biosecurity measures that were always a consideration. Do you feel that having experience of previous epidemics in the animal field has given you more confidence in dealing with this pandemic?

V2: Well, with foot and mouth, we weren't impacted up here. The foot and mouth episodes were very much limited to southern regions, so I don't have any personal experience of foot and mouth. But there have been other issues where, I suppose veterinary surgeons are well equipped for an emerging disease scenario. We are getting it all the time in our Veterinary Records magazine, so there is lots of stuff in there about emerging diseases. As part of our continued professional development is often attending speakers who have knowledge of specific condition, so we touch on that and we are pretty

well prepared. The foot and mouth thing, again, I am sure that will be handled differently again. Because the bottom line is that it isn't a zoonotic disease. But to get back to COVID, we are all pretty smart now that we are 3 months into it, but my personal take on it is that the care homes seem to be behind the curve. I find that disappointing because we don't have a lot of care homes, and the ones we do have been handled poorly. What we have been doing for a long time with regards to the intensive piggery industry, and the hen and chicken industry, where we have strict biosecurity- and the farmers have been doing that for decades. I feel that we missed a trick there with care homes, in terms of straight forward biosecurity- with having a simple shower in shower out system- which didn't happen as far as I understand. I think that should be handled better if there was another pandemic situation. With no vaccines about now I think care homes could still put that into place.

The piggeries that I have spent time at were very high health status, and it was very straight forward. You have a changing room, and a shower, outside clothes don't go beyond the shower. There has been a lot of work done in the animal world with regard to transmissible disease, particularly in avarian and porcines. I spent few years with a pig farmer in the north east, and I can recall a lot of research going into, with swabs being taken from nostrils. So, I was involved with various projects looking at pig diseases in my respiratory system and it was quite interesting because I was the pig farmers personal vet. So, I had to observe very strict biosecurity, because we had one piggery of particularly high health, where he was producing guilds and bores of particularly high health status, and he was pushing them down the pyramid to another piggery, which was of a lesser health status. So, I had to arrange my visits so that I was going down the pyramid at all times, otherwise I was observing 72 hours free from any pigs before I could go back into the piggery of the higher status.

We will probably get better at handling COVID. It's not going to go away quick. It obviously has, in other parts of the world, shown its capability of going quiet and then coming out again. I think we will have the same issue here, particularly when it gets to winter-time. So, it will be interesting to see how places like the care homes carry on. I wonder as well how the bigger care homes, like ARI will cope with it. I've never been keen on having visitors coming into hospitals- that just seems so wrong. I mean, yes, when there should be exceptions to the rule, but I would keep visitors right out. We find, even in our clinics, I can remember a dog, which is now no longer, but it had MRSA, and it just happened to be that I was dealing with this dog, and the owners were really switched on with MRSA because the couple were both switched on as they both had hospital experiences. When I phoned them up to say that the urine sample had MRSA they were horrified and they kind of went into a lock down situation there and then. That is an example of where, it is everywhere! And I'm sure that going back to the early days of the NHS, the biosecurity would have been far, far better than what it is now. In 1946-47 when they were starting out, and the nursing system for human health was phenomenal. Compared to now, it is a hospital, and yes, there are always serious situations, but there is also a feeling of no one there reducing, or trying to speak about the basics of, "It would work far better- yes your relative is in care, but we will care for your relative and we will be able to give more care to you relative if you stay away. And just communicate with your mobiles". It is very old hat- maybe I am just running around the country oblivious to them having it all sorted.

I know nothing about it other than my wifes mother who was nursing in the 50's , and she brings out these stories of people being in isolation for a year and ahlf. So I am intrigued by that! My wifes mother herself, at around 6 months of age, she was in isolation for over a year in Aberdeen, in a hospital. Nd you just don't hear about that.

So Perspex, for example- there might have to be more production of Perspex with a sanitorium scenario- rather than just having hospitals that we have at the moment.

AW: I think that is interesting- we have really been focussing on the impact of the pandemic on equine welfare, but really you are speaking about the core of the issue- and that is probably where the most work can be done.

V2: It is a fascinating subject.

Interview 2

WCM2

**AW: Could you tell me about your day to day during the pandemic?**

WCM2: Well, on a day to day basis it is kind of the same so to speak. I think the biggest impact we would have had here would be with all the restrictions that with the nature of the types of horses we have we had to be even more cautious to make sure no one got unnecessarily hurt. So, I think maybe the rehabilitation of the horses has been impacted in that we have done a little bit less with horses that we would have maybe done a little bit more with under normal circumstances. Like I say, just in case of unnecessary injury. I think the different types of horses we have- the ridden horses we chose to give them a break. So, I think the impact of that would be that after the break we then had to bring the horses back into work and they had to stay here longer than they would have done. But I think really for me that would be the biggest thing. But afterwards we seem to have caught up, because we had two or three months when we weren't really able to rehome horses, so taking a positive spin on it, it gave us a bit of time to think about how we do things and why we do them. So, taking the opportunity for some self-evaluation and I think, obviously now we have played catch up, I think it has been positive in a sense. From the evaluation point of view, and the only negative part of it was that we weren't able to rehome the horses, and we just had to be very, very careful.

**AW: Did you see in increase in admissions to your centre at all?**

WCM2: No, during the actual lockdown time, we had the odd horse, but when the restrictions got relaxed, then we saw a rise in the amount of horses coming here. But still, I don't think we have seen the full impact of it yet. I think it is almost a little bit, the calm before the storm. We have had an intake of horses since the restrictions have been lifted, but I wouldn't say a huge amount.

**AW: Right, okay. At the beginning of lockdown, how did you manage things like social distancing that were introduced?**

WCM2: We imposed little bits and bobs, stricter social distancing. We didn't stop handling the horses because I thought it would be detrimental to their (development). Because, you know, if we stopped handling some of the horses and they became, what I would call feral; if those horses then had an accident or we needed to treat them for anything during that period, then actually it would have become more of a welfare issue. So, we didn't continue with anything that we hadn't already done with them. What I would say is that we maintained their skill set while maintaining social distancing and the health and safety of the staff at the same time.

**AW: And in laminitis cases on your yard- have you seen any change in those?**

WCM2: I don't want to tempt fate, but we haven't actually had any. We did turn all of our horses out, but I still brought horses in on a daily basis to let them stand in off the grass. So we still sort of, watched the calorie intake of those horses that were laminitis candidates, but luckily we haven't had any. And again, we just brought the horses in all day and then they went out at night, so they were just on the grass for a shorter period of time. And obviously the guidelines were to turn the horse out, rough the horse off, which we did to a point. We didn't ride. But there is, with the riding, you know,

some horses, like my own horse, there is no way I could have stopped riding it because once the muscle goes his stifles slip- and then it becomes a welfare issue. So I think it was about risk assessing it, I didn't jump it, I didn't hack it around the farm. I rode it inside. I know the horse really well. So yeah, I think it is just, it was about risk assessing things for the safety of the horses and the staff. And laminitis was a big thing for me, I didn't want any of the horses getting laminitis, so that is why I had what I call a fat field. It was a field with not very much grass on it, and all of the horses that looked a bit rounder than I would like, they basically lived out for a short period at night, came in all day, and got turned out again later on. So, yeah, we just managed it.

AW: In terms of the guidance that was given to horse owners to stop riding, did you think it was adequate?

WCM2: I think, the types of horses we have at the centre, the cobbier type horses, you know you can let them down, you can stop riding them. They were never fit to start with, you know they were only fit for the job that they were doing. But the sort of, more competition type horses, my horse is a warmblood- quite sharp- not naughty, you know? I ride her every day, but had I not continued to do something with that horse- because mentally it needs stimulated, she would have maybe hurt herself in the field. She is only 5 and has weak stifles. So, if I don't maintain the work the muscle development goes, and the stifles start to slip. So, yeah, things like that- people need to risk assess what they are doing. There were many days I would have liked to take her for a hack around the farm, but the horses were out, and they could have galloped over, she would then get silly- so I chose not to do that. I chose to ride her inside, where I know she is going to be fine and I would be as safe as I can be.

AW: Have you noticed a separation into two groups – one which the centres have allowed riding and one which are not allowed to ride?

WCM2: Not really a conflict, but I do a lot of teaching and in the local area- some horses have been in work because they have not had restrictions on the yard- or the only restriction they have had are time slots where they have a limited amount of time to go to the yard and do their own horse, but then other people I have been to teach- their horses have had nearly 3 months off as their yard owners have said- no riding and that's all there is to it. To be honest I think that is a very brave thing to say to people. If, like I talked about with my horse, if you caused their horses to be…. You know, it could have been detrimental to the horses. So actually, I think for the yard owners to do that, I think it's a really bold and brave thing to do.

AW: Do you have any overarching concerns for equine welfare in your area?

WCM2: Ehm, no nothing in particular really. When I have been teaching I have maybe made the odd comment that a clients horse might be a little bit rounder than it should be. But not really any more so than I would see throughout the year. You know, most horses get a little bit fatter in the summer when they get access to the grass. Some horses get fatter in the winter when their owners bring them in and feed them. So no, I haven't really seen anything like that I don't think.

AW: So, for you, is laminitis any more of a concern than it would be normally?

WCM2: Yeah, I personally haven't seen it. But that is something going back to people saying, you can't exercise your hoses – I think that is a very bold thing to say, because there will be a lot of people who will have taken that as gospel, and literally stopped doing anything with their horses. Whereas- I don't mean to sound big headed- educated people like me will have taken the advice but not to the letter. So we did it to maintain the safety of the staff the horses and everybody else, but we took measured risks to also maintain the welfare of the horses at the same time.

**AW: That is interesting- so the guidance has been largely interpreted individually?**

WCM2: Yeah, because (horse owners who follow the guidance to the letter) are probably not experienced enough to make those calls. So, if you say no riding, you know a lot of people are going to be quite impressionable on that and are literally stop riding their horse.  But that could cause a whole host of problems for the horse, and the person I suppose. Because they can become a bit naughty if they are not mentally stimulated, but then the guidelines could have been a bit better because there are things that you can do with your horses without having to sit on top of them. You know, there are things that mentally stimulate the horse without physically putting yourself at too much risk.

**AW: Did you come across any advice like that?**

WCM2: Eh, I don't … I think people were just advised not to. To be honest I think some of the advice was a bit airy fairy, but then I suppose it had to be airy fairy because if you impose restrictions on people then it's them who have got to carry the can if it goes wrong. I think the guidelines could have covered more aspects.

**AW: In terms of your centre, have you found it easy enough to manage your staff as you go along?**

WCM2: Yeah it has been quite easy. I don't usually have any more than about 5 staff, so I haven't- I know some of the other farms have put their staff on a rota basis, but I haven't here. We have been very careful- when having breakfast, lunch and breaks a lot of girls have been very good and have gone and sat in their car, when the weather has been good we have sat outside, because otherwise, what they used to do is they would have a room where they would all sit around a table. So they have stopped doing that. If there were horses that needed two of you to be quite close together, then they were the horses that we didn't do a great deal with, unless you could manage. Even having a lunge line so that you were at least a meter away from each other. Even the farrer- the farrier came and we just tied the horses up for him and left him to it. So we were quite lucky, and we were lucky with the vet as well. They came and did things that needed to be done. Obviously routine stuff was knocked on the head, but stuff that needed to be done was. And they were quite good, because they brought another vet, so they could socially distance themselves from the staff. So we had to have a couple of horses castrated because they were getting a little bit full of themselves. So actually two vets came which was excellent. Because if they hadn't, our staff then would have been very close. So that was quite good. So we still could get stuff done but it was still very safe for everybody.

**AW: And have you had any cases or situations that could have been easily dealt with under normal circumstances, but that required more complex actions due to the restrictions?**

WCM2: No, luckily, we haven't. We have had to make choices where the horse was not safe enough to continue with- but it was going to be not safe enough whether it was the pandemic or not. So yeah, luckily, we haven't really had to make decisions like that. Because all of the horses we have here- lots were feral, but before the lockdown we had got to grips with them. So even though they were a bit nervy and not fully there yet, they were able to be handled and were manageable. So, we do try to get on top of them as quickly as we can- because as I said if you don't and you have to treat the horse for something then it can be a lot, lot worse.

**AW: Is there anything else that you think is important to include in the conversation?**

WCM2: I don't think so. I already mentioned the fact that I like to think of it as not a negative thing that has happened. I think kit has given everybody a lot of time to learn. It has given us time to do things on the farm that we never get the chance to do. You know we painted the arena a different colour, and it gave everybody a sense of pride to improve the place- even though there are no visitors and we are closed, I think for us it is nice to be able to take a bit more pride in it. Usually we wouldn't get the chance to do that, there is no way we would be able to paint the arena, or the office or the tack room – there is no way we would have had time to do that. Because we used to be open three days a week, and we would be rehabilitating the horses on all of the other days, people would take holidays and days off- you would just never get things like that done. You know, it has been positive. And I have actually got cross with people who have moaned about it, because actually what is a hardship? Having a little bit more time to yourself? Spending time with your family? Its not like you've been called up to war is it?

**AW: Do you think that could be something that could be taken forward- maybe taking some time to catch up with tasks?**

WCM2: Yeah, I think so. Before I came here, I worked in a college, so when the college was closed in June, we actually got that time every year. Whereas here it is pretty full on all year round. When actually if there was time to get on top of things, it would actually help everybody. We use to fininsh at the end of June, and once all the paperwork was done, the stables were all painted, the horses had a rest. It gave everyone a chance to think about the coming year and how we are going to do things differently. What was good about this year, what wasn't so good, what wouldn't we do again. And I think we have actually had a chance now to do that here at the farm, and it has been good.

**AW: Do you have anything in particular that you have reflected upon that you will take forward?**

WCM2: I think just taking the time to enjoy the journey with the horses. You know, just taking that time to enjoy it. To enjoy the journey as opposed to it becoming like a bit of a production line. Obviously no one could go out for lunch and no one could go out for tea, so a positive from that is you save some money!

Interviewer: Ashley Ward (AW)

Interviewee: HH3

Date and time: July 6th, 3030 09:30

Location: Aberdeenshire. Remote

**AW: So, I see that you keep your horses at home, would you mind outlining the set up that you have and how you keep them?**

HH3: Yeah, at the moment I've just got one highland pony, se is in a paddock that is probably around ¼ of an acre, about a 30x40 paddock. She is out during the day and in at night. When out during the day she has a hay net of soaked hay, which has been soaked overnight, for a full 14 hours Then when she comes in at night she gets a feed of hifi light, top spec antilam, lamatox supplement, and a pump of cod-liver oil and another soaked hay net to keep her going for the night. She's on a straw bed so she does eat a lot of straw- but to be honest I would rather she ate that than the grass, so it keeps her happy.

**AW: That is great, so it sounds as though you have a lot of preventative measures in place to prevent laminitis, is that the focus of how you manage her?**

HH3: Yeah, to be honest last February 3019, I had to stop riding because I was pregnant, and about a month later she was diagnosed with laminitis. So, I had from then until August she was on box rest, she was on thyroxine to get weight off and also Danilon obviously to help with the pain. The hard part was that she didn't really show her pain, so it was hard to know how bad she was. We had a few stages of x-rays taken. She did have rotation and sinkage, so we obviously required remedial farriery. It took us a long time to get her sound again. I would say she was sound by August, but then around two months later she had a relapse. Then she was in the box again for another 3 months, and then since Christmas 3019, we have been building her back up and she is now in the best health and fitness she has ever been in. So, I am really pleased with her now. So that's why I have all these things in place. She also gets ridden 4 times a week with a variety of schooling hacking and lunging.

**AW: It must be in your awareness that when you stopped riding, she developed laminitis for the first time.**

HH3: I will tell you something very embarrassing. I have had horses for 30 odd years, and I thought I know how she should look and what she should look like. When she developed laminitis, I knew she was fat, but I really didn't see how fat she was. She was actually 740 kgs and I weighed her the other day and she is now down to 550 kg. So, she has lost nearly 300 kg which is absolutely massive. So that's her journey into where she is now, and I wouldn't have her any other way now. I think native ponies are worse because they hold it in so many different places. She didn't look big in the belly area, but she was holding it in pockets everywhere, she had a big crest and it was on her bum.

**AW: Has this all been difficult to keep up during the pandemic?**

HH3: To be honest it has actually been easier. Both me and my husband have been working from home, so it has been easier. If there is a day if she looks big or whatever, you can just bring her in. That routine is easier as we aren't' having to rush away in the morning to get to work. We have time during the day, and I've got time to go out and ride if I want to. I think it would be different if she was at livery.

AW: That sounds ideal. Have you still been able to access all the specialist feeding and hay that you have been using?

HH3: it has, we have a farm as well, so we have a good relationship with our local feed suppliers at harbo. We haven't had any problems getting hold of anything and we can just ring up and collect it instead of going into the shop. If for whatever reason they don't have something in they just order it and its there in a couple of says. The only thing I have to order in is the lamatox supplement. But I haven't had any problems getting that either. But we have been really lucky.

AW: Have you been able to manage farrier and vet visits?

HH3: Yes, I am in a good position with the farrier. I just leave her in the stable in the morning and he just comes and sees her, then I just send the money via online banking- he just keeps in touch through the phone. The vets have all been really good, they have told us tat they have been struggling as they have had to furlough staff. But when we have needed them here it has been absolutely fine.

AW: That's great. Have you been getting out riding as much as usual?

HH3: Yeah again, we are in a very fortunate position where we are very rural, so I have just carried on riding as normal. I have an arena at home, so I am in a very fortunate position with that to keep her ticking over. We have got woodlands around here and you can go riding and never see anyone, se we are very fortunate.

AW: A few people have been saying that there have been more people out and about- have you noticed that?

HH3: The road that we live on is rural, and there are nicer areas to access that our road, so people really haven't been around at all.

Aw: Do you have any specific routine for keeping on top of your pasture?

HH3: I just poo pick it every couple of days, I have three sheep on it at the moment and she is wormed every 13 weeks. The rest of the field has sheep on, and she is in an electric fenced paddock within. That's all I really do, if we need to get rid of docks, we just strim them away. It has taken us a long time to get here, but we have created the ideal for how we work and how we want to manage the animals.

AW: Do you normally compete or travel at this time of year?

HH3: I normally do showing and dressage during the summer, not as much during the winter, obviously we haven't been doing it this year, but to be honest I have just been getting her fit, so it hasn't been an issue. I would have normally taken her to the beach more, and we have just started to do that again now. We have missed that, but it hasn't impacted on her levels of fitness.

AW: Some people seem to be more relaxed without the competition pressures., have you found it more pleasant riding for fitness?

HH3: I really enjoy having something to aim for, but I haven't missed it that much. I haven't' wished I was showing, but there have been a few shows online and got my husband to video and send it in. That has kept us going and it something to do.

AW: In the overall native pony community in Aberdeenshire- do you have any concerns that relate to the pandemic?

HH3: I do have huge concerns over obesity in the showing world, but not directly associated with the pandemic because everyone has done their own thing, nothing has been organised that shouldn't have been organised.

AW: We worried that people might have turned horses away in the field at the beginning of the pandemic and then we would see a spike in laminitic sand obesity, have you seen that in your area?

HH3: No not at all, two of the community that I know have native ponies and they have just carried on as normal. One has a pony who had laminitis and his owner has kept on top of hm, although he is very overweight. The other has a small paddock and has been managing that so no concerns at all.

AW: With regards to getting information and guidance about managing horses, have you received any information on how to approach things that might be difficult during the pandemic?

HH3: I haven't received anything directly, but I haven't been looking for it and I think If you wanted to find out more you could go on Facebook. I had seen a few articles about it there. There was someone who put out guidance, some competition body, but I can't remember who now, they gave out some guidance.

AW: Do you think that direct guidance would have been useful to receive? Maybe a checklist of dos and don'ts?

HH3: I think so , the only thing is, we can say that- so if we say the bhs should have sent out something to all horse owners, but how are they going to do that, because if you're not signed up as a member your not going to get it. If they do input something on Facebook, you see it on your newsfeed.

AW: Someone else I was speaking to said they would have liked their vets to issue guidance, do you think that would have been appropriate?

HH3: I'm not sure that is really their role. I guess as a horse owner your actions and what you do with your horse is your responsibility, it is like your doctors telling your if you can or can't go outside. I think it is up to everyone and their personal circumstances and what they think is right or wrong. That's up to them though, I think it is really down to the individual.

AW: I heard a story of some conflict between riders and non-riders, have you faced any conflict or disagreement between other horse owners?

HH3: Not at all nothing like that. Everyone I live near are all farmers and they have been out doing their work as normal – they have had to. I think we all have a mutual understanding that animals like horses need working to keep them going. I can imagine in that situation it would be very difficult. I think if I was in that situation I would just say, look this animal can't be left in the field to get fat, she will die, kind of thing. It must be really difficult.

AW: Those are all my questions, but is there anything else that you would add that you think is important to include?

HH3: I don't think so. Whether it is right or wrong I am not sure- I have carried on as normal, apart from not taking her away from home. I think it has been important to keep them going because I can guarantee with 3 months off work, she would have ended up needing to be kept inside and that wouldn't have been good mentally or physically. For me the trigger with her laminitis was the fact that I stopped riding her. If I had continued riding and had got some weight off doing that, I don't think she would have got laminitis in the first place. Its like people, if people have Type 3 diabetes then doctors with tell people to get outside, eat less and exercise more. If I felt that she was unsafe of that I had a high risk of falling off, I wouldn't. I didn't do anything dangerous – she is a very safe and reliable pony, so I just carried on as I deemed acceptable. I think for my sanity as well as hers. It is definitely a big part of our mental health.

END-----------------------------------------------

Interview 3

HL3

**AW: Can you tell me a bit about your set up and where you keep your horses?**

HL3: The horses are in a field in the middle of town kind of, in a forest. It is a really nice field there. They used to be my mum's horses, so they used to be somewhere up on the hills, but now they are just in there. I've got a Shetland pony, and she is 9, and an Arab cross – my mum got him when we were really young, so he has been in the family. He is still going; I'm looking after him and he was lame for a while but now I have been getting back on him and he has been really good actually. He loves going out on adventures. It is a field that's owned by a farmer, but its in a public forest area about 5 miles from where I live. It is really nice for riding around there.

**AW: So how do you incorporate the horses into your routine on a day to day basis?**

HL3: So, the Shetland is the one in danger of laminitis, and she also has sweet itch as well. She is the one that I have to do a lot more day to day care with. Usually I go every morning and I feed them and put neem oil on her for the sweet itch and just do various things with fly rugs and masks and things like that. Then I'll either take pixie out for a run or the other out for a ride. There are two other horses there that I ride also.

**AW: Do you have a field shelter or stables to use?**

HL3; Yeah, we have a field shelter.

**AW: So, if Pixie was to develop laminitis, do you have somewhere to keep her off the grass?**

HL3: Well when spring hits, this is the first year of me looking after the horses myself, so I wasn't aware but another owner pointed out pink rings around her hooves and I thought, I had better make sure she isn't eating too much grass. So, I put her in a long thin field on her own within the field, and that helped loads, the pink rings have gone now. Actually, the others have eaten down the grass in other areas of the field.

**AW: Has she developed laminitis to the point where she is lame?**

HL3: No, she hasn't, it was just the pink rings on the feet that we noticed as an early sign.

**AW: With the care of the two, has covid impacted your ability to look after them the way you like to?**

HL3: In a way it has, because there have been a few ladies who come and help, so having to juggle that so that we can all socially distance and things has been a bit of work. Also, actually, its almost busier at the field because so many people have been going for walks there, so navigating that has

been difficult as well. So, I have just been going to the horses really early to avoid that because it gets so busy there.

AW: Have you noticed people interacting with them over the gate?

HL3: Yeah that has been an issue. We have put up signs, but people definitely ignore that. Some people kept coming and feeding them carrots and apples, so I had to say to them, if you are going to give them something you have to give them a horse treat that isn't a sugary snack!

Aw: So, the biggest thing you have noticed is people being around more. Have you had any difficulties with managing the paddocks?

HL3: Yeah, we pick up the poos every day. Actually, another thing was that people were doing lots of gardening, so we found poo piles had completely disappeared- people had taken them. We didn't need to get it collected so that was fine. The farmer who owns the field usually uses it for his other work.

AW: Has your work- life balance been impacted during this time?

HL3: I have definitely had more time to look after the horses, because not having so much of a structure in my day, I ended up just going up to the horses and being there for like four hours.

AW: Has that time been spent riding?

HL3: Not necessarily more riding, I was just hanging out there, brushing them, pulling off ticks. All the things that you never really had time to do.

AW; Have you been able to access horse shops?

HL3: Yeah, I have ended up going online more. But I do prefer to support my local places rather than buying online. I have noticed our local feedstore have been posting a lot on Facebook and things, so I think that probably helps them.

AW: And have you been able to access your farrier as normal?

HL3: Actually, that has been difficult. At first, she cancelled an appointment and said I won't come out unless it is really necessary which kind of left their feet a little too long. Then she said she was going to drop clients and stop doing so much. They are barefoot, so we had to find another barefoot trimmer. We did find another one. It was a bit difficult, but luckily, we found another lady.

Aw: And your vets as well, have you needed them?

HL3: No, I haven't actually needed them, so I haven't faced that problem.

AW: In terms of sharing space with the others there, has that been tricky to manage that?

HL3: Well we all have certain days where we know someone is picking up the poos, and with me going early in the morning, it meant that we weren't all there at the same time.

AW: Did you implement any disinfecting protocols?

HL3: No, I just made sure to wash my hands and distance. But we didn't really talk about it at all. We all look after them in the way that we will pick out all of the poos and if one of them needs a fly mask we will do it.

AW: Was it the government guidance that led you the most?

HL3: Yeah. I was riding one day, and this lady started shouting at me and said that I wasn't allowed to be riding. She had her horse at livery, and they weren't allowing people to ride their horses, and she said, you have to look at the BHS, it is illegal to be riding your horse right now. So, I went and looked at the BHS and it said it is kind of up to you whether you want to ride your horse or not. With the horses being at risk of laminitis, I didn't want to stop riding. So, I felt like I had a reason to be riding. I think if he was the kind of horse that, you know, on him I'm just going for a plod around the forest, and its not very far to fall. So, I did consider it, but I decided that it was best for both of us to carry on. So, I was a bit upset, but then I looked at the BHS and I was like, she's wrong! But thinking about it she was probably just upset because she couldn't ride her horse, so I can see it from her point of view.

AW: Did you have any other problems with people when out riding?

HL3; No that's the only incident so far.

AW: Obviously not everyone follows social media or the BHS, can you think of any groups who would have been in a good place to give universal horse related guidance?

HL3: I suppose the main place people would look to would be the BHS but not everyone does. There are lots of different groups on Facebook, but I don't remember anyone saying much about it, about what the actual guidelines are. I think it would be good if there was something that people could look to for guidance.

AW: Do you think that horse owners in the region have been impacted by this pandemic in a way that might have a lasting effect?

HL3: Well, I don't know. Probably not for me but I can imagine for people who wanted to make money from it, like riding schools, or people who were taking part in or running events, I imagine they will be very affected by it. Probably in the future too it will have a knock-on effect of horses not having so much training this year, that would have a knock-on effect for next year.

AW: Looking at laminitis, do you have any worries for horses in the local region?

HL3: I haven't seen any horses that I would worry about. All of the people I see around have sectioned off their fields and things like that. The one thing that I think would be really interesting is, I think there is an app that you can pay for that tells you about possible spikes in the sugar in the grass. That would be able to advise you on at-risk times. I think it would be so good, because at certain points I was definitely being too strict on keeping pixie in her tiny pasture and only letting her out for an hour of friendship time with the others. I just didn't know if it was safe to let her out with the others.

AW: We have covered everything that I would like to ask, is there anything else that you think we should include in the pandemic?

HL3: Ehm, I think it was interesting to think about how horses that usually would have been getting a lot of exercise ended up not getting so much. I was really worried about it because I had a horse which died from laminitis-she had Cushing's disease. I just really wanted to make sure that Pixie didn't get laminitis or get too fat. I guess you don't often know the extent that it can go to.

END--------------------------------------------

Interviewer: AW

Interviewee: V3

10TH July 2020: 1000

Recording was poor quality and so direct quotes in the form of notes were used to ensure correctness of transcription.

**AW:** Could you please start by giving an overview of the way the pandemic has impacted your practice and the way you work?

**V3:** Yep, sure. Well I would say that the biggest thing is that we have had four vets furloughed. We are a mixed practice, but it was just the pure SA vets who were furloughed. The workload has just increased due to this, so maybe normally I would see more equine and farm, whereas now I am having to fit in more of the small animal stuff too which can just be a bit challenging. Although for the first month we saw no equine cases at all. These were all put off. In the clinic we have just been engaging in all of the social distancing practices, so we have no clients coming into the clinic, and we are just seeing emergencies.  It has been about 10 weeks that we have been doing this and it's fine- it's just extremely busy. Now working predominantly in a telemedicine capacity is pretty normal, but it is difficult. I think handling prescriptions making sure that you are giving the right thing and that the owner is actually going to administer it can be hard. The pressures to re-start routine vaccinations is also, ehm, significant. We just can't keep up with the work load, and we have had to start re-doing them as the clients are just not co-operating. The number of calls we have about them is just overwhelming. I have spoken to some others who have said that their practices are still not doing them, and another that has opened a second practice just for vaccinations to handle the workload.

There are some as well who would phone and over exaggerate the issue because they knew that it would get them seen. The horse owning clients weren't as bad, but there were calls where people would state certain "buzzwords" just to get their animals seen, even when it was just for something routine. Horse owners were not as bad. The farm and equine were no where near as bad as the small animal. I think, on the whole, it was really the vets decision to see the animal based on whether the owner was happy for them to do that. It was hard to always know whether to see them or not. Farm animal is just as normal- it hasn't really changed at all I think it is harder to engage something like we have in SA in those environments.

**AW:** Do you feel as though the guidance issued addressed those types of scenarios?

**V3:** I do , I think it changed quickly and randomly, so it was hard to keep track of exactly what is allowed and what's not. Horse owners especially didn't seem to have huge amounts of guidelines to help them, but then it was easier to maintain social distancing and things like that in those outdoor scenarios.

**AW**: Do you feel as though your approach to practicing- relying more on telemedicine, is something that is useful?

**V3:** No , I am not good with the phone calls- I find it much harder to assess a situation. Obviously, you can send videos and get pictures to supplement the descriptions, but I think the main thing is

that there is a lot more work to that and not enough time in the day. Days have just been so busy. I think there is more room for misdiagnosis, and that's something I worry about, but it's so busy. In the phone calls as well, they will start talking about something completely different, going off topic, then the information that you actually want to know, like dates and how long something has been like that for, if they are on any medication, that is the information they don't know and you can spend a long time on the phone in a situation like that.

**AW:** Do you feel that your clients have been adequately informed on how to handle obtaining veterinary treatment for something they are worried about?

**V3**: I don't know. It's hard to say, I mean maybe they have this information- they can certainly find it if they look for it online. I don't know if it is up to the vet practice to give that information.

**AW**: And, in terms of equine cases specifically, is there anything that concerns you in particular, more-so given the pandemic?

**V3**: No I don't think so. I mean, last year was an absolute crop year for laminitis. I don't have an exact number for that or this year, but I would say that is something that concerns me generally most of the time, I am not sure that it has changed because of the pandemic specifically.

**AW:** Are there any preventative strategies that you feel have been complicated by the pandemic?

**V3:** I mean I suppose with regards to exercising horses, like, my own horse, it is not overly clear on which way to go, whether to ride or not. I mean, you could say to do more lunging or inhand work- but it hasn't really been clear. Certainly , in my case there has been no hacking or jumping, for safety reasons, but I think you can't really put something in place (in terms of guidelines) that suits everyone. There are always going to be some who do not agree. It's hard to get the right pitch with guidance, you wouldn't want to say- more lunging, and then have a laminitic horse in pain, then you have a welfare issue. So , it is hard to get the right pitch.

**AW**: How do you feel things are progressing, do you have any ideas that would minimise your concerns over laminitis and equine welfare in general?

I think there are a couple of ongoing things, like owners being furloughed- you do worry about horses' care being dropped or shoeing or trimming being missed. I think that concise, clear guidance would make a difference, like, something that people could follow. For vet practices, there was a bit of heads butting, with some practices offering things that others weren't and registering new clients. That was something we didn't do, once we restarted vaccines, we did only those that had been registered with the practice at the start of the pandemic. So we didn't take on any one new. Some practices were taking on new clients just for vaccinations, and we had some calls from clients who were registered with others but just wanted us to vaccinate their horse or their dog or whatever.

END---------------------------------------------------

Interview 3 WCM 3

**WCM3**: Again, that means there is less attention being payed on the animals.

**AW: Do you think that has meant that things have been missed with the animals that usually wouldn't have been missed under normal circumstances?**

**WCM3**: Ehm, I don't think so. There was one thing, I was supposed to collect a urine sample from one of the mares. That was just before lockdown and so there was nothing being sent off. But finding the time to hang around and collect the pee (was hard), because we have got limited volunteers.

**AW: Yes, I completely understand! That is great that you could get the vaccinations done before lockdown. You said that most of our horses were okay for the farrier, what about the others?**

**WCM3**: Well, we have got two huge French cobs, so they are only about 16.3 hh, but they are absolutely massive. They are so big that you can't get your hand around their pastern, so they need to be sedated because they are so big. Only because the farrier can't get a proper grip. Some we had to extend our initial farriery visits, because we have 6 big horses, 5 ponies and 2 donkeys. So, he usually comes every 6-8 weeks to do the horses and the ponies, then he comes a couple of weeks later to do the two big cobs, the donkeys and the Shetland stallion. We took in a stallion who was 19, so he is stabled next to the donkeys. He is now 27, but he is really good, and we keep him separately from the mares. So that was the only thing that was done, it was extended to 10 weeks. But the farrier is happy with that. There are no welfare issues. So, they have had their feet done every 10 weeks at the moment.

**AW: Do you have laminitics in your care at the moment?**

**WCM3**: We have a Shetland pony who has EMS, so he has to be monitored quite closely. But our ponies don't go onto grass in the summertime. They just stay on the year-round arena. Obviously, a little grass comes through but never much, and he comes in at night. We also have a 20-year-old Shetland pony who we took in recently, she had been unintendedly neglected by hr owners. So, we took her on, she is the one we were trying to get the urine sample from. I think it is probably Cushing's, but we will see what happens with her. She is pot-bellied but very thin, and that is very like a Cushing pony we have had before. She is on Cush-care and molasses free hi-fi. She has a problem with her teeth as well, she kind of sooks on the hay. She comes in at night and gets a big bucket of molasses free hi-fi. We are trying to avoid the laminitis risk for her.

**AW: I assume that this is more expensive than normal food?**

**WCM3**: Yes, especially when we are supplementing with hay. The hi-fi is about £15 a bag. It is very scary, with donations reducing.

**AW: Do you think there is a reason for donations reducing?**

**WCM3**: Well, when lockdown first happened, people were very generous. There was something in the mail every day from our wish list. Now, I don't know If people think the lockdown is over, and they are reverting back to normal. Or, it may be because they are thinking the furlough is going to stop, or they are out of a job and there is just no money available. I fully understand that, it just makes it difficult when you are in our situation relying on donations.

**AW: In terms of the overall impact of the pandemic on equine welfare, are you worried that you will see a specific type of horse being referred to you?**

**WCM3**: I think there will be a lot of companion horses looking for homes or being put to sleep. You know when the oil companies laid off loads of people a few years ago, there were lots of companion horses, unridable horses and older horses being put to sleep. At that point we had to have an animal taken away by Grey's and they were saying they were taking away double the number of horses they normally did. Because people just can't afford to keep them, and I think that's what will happen now. Once people are out of jobs and the economy has crashed, I think there will be a lot of unusable horses that people will get rid of.

**AW: Will you be in a position to take on more animals if you are increasingly approached?**

**WCM3**: No, we are full. We always like to have a stable for the horse, because they all come in at night in the winter, and we are struggling as it is. And financially we need to take care of the ones we have got. And we do only have 17 acres, so we are really at full capacity. I maybe could squeeze in a pony, but I certainly couldn't take a bigger one in. All the other rescues will be the same.

**AW: How do you think that the guidance has been sufficient for people to make calls on their horse's care?**

**WCM3**: Well I am just, kind of, working along with the vets and the farriers, but my partner actually works at Hogholm livery yard. The yard was closed to clients for quite a while, so it was just the staff looking after the horses. They followed the BHS guidelines, so people are back now, but initially they weren't allowed to come on the yard, the horses were brought down onto the block. But now I think they can ride and things, and over that period of time of lockdown the farrier stopped coming and it was only vet emergencies. So, I don't know if horses had shoes on over that time, so that will have had a knock-on effect on farriers- they will then have just been doing trimming rather than shoeing. But I do personally think there was no real proper information about riding your horses. So I know some people were riding their horses on the roads during lockdown, which I personally think is quite silly because if you have an accident you are putting a lot of people under pressure who are already under a lot of pressure. So, I think the guidelines about riding were not clear, they should have been more, "do this, or do this", but it could be read into what you wanted to read into it.

**AW: Do you think that riding was a good idea then?**

**WCM3**: I don't know, I don't think riding out on the roads or riding in public would be a good idea. If you are riding in your sand school and you know your horse, maybe? I don't have time anymore, but I probably wouldn't have ridden my horse during lockdown. But if you were in your sand school or on your property fine, but I don't think it was sensible to be riding out in public. Then if you are hurt, or your horse is hurt, you have to get the vet or the ambulance out. You are then taking up bed space for a potential COVID patients.

**AW: Is there anything else you think you could add to the conversation?**

**WCM3**: I mean obviously if there was another, bigger, wave- how will that affect things with vet treatment or farrier treatment. I mean, when the lockdown happened, the first thing we did was buy a pallet of pig feed and a panel of chicken feed. Because the others I knew we could easily feed the others by adapting their feed. But the pigs and the chickens would be the most difficult to adapt their food. So that is what we did, we bought a whole pallet which did us for while you maybe weren't wanting to go out and buy things or to do too much shopping. So, we did a lot of stockpiling at the beginning. So, if things get worse and we had a really big lockdown, then it is a big concern that food stuffs might be hard to get.

Interviewer: Ashley Ward (AW)

Interviewee: F3

Date and time: June 30th, 1100

Location: Aberdeenshire. Remote

**AW: Could you tell me, in general, how your day to day has been altered since the pandemic started?**

FF2: Well in general, I have been busier than ever. There have been two factors: 1 I have been covering for other farriers who have been off shielding themselves, ; and 2 people have been riding their horses more with being off work, so they have more time to ride which is really going against the grain when the recommendation is limiting the amount of exercise and going out the house as little as possible. But anyway, I have been busier than ever really.

**AW: That is really interesting, would you say that they are doing the same type of work with their horses, or have they changed that?**

F2: Well, some that have never been ridden have been getting fit and getting shoes on, and they are riding them now. Other than that, the type of work is pretty much the same as they were doing before, just more of it.

**AW: How are you finding the yards that you visit, is everything set up in a way that you can carry out your work safely?**

F2: Yeah, definitely. I think people have been pretty sensible. Those at livery yards have generally been booking in a time for everyone at the yard to get done at once, to limit the number of people on and off the yard. Going through bank transfer rather than cash payment. People who are in their own private areas, they are either just leaving their horse tied up so that I'm not needing to come into contact with them, and carrying out a bank transfer again, rather than face to face contact. It has been a handy thing to have.

**AW: That's great, is that something that you will continue to do after the pandemic?**

F2: I can do, yes, it is great for having the availability to transfer money. It is handier for the customers, the only difficult thing is if you are in a rea with poor signal it can be a bit difficult to get the payment to come through, but I think it is a great idea otherwise.

**AW: Have you been happy to visit yards throughout the pandemic or have you shielded at any point?**

F2: I didn't shield but I did try to put as much work off as I could. The ones that have got really good feet that don't need done as regularly as they were getting done, I tried to push them back a wee bit at the start, thinking that this lockdown was going to be shorter term. As the months started going by, you start realising that it wasn't just going to be a quick fix, so from that point on I started to put them back to their original cycles.

AW: When you started seeing them again, have you noticed any difference in the quality of their feet?

F2: Fairly similar, the weather has had a big part to do with it, with a lot of wet and dry spells. Otherwise most horses have coped pretty well with it.

AW: In terms of the differences between those who keep their horses at home and those on livery yards- do you have a preference between those two scenarios?

F2: Not massively, everyone is being sensible enough about it, as at the livery yard you have people booking in time spots so there are less people on the yard at the best of times. And most places have a well thought out area to be able to shoe in in a safe environment. Whether it is at an individual stable, or at the back of a tie up area- just somewhere away from the rest of the group, that has worked out well. Obviously at the private yards you generally only have a single person, so you have massively reduced the number of people you have come into contact with.

AW: Have you seen any overall impact on equine welfare in terms of their shoeing or any other concerns?

F2: There have been a few people who have been struggling I think with losing jobs and lack of funds that have been trying to maybe stretch them more than what they should be but I wouldn't say it have been a welfare case exactly, just some who have been in slightly poorer condition. I think that will all bounce back once things come back to semi-normality.

AW: Have you been seeing many laminitis cases across the time of the pandemic?

F2: A few, but not massive in numbers, it has been semi-controlled. Just the typical ones that are already predisposed to getting it, maybe with other medical conditions. Nothing scarily high or anything like that.

AW: Are you finding that people are able to manage them the same way they usually do?

F2: Well I think people are more understanding of the condition, especially with the veterinary research becoming more readily available for the customer to read. Also, people are much more clued up on the situation, especially with the drugs like prascend and metformin to treat the underlying condition, these things are massively improving welfare on the whole.

AW: It sounds as though you are optimistic that people are trying to do their best.

F2: I think generally there are. I think everyone tries to do their best. But there are factors can limit anyone's decision whether it be available time, work, they might not be able to reduce the grazing – that's just something we can't really control. If they are working a lot and are not on a livery yard, then its out of their hands. Unless they get someone else to do it. Then there are funds as well- for something like going on medication or getting regular farrier appointments- vet check-ups- everything has limiting factors. To what extent they can contribute- I feel that my customers do try their best.

AW: Do you find yourself giving advice to your clients, in terms of hoof management, as well as other aspects of management to people with laminitic horses?

F2: Its difficult as you can say about their feet- but at that point it is too late. Looking at their general welfare – weight, condition, body score or whatever- that's a difficult one as people get offended by what you say about their horse. Its difficult to word it, and I try to word it in a way like, your horse is on the big side, maybe try and reduce the amount of feeding. I never say starve them, but maybe soak hay.

AW: Are people quite receptive to that type of advice?

F2: I would say most listen, there are a few typical ones that will never see their pony being on a restricted diet- they always have them with treats and what not- and that's they way they have been and always will be. But I would say most of the people do listen very well, and when they start seeing maybe scare mongering, on Facebook with some horse that has had it, it maybe gives them a shock. So yeah, I think most things are going in the right direction really.

AW: We were worried about increased obesity and laminitis cases during the pandemic, do you think that has happened?

F2: There are two categories really, the people who are now furloughed and not working and they have had so much more time for their horse. They are riding more and doing more with them because they have nothing else to do, They can't go out and shop, they can't go out and social gather, so they are riding more. And then you've got the other category where they are struggling to pay for things so they are turning horses out in fields and leaving them really. But one the whole I would say most people are doing more with their horses.

AW: Do you think that is to the horses benefit?

F2: Some, yeah. Some yes and some no. Most increasing exercise and reducing intake is probably promoting health. I think we need to look at that as a whole as a country. I think there are a lot of grossly overweight horses, and I think it helps with a lot of the older horses too, getting them out and oosening them up . I would say on the whoel it helps.

AW: And with the advice that you were given from your governing bodies, was this effective?

F2; At the start yeah, we were given a traffic light system and we were advised to put them into categories. So a laminitic horse needs their horse don e every 6 weeks so make them your priority. But your cobby type that are barefoot-goo feet, they could maybe be pushed to 10 weeks or so. Obviously they advised us to take bank transfers more than anything else. Limit human interaction. I myself, have tried wearing gloves and using hand sanitiser, I sanitise my tools at the end of the day- all just to try to limit the amount of potential spread. I think if everyone tries to play within the rules then it should all sort itself out in time.

AW: Do you think the traffic light system has helped to avoid instances of welfare cases?

F2: That is a difficult one to answer. The time that this has gone on for has been unforeseen. I think everyone expected it to last for less time. The traffic light system was introduced at the start with the thinking that this would be over after a short time, so it worked well at the start. But,you push all these horses back, and they all need done at a certain point. Then all the farriers become very busy all at once. And again, some of these horses have been pushed back further than initially intended. It has its goods and bads. I think a lot of people in this area stuck to it as much as possible, and a few people stopped working for a bit. But you can only leave it for so long before you need to intervene again.

AW: So there wasn't really an update to renew the rules midway as we have drifted on.

F2: That's it, the basics they drummed into us was stick to bank transfers, hand sanitizer, reduce social interaction. Those are the three main things that I stick to, and I think most people have.

AW: Do you think that horse owners have been given equally helpful guidance?

F2: I think most people have been sticking to what is on the news more than anything. But I would say they have just been sticking to the general rules of the country.

AW: Some have suggested that there should have been a more integrated approach so that the whole industry was provided with guidance, taking into account the different roles. Do you think this would have been more helpful for farriers?

F2: Well yeah, for horses than need held for shoeing or trimming, it is difficult because where do you draw the line. Some horses that need held- I have asked them just to stand on the opposite side of the horse – just to distance ourselves with the horse in between us. But yeah, we have all been given as much guidance as we could expect really. They have given us all of the warning flags, areas we should try to avoid- it has then been put in our hands and the customers hands to make the informed decision on what is best to do. So maybe the ones that are more awkward to do, the best course has been to take the shoes off them and try to prolong their cycle- so we are not seeing them as regularly. But yeah.

AW: Is there anything that you could take from this that you have learned from?

F2: I would just say, if this exact thing was to crop up again- we are all lot more informed about how things happen now, so we would be a lot more sensible with addressing shoeing and horses,-it would be sensible to limit human interaction again. So even try to avoid meeting the customer where possible- have horses tied up and ready for us so that we don't need to put head collars on or touch anything. We should come in, horse is tied up, shoe the horse, text to say I'm leaving and finding bank transfer sent and job done. But again, everyone is an individual and you need to treat them as such. They aren't robots and you need to try to adapt to each individual situation.

AW: It sounds as though it potentially could be a very streamlined process, and the risk can be almost completely mitigated if prepared for.

F2: The other thing I could advise would be that if customers had hand washing facilities, even just a washing station, then that would be helpful. If you have hand sanitiser then that is a bonus, but at one stage there was a struggle to get stuff like that. But if you could sanitise all of your tools and your hands then that would make a massive difference.

The only other thing I could recommend is that if you are at a livery yard, try to get as many horses done in the same day. Then you're not in and out multiple times. Try to get yards done in days, rather than single horses at each yard, so you are limiting your spread area.

**AW: Would that be a case of the owners controlling scheduling or maybe the livery yard owner?**

F2: Either or really, who ever is responsible enough and who is willing to take charge of the situation.

**AW: How has your ability to take care of your own needs?**

F2: Well, most days I just bring my own stuff with me. You do sometimes stop, but everyone is trying to limit the amount of times they are stopping or having a cup of coffee at a yard. It has been trying to limit anything that you shouldn't be touching or interacting with you try to limit that as much as possible. Saying no to a cup of tea on a yard is difficult, but it's what you've got to do sometimes.

AW: In terms of job satisfaction, have you been happy working throughout this time, or has it had an impact on the way you feel about the work you do?

F2: No I have been happy to work in general, because, 1) I don't know anyone who has had it or who has been in contact with it – I have been asking all of my clients if they have been in contact with it before I arrive. If anyone had been, I would just put them off for a few weeks. But no, I haven't known anyone to be in contact with COVID-19, and so that gives you much more confidence in doing the work. And 2, most customers have been sensible enough to provide me with a safe working area, so on the whole it has been relatively straightforward. There has been the odd ball that has been thrown in but 90% of the work has been pretty normal.

AW; Can you describe an example of a difficult situation that you have found yourself in?

F2: It has only been the situation where a horse needs held, or young horses. For instances a horse that is a bit awkward to shoe, that needs a lick, or reassurance from the owner- the two-metre rule is difficult to hold. Especially if you are doing a Shetland pony, you can't get that rule enforced. So, at that point you need to employ face coverings, gloves, and them standing at the other side of the horse, and maybe face in the opposite direction as well. That is the only situation that I can see that has caused any hassle.

**AW: Asking clients if they have been in contact with the virus sounds like a good and systematic approach to making sure you aren't compromising yourself.**

F2: Yeah, just asking if they have any symptoms, if they know anyone who has, if they have been away out anywhere – have they visited others. You're trying to paint a picture in your head of the scenario before you arrive.

AW: Many people have said that the mental health of their clients has been impacted, is this something that you have experienced?

F2: I would say there have been people who have been open with me and told me that their mental health has been affected. 1 due to the lack of social aspect on these big yards who have been strict on their rules and giving time slots on the yard. Usually people like to go up, see their horse, have a wee coffee morning. Now its just basic welfare maintenance so I think people are missing out on the social interaction. I think it has made a massive difference to their lifestyle, so I would say yes it has made a difference.

AW: Is there anything else that you think is important to add to the conversation?

F2: No, I think we have covered all bases really. On the whole I feel that we see customers more regularly than the vets. A vet may only see a horse for boosters if that, and we are seeing them every 6-8 weeks. Whether it is dietary, or health and wellbeing, we are seeing more of that horse and the customers are more open to telling us stuff.

Interviewer: Ashley Ward (AW)

Interviewee: F4

Date and time: 13/06/2020. 14:00

Location: Aberdeenshire. Remote

AW: Would you outline your day-to-day approach to your work now during the pandemic?

F4: Yeah, it has really just involved going to yards, having the horses tied up, people keeping their social distance, probably having a few more horses that are getting sedated because they are youngsters, and they are better off if someone is holding them-but with social distancing you can't so you just get them sedated, or we are taking the shoes off and they are getting turned away until things improve.

AW: Do you have a lot of clients were happy to sedate to enable you to work, or was that something that you recommended?

F4: It was a mutual agreement that if they wanted to carry on, the best way to do it was to just get them sedated so that we could do the social distancing.

AW: Have those conversations been quite easy to have and fairly positive?

F4: Yeah, I've only had a couple who decided that they weren't going to be doing that much with them so they would just take the shoes off rather than carry on shoeing them. So we just got the shoes off and trimmed them up. Then instead of going 8 weeks they are maybe going 12. So that has stretched out a bit longer.

AW: Have you seen a detrimental impact on their feet from lengthening the intervals between shoeing?

F4: Not really, the weather has been good for their feet not growing as much as they normally would. So we are not getting the overgrown feet you would expect to see at 12 weeks.

AW: Are your clients largely keeping their horses in work?

F4: I think the first month / 6 weeks a lot of people just stopped. After that they got them back into work. A lot of their horses weren't in work and have been quite awkward to deal with- and the owners found that as well. So that is the reason why they brought them back into work.

AW: Did you notice a change in their behaviour?

F4: Yes, there were quite a few which almost became feral again, just because they weren't getting the same handling. That was a battle with them.

AW: Intersting, so we obviously had to balance up safety for us with the welfare of the horses, but it was important for these horses to be worked so that we could work around them?

F4: Yes, absolutely. It was quite interesting, just how they changed. They have been in work long enough that you would think 6 weeks off would have been fine. But I think the owners weren't handling them so much. Certainly a lot of livery yards were banning liveries for a month- 6 weeks anyway, so they weren't getting any handling pother than the people on the yards.

Aw: Has anyone mentioned their horses changing their way of going since having time off?

F4: I think for a lot of them the rest was quite good for them. There are so many things on all year round now, people just keep going. I think having 4-6 weeks break has made a difference to them. A few subtle lameness's because the vets couldn't get out- they have actually gone away because the horse has got a break. I am fortunate in that I have been at it of 35 years. When I first started, you had maybe 6-8 months when there were competitions going on, and then they got 4 months off. Because there were no indoor or outdoor schools, so there was no reason to ride them. They might hack them out at the weekend but that is it. If there were any niggles, they got 4 months of to recover and they were fine again.

AW: Have you seen anything, overall, within your client base, have you had any welfare concerns during the pandemic?

F4: There are strange cases of laminitis coming up – horses that aren't overweight, they have been in a little bit of work. Just the way the weather has been we have had a few getting laminitis, and quite bad cases of it as well?

AW: DO you think that owners have struggled to handle that?

F4: Yes, they have put restricted grazing and muzzles and things on them but it hasn't made much difference. With the dry spell, to wet and warm, to cold and wet, I think the grass growth has just been unpredictable. These were ponies that were living out 24/7 so they should have been able to adjust a lot better. I did one, took it out the stable, thought – you're not looking too comfortable, shod it, and it hobbled back into its stable. I thought. Omg, that's it, it has laminitis. But it came out just looking a bit stiff. But ½ an hour of standing getting shod just tipped it over the edge. That was quite bizzare. It was a horse. We were both quite surprised because it was in work and it was quite fit looking. Again, it was just that weird weather.

AW: Have you been called to trim any laminitic cases during the pandemic where the vet has been involved and you have needed to go an get on top of?

F4: There have been a couple where the vets have been to see them, and they have decided that they needed to get something done. A lot of them were using barefoot trimmers, and they weren't allowed to come back out because they weren't deemed as hoof care professionals. Whereas, as farriers, we were getting out on welfare grounds. And a lot of the barefoot trimmers travel quite a distance to get to them as well, which I think restricted their ability to get to them too.

I think that is why we got more calls to go and do them.

Aw: Have you had to fill in for other local farriers who are maybe shielding?

F4: Yeah, farriers who got letters from the government to shield- I have taken on quite a lot of their work.

AW: Was that something you were quite happy to do?

F4: Absolutely, we are a small community and we all try to help each other . We have been fortunate as we have a new farrier in the area who has taken on quite a lot of clients to help others out.

AW: That's great, it seems as though farriers have the best working relationships within the groups.

F4: Yeah, years ago we never used to get on with each other but now, we are all quite young, we have all come through competitions and apprenticeships and we all get on really well with each other now. Even the older ones have come around to being civil. It is quite interesting that horse owners think that we don't speak, and we don't get on with each other. At social events it's quite good to go along, we all get together and chat and stuff. The equestrian awards two years ago, the nominees all had to go up to the dance floor bit and then they read it out, and we were the only group of people that whenever we were called up we all went and shook each other's hands. We were all just happy to be nominated for it, without bitching to each other about anything. Its nice to know that your clients appreciate you and its nice to share that with the others.

AW: In terms of the horses that you have been seeing, have you been witness to any worrying cases that you would refer to a vet, or would think of as a welfare case?

F4: No, I think, personally, all my work is pre-booked, so 6 weeks today I know where I am going to be, because that is just how my diary works. But I have ones that were every four weeks because of shoeing needs, they need to get shod every four weeks because they go lame or their shoes fall off or whatever. So, on those welfare grounds, I have managed to keep going as normal. It would take a little bit longer to get that far. Some of them, they weren't that bad, but they stand a lot better if there was someone at their head. So instead of taking 45 minutes it was taking you the hour to do it. But you just had to get on with it.

AW: Are your clients back to holding the horse as normal now?

F4: A lot of them are- fidgety youngsters they are holding them now. Generally, they are speaking to my bum anyway- it's not face to face- so you know, we are not really that close- although we are maybe a metre apart we aren't actually in close contact. I have one customer who is completely over the top, who comes out with wellies, boiler suit, face masks, glasses, hair net, riding hat on. You think, there is no need for all that. But then I was speakingto another farrier who is insisting that people wear that if they want to come out and hold the horse. If they haven't got face masks then he will provide them with them. I was disinfecting my tools after every stop aswell, my tools, apron, everything was getting disinfected. As a precaution. I just do it at the end of the day now. Because everything was getting drenched in Jay's fluid, and I was getting a bit too used to the smell of it!

AW: How have you felt the traffic light system to work with?

F4: Yeah, the clients understood aswell, the trims getting done every 6-8 weeks were green, so we pushed them another 4-6 weeks, which would then take them almost to a red – where they need to be done. All the ones that I shoe on a regular basis for veterinary reasons, they were red ,and your normal shoeing was amber, but if you went another couple of weeks they would become red. So it was a fine line. But everyone was quite happy, understood why. A couple fo people cancelled because they were shielding because they had a cough, so just to be safe. I had one customer cancel because she had been to her grandfathers funeral and two of her cousins were nurses. So she said just postpone me for two weeks, and that makes sense, and she was being sensible. I would rather than than I turn up there and she had symptoms and didn't say anything.

AW: So you must have a bit of faith, and trust, in your clients.

F4: Yeah. There is no one where I have felt has invaded my space or come too close. It's not the sort of job that you are face to face anyway. The majority of the time you are 2-3 meters apart anyway, and you are just having conversations as you work. You do it, but you don't realise that you are doing it. It is just one of these natural things that happen. Its not as if we are standing a foot apart having a chat at any point- we have never done that or been that close.

**AW: Was it similar to the scenario during the foot and mouth outbreak?**

F2: Well, I did have a lot of farm clients, and it was just safer not to go near them. We were taking shoes off – I was out in Fiji at the time and it came up on the news, and Aberdeen was obviously one of the places. I arrived back and phoned the ministry vet in Inverurie to say what should and shouldn't I do. And he just said, stay off farms basically. People who lived on farms were perfectly happy with that as they didn't want anyone coming on their land anyway.

**AW: Do you think that having that experience gave you some confidence in your ability to control this situation?**

F4: I think because I was brought up on a farm and it is just common sense- there were things that you did that you didn't think about, and I think that that has carried into working life.

**AW: Do you think that the owners had an equivalent level of guidance given to them that they could employ?**

F4: I think initially a lot of them were very cautious, and chatting with them they chilled out a bit. But I think they were maybe being over protective. Some of them still wont go shopping, and you have to say, yeah you are safe enough. But you go to the shops, and other idiots who can't follow arrows are more concerning. You know, folk maybe have elderly parents and they might be shielding. I think generally everyone has been pretty sensible, certainly up here anyway.

And lots of people have wanted to carry on, because they are enjoying the peace and quiet!

**AW: Yeah, that is a good point, maybe people are enjoying the break from normal life.**

F4: And people have adapted, I have clients getting lessons from instructors down south. They set their phone up and they have really good microphones, and they speak to their instructors- and the horse is better behaved at home because it is used to that environment- they are getting better work out of it. But people are absolutely loving that ,and have said it is so much better. The instructor doesn't have to drive for hours, or fly up- they can sit in their kitchen, having a coffee, on their IPad or whatever, and just tell them what they need to do.

**AW: That's very true- now things might be a lot more affordable and achievable for some people.**

F4: Yea, for some people it can take an hour to get to the place for the lesson. Then you have an hour lesson and then another hour back home- so it is four hours by the time they get everything done. But this way, they can go out, get the horse in, tack it up, an hour lesson and 15 -20 minutes at the end. So in an hour and a half they can get on with other things, and they don't have to take four hours out of their day for a lesson. It makes quite good sense to continue doing it that way. A few of them have said, "I'm not going to go for lessons, I'll have one when it is easier for me to fit it in".

**AW: Has they farriery council been using the same means to provide CPD to farriers online?**

F4: Yeah, there are Mustad- a big shoe / nail / tool supply company based in Sweden- they have guys they employ to do clinics all over the world, and they have been doing online, on a Friday night making certain shoes. So it is basically an online clinic and you can ask them questions or whatever while they are doing it. Then there is a guy who does glue on shoes, and there are so many times that you can access the different processes of gluing shoes on. So the good thing is that it is there, so you can go back and refer back to it if need be. There are now online farrier competitions as well, so you make your shoes and then photograph them from different angles. Then you send them into a Facebook page and the judge looks at the pictures and decides the best shoes. So it is something new. You're getting guys from all over the world- and they come from their own smiddy at home. I think at 9-11am on a Saturday morning is the time you have to make your shoes- so everyone gets their shoes sent in at the same time. So it is a really good idea.

**AW: Have you noticed a difference in people's ability to handle the pandemic, between those who keep their horses at livery and those that keep them at home?**

F4: Yeah, I had one yard in particular- I was there on Monday night, no one said anything. Tuesday morning- complete lock down. No owners allowed on the yard- they were going to deal with it. One of the liveries – it wasn't even a client- phoned me in floods of tears because her farrier had refused to go up because no one was allowed on the yard to put a shoe back on her horse. I said, I am going back on Friday I'll pop the shoe on no problem. So I went up to the yard on Friday, and I spoke to the yard and said, you're going to have to watch what you're doing, because that was day 1 and a livery was already in floods of tears because the farrier wasn't allowed up and she couldn't get up to her horse, so you are going to have issues with that. (Their response was) no it will be fine. So they did that for a month, and then they tried to sort out other things for another month, and then 17 of their liveries left. Just because they had handled it completely…badly. Because he is asthmatic so he is at risk, his partner is a primary carer for her mother who had been diagnosed with cancer. So instead of them staying off the yard and letting owners deal with it, they just said- everyone is full livery. It backfired on them big-time. And then there are other yards where you weren't allowed to ride but you could come up and see your horse. A lot of these horsey women are quite sensitive and they need their daily horse fix. Its not until a situation like this arises that you realise just how vulnerable some of them are. I know a lot of them have good, high pressure jobs, and they come up to the yard and get their horsey fix, and they forget about work. They have to get that fix.

**AW: So largely we have worried about equine welfare, when actually the mental health of horse owners is really impacted too?**

F4: Yes, and the unfortunate thing is that on that yard, the horses weren't getting as well looked after. They weren't getting groomed, their extra bit of hay, and a lot of them lost a lot of hay, some were getting ulcers, just because it was too big a change to the routine. They just couldn't cope with it.

Just say, this is your time, this is when you can come up, the yard just handled it badly. They have opened it up again but they moved the yards about so that there were a couple of blocks of full livery where no-one was allowed in. if I went to shoe a horse, I had to find staff, get them to bring the horse out, ties it up, then I would shoe it and have to find them again and get them to put it back. So actually I ended up just buying my own brush, because they were obsessed about people touching equipment and stuff, so I just thought, I'll just buy myself a brush so I can sweep up after myself, rather than waiting for them to come and brush it up. There was a big fall out because someone touched someone elses tools and they weren't meant to. You think, really? It's a brush?! Just at the beginning, that is how obsessive people were being about everything. Without really thinking about

the issue needing to be addressed. Because I used peoples brushes, and would disinfect them before I put them back. But I just thought, it is easier getting a brush and just sweeping it into a pile and they can deal with it after that.

AW: A useful idea might have been to have a designated station where visitors to the yard had the facilities to self-manage and to adhere to the guidelines. Have you been at any yards which have had something like that?

F4: No. None of them. I got hand sanitiser and things from various clients. For the first couple of weeks I was using bleach for my tools and just gave my hands a quick wash with that- but after I while I got the hand sanitisers. Then, getting me a cup of tea- some said I won't offer you a cup of tea, and I said the hot water kills it – it's fine. So they would lay it down somewhere and I would pick it up.

**AW: Have covered all of the questions I have to ask, but is there anything else that you think is important to include in the conversation?**

F4: I think just the fact that some of these horses have become a bit feral because they haven't been getting the human contact that they would normally get. People might have checked them over, but not actually brought them in and given them a groom and a feed or whatever- because the circumstances didn't allow that to happen. You know, what is the point in coming up and doing all the grooming and the fussing if I'm not getting to ride it. You end up just coming up and making sure it is still alive and on four legs, so there just wasn't the same level of handling. And that did show in a few of them. Whether its just losing that discipline. Horses are creatures of habit and they like a routine, and the stricter the routine- a lot of them are much happier and content. I think it will take a while for them to get back into the routine again. One yard owner, he was moaning that "These bloody horsey women are so demanding, wanting to come up and see their horses, and wanting to dictate the times they come up. I'm a fisherman and my mate has offered me to go fishing. Anytime I wanted I would drive up there, on my own, and then stand there on my own, miles away from anyone while I'm fishing, but I'm not allowed to do that. So why should they?" I absolutely took his point- why couldn't he go and fish? You know? But the thing is that if something had happened, say he had drowned, that would put pressure on the NHS.

**AW: That is interesting. I have heard reports of conflict between those who have decided to keep riding and those who haven't. Is that something you have seen**.

F4: Yeah, I think it depended on what livery yard you were on- whether you were allowed to ride or not.

End----------------------------

Interview 4 HH

**AW: If you wouldn't mind, could you describe your day-to-day during the pandemic and the way you manage your ponies.**

HH4: I have to say it has been easier. Because I haven't been chasing about like a Whitsitt. I have been at home far more, the pony lives at home, he's here with me and another companion pony. So, I have ridden every day. I live in the middle of the country; I've got miles and miles of off-road hacking. So, I've ridden every day. his day to day management has been, for me, easier, because I have been here and not rushing about doing other things. The lockdown started a week after we got back from Australia, so he had been with another rider, being ridden every day. This is because at the end of October last year, he had a lesson on Monday, and on Tuesday he couldn't walk. To cut a long story short, two trips to the vets later, he had chipped a pedal bone. He was really ill, and he got colitis with all of the painkillers he was on and it was just horrendous. But they operated and he did his rehab and he was fine. Just before going to Australia he could start with riding. Since I've got him home, he has been sound as a pound, and fit! So, for me, it has been positive for my riding. I have been doing Zoom lessons with my chum who teaches me, so we were doing lessons when we weren't allowed to do anything. I haven't got an arena, but I've got a flat park, and for us it worked out really well. That's not to say its not been difficult- but getting food and things has all been fine because you can just phone up, order what you need and go and collect it. I have plenty of hay. So, it's not really impacted horse management. Apart from not being able to go to indoor arenas and competitions, it has actually not been an issue. We have been able to crack on with fitness and training. We were lucky that the weather in March April and May was fabulous, so we weren't; stopped from doing anything. I think the pony has looked as well as he has looked for years. Because he has EMS. They live in a small patch, and he comes into an even smaller patch at night where he can get access to indoors. He gets a fed and a soaked hay net and then he gets out again during the day. And he is looking great, and he has had no medication. So, I've been very lucky.

**AW: He you needed the vets?**

HH4: No, no, since he came back from the vets the vet came once to look at his foot. Well the vet came when they took the plate off, so when they operated, they drilled a hole in his sole to get at the bone. But he was sound as a pound. And the vet came to have a look, but I haven't seen them since. Apart from flu jabs- luckily, I got them done before we went to Australia. I got them done at the beginning of February. This was a month early but in retrospect it was a good idea.

**AW: Has the farriery been complex to manage?**

HH4: No, my farrier just came, the first time he came he wouldn't have a cup of tea. The second time he came he had a cup of tea- socially distanced. And he just gets on with it. I just tie the horse up and he gets on with it- not a problem. I also had the dentist last week- he comes up from the south twice a year. Although it was a couple of months later than he usually comes, it wasn't a problem and he just got on with it from a distance.

**AW: So, it sounds as though you had a robust plan in place anyway before the pandemic, where everything was in place to stay on top of things.**

HH4: Well yes but I'm very lucky in that the horses are here at home. I'm sitting in the garden just now looking at him so I know I am very lucky, if he was on a livery yard it would be a nightmare- I know people who are, and it has been very tricky. I have a couple of friends with horses at livery. One has moved her horses to her mum's place to get it away from a livery yard. With his pedal bone he was close to a hole in the ground. He had two MRI scans and eventually they found a little dark patch. But he way really ill and he lost so much condition. But the minute we got him home he was on the mend.

**AW: Does your companion pony keep well?**

HH4: Yes, he is fine, he is just fat. He doesn't get anything extra to eat. But he is absolutely fine he just gets the farrier, vaccines and dentist twice a year. Again, being at home all the time, I have been here all day everyday for weeks, so I can see them, and I can see when I need to move the fence a wee bit- to shut them in more. When it was hot the grass wasn't growing so I could give them a slightly bigger section. They are in a 2-acre field so when it started raining and the grass started growing, I just pulled them back a bit. So, it was very easy to manage, but then we worked at this management for 20 years. It works.

**AW: When you have had visitors, you will have been aware of social distancing and things – did you receive any guidance from equestrian bodies about how to manage people coming onto you yard?**

HH4: The BHS stuff was okay, the British Equestrian Federation stuff was useless, it was pretty confusing and not up to much. For the start they didn't seem to be singing from the same hymn sheet. Especially laterally the BHS stuff has been quite good. In terms of managing people coming, I think of it as my house, and the horses are just in the shed. You know what to do, you just keep away and say there is the horse you just get on with it. And you just pay by bank transfer or by check with an envelope- nothing was tricky because its only my husband and I here. I know there are much more people in difficult situations.

**AW: I'm sure it comes with its downsides, having sole responsibility?**

HH4: No that's good, that is the way I want it. I have never done anything else; I have always had my horses at home, and I've never used a livery yard. Now I can meet up with friends and go for a hack or go somewhere else to train and that kind of thing- we have started doing that a bit – just locally. Last week I took him over to a friend to ride in her arena. That was the first time I had done that- but he behaved like an idiot- I wasn't surprised because he hasn't been asked to go around in a circle since February. But he has been working well and progressing well.

**AW: Have you been based in Aberdeenshire for a long time?**

HH4: Oh yes, I have lived in this house for 40 years.

**AW: You must have a good overview of the equestrian industry in the region?**

**HH4**: Yes, I was on the BHS and Pony Club committee and things for many years- but I've done all that now, so I don't do it anymore.

**AW: Is there anything that concerns you in regard to the welfare of native ponies in the area?**

**HH4**: Yes, far too many are far too fat, and they get a surprise when suddenly they get laminitis. Encouraged, I have to say, by showing judges. It is my fault that mine got laminitis, he never got it badly. When he was young, I always liked to do a bit of showing. And you would go to shows and they would say, this pony could go to Blair if it had a but more condition. And we took him to Blair twice and he was 4th or 5th in the workers. Then we gave up showing all together and he evented, did dressage and show jumped as well. Now he does mainly dressage and a little jumping for fun. But there was this encouragement when he was younger to be fat. Of course, being a native he would eat until he couldn't' eat another mouthful. I should have known because his mother had laminitis as a young filly. I got her because she was about to be put into a hole in the ground because she wasn't sound due to really bad laminitis as a 5-year-old- after being at the highland show and being encouraged to be gross. I took her on, and I got a physio to her and it turned out her stifle ligaments had slipped. So, we got her sound and she evented, and she did great. These are Welsh Cobs. Again, that was just showing people encouraging them to look like whales.

**AW**: Do you think it is over feeding on purpose?

**HH4**: Well I remember the days when horses didn't get 24 different supplements. I think its all of these magazines encouraging folk to feed horses all of these different things, and also because so and so said its good for them. Oh, it has to have pink powder, this supplement and that. Plus, unlimited grazing which is often fertilised, and top-quality hay- which they don't need, and a reluctance to soak it. I was guilty of that as well, until he got diagnosed with his EMS. I was told, he needs practically nothing. When he was eventing for a couple of years, we were shovelling far too much into him to try to get the speed.

**AW**: Do you think the exercise kind of counteracted that?

**HH4**: Yes, but he didn't need performance mix and all the shit he was getting. So, I think there is a lot of that.

**AW**: Do you think that would be more prevalent that the obesity by neglect scenario?

**HH4**: Yes, well there is still a lot of that, but not amongst competition horses and ponies. They get laminitis through overfeeding- feeding of things that native ponies just don't need. I didn't learn my lesson quick enough. Now he just gets a feed at night; a handful of superlight top spec chaff, and a handful of farrier's formula for his feet, a glug of cod-liver oil and a tiny scoop of speedibeet to wet it and that's it. Then he gets only soaked hay- and its not brilliant hay- but that's fine because he is getting enough. He looks good- you can see his ribs- but you have to work hard at it.

**AW:** Yes, it sounds like it has taken time to get there?

**HH4**: Yes, well this is the third or fourth year of him being diagnosed but my vet was super.

AW: Do you think that with overfeeding being such a big issue, and people not getting to their horses so much- do you think we will see an improvement in them in that case? If they are not being over conditioned for competition?

HH4: I don't know, I think its habitual now. According to my vet there is a laminitis outbreak- and I think that is because people haven't been riding so much and still feeding the same. Or because of the weather- its not usually beautiful in April May and June. That meant the grass didn't grow till later, and people should have had their horses off it. The way we used to manage them was that you fed them a bit in the winter and gave them some hay, and in the summer, they didn't get anything except grass. If they were eventing, they might get a handful of chaff or pony nuts, but that would be it. But now because of all this advertising, it is very difficult for people. Especially younger fold reading al of these magazines. Their chum on Facebook says that this worked on their horse, or they say, you should try this. And I think there is a bit of that. You'd rather listed to someone on Facebook than the vets.

AW: A few people have noticed a great increase in people walking on normally quiet hacking routes. Have you found this too?

HH4: Yes, there have been a few more bikes. If they are local, they know to shout, but there have been a few more people walking, and a few more with dogs, but nothing that has been an issue. We don't have people being able to get to the gate or the field side-so we have had none of the people feeding horses and things. I have read about people with horses getting loafs and things over the fence. But it's an electric fence so they can suit themselves.

AW: That is great. I have asked all the questions I have to ask, are there any other points that you think are important to add in our conversation in terms of the impact of the pandemic?

HH4: Not really, I can see that people on livery yards have had a pretty ropey time, but for me my situation has only improved, I've been riding more the horse is fit. Apart from not being able to hack out with any chums- that was a bit of a pain, having to go on your own. But that's not the end of the world, you know? For me, because my horse will go without someone else- but for some people that would be tricky if the horse prefers to hack out with someone else. But we are not in that position at all.

END------------------------------------------

Interview 4

HL 4

AW: Could you please describe how you keep your horses?

HL4: Yeah, so I have one horse that I own. He lives at Aberdeen Riding Club, and Aberdeen RC is a very big livery yard. I think they have between 80-90 liveries, and they also have a riding school there and they have school horses there as well. So, the riding school has been affected as well as the livery activity as well. So, my horse lives out 24/7. He is a grass livery, and he lives in a field with four other geldings and they keep them all in sex groups and they try to keep them in groups who get along with each other, have similar needs and similar feeding requirements. So, our field is mainly a fatty's field, apart from one of the horses, but they give him plenty of supplementary feed to keep his weight up. But ours are the muzzling field, the muzzled boys! But they do try to put us in a field that hasn't got loads of grass thankfully! We have really good facilities at the centre. So, we have two indoor arena's- a big arena and a smaller one. And we have stables that we can use if it is raining. So, we have grass livery stables so that even though I haven't got my own stable, I am able to access a stable if I need one. We also have an outdoor sand arena and we have about 3 km hacking tracks around the outside of all the fields, which is fantastic as well. So, we can get out and hack around, even if we can't go off the premises, we can hack around the perimeter of the whole yard. So, I should be DIY really, because he is out at grass, and I just give him a feed when he comes in and I ride him- so I don't need the staff to do anything with my horse, but it ranges right through from people who are on full livery- so we have a lot of much posher horses than mine that are on full livery and have a stable and things like that. The staff do full livery.

AW: So, as we moved into the pandemic were you able to keep up that same level of autonomy on the yard or did your reliance change?

HL4: Well, yes and no! Because my clever horse decided he was going to injure himself! Actually, if he was going to do it, he picked the right time, in February he managed to scrape the back of his right knee with his other foot. The vet thinks he has done it getting up in the field, because it didn't look like a place where he could have been kicked by another horse. So, it was an upside down, V-shaped cut. This was before the lockdown anyway. So, he got stitches. But then the wound got infected under the stitches, so he had to go in overnight to the vet hospital and have it opened up and irrigated. Then he had to have the wound treated open, with antibiotics and creams and all goodness knows what. So, then he was on total box rest for nearly 3 months. So, that was a bit of a nightmare. Then of course lockdown started, so I was really lucky because the way our yard manager managed the situation was that she gave all the liveries a 2-hour rota slot to reduce the amount of people at the yard at any one time. We all were under strict instructions. She implemented a one-way system into the yard as well, you entered through one door and left through another, there was hand sanitiser everywhere, you had to wear gloves and you had to stay at least 2 hours away from others. We didn't have to wear masks because we were pretty much outside and we were socially distancing anyway. She made sure that because I was needing to see to Hugo having his dressing changed, so I would do his dressing in the morning, and one of the staff would do his creams and his antibiotics and stuff in the evening. So, we were able to do that daily. Then the period of time when I had to walk him out in hand, I was able to go up once a day and do that, and then a staff member would do it for me in the evening if anything needed to be done in the evening. So, I got my slot when it was good for me to be able to go up and manage him. Then he got turned out in a pen, and that was fine I could go out and set things up in the field. By that point if I needed to be up twice a day I could be. She said because it is a medical management it is fine. Just make sure you're not around when other people are around.

So, I was able to do that as I wanted to really, if I had to come up out with my slot to walk him or do something then I could do that. And I had him on livery then as well, so he was being mucked out and stuff. I would add in more shavings, but the staff would much him out.

Aw: You must trust the staff a lot to be happy to leave then in charge of him?

HL4: yes, definitely. It's a BHS training centre so they are all doing BHS exams. Even the staff who aren't instructors are very good with the horses. I've known them for a very long time. This is the third horse I have had at the same place. Yes, they have a very good reputation. We are lucky that if something isn't quite right, then you can just go and say to the manager of the livery staff, this wasn't done, and also they have a board that you write on, so if you want something extra done then you write on the board. And if its on the board then it gets done. It doesn't get done if its not on the board.

AW: So, you must feel able to bring up issues with the staff quite easily?

HL4: Yes, and they have responded to things when there have been problems.

AW: So, you said you had a 2-hour rota slot to fit things in?

HL4: Yes, you can't ride in an hour. So, the yard manager was very much of the opinion that you need to be able to ride your horse to keep its weight down and to keep it fit. I mean, the riding school horses had time off, and it was nice for them to get a bit of a holiday. But then they all started getting exercised well in advance of them knowing when they were going to lift restrictions. Early on they devised an 8-phase plan for the yard, so it was all set out. We knew what would be happening on a data by date basis. The goalposts moved a little bit, but they had thought everything through to when furloughed staff would be coming back, when we would be starting up lessons again. Initially we were allowed to ride in the arenas, but we weren't allowed to use any poles or to touch anything. Then a few months down the line they said yes you can use poles, but you have to wash them before and after, so they had buckets and fairy liquid next to all the arena and disposable gloves available, So that if you wanted to use poles then you could set them up. Initially we weren't allowed to jump because that was a potential danger. So, to take pressure off the NHS we weren't allowed to do anything that was potentially dangerous. But we were allowed to use poles if we washed them down with fairy liquid. And the liveries were asked to pitch in and bring fairy liquid- if you're in a shop and you see some then can you bring a bottle. It's a non-profit making club, so I think that goes some way to explain why it is run the way it is. Because the members of the club pay a membership, and all the liveries and riding school people are all members, so all the money made, any profit made, goes back into the club. So, there is a board to trustees and then, so the manager then gets a salary from the club and the staff do and everything else, that is kind of how it is run. So, it is not necessarily run on a profit-making business model. So maybe that is one of the reasons they have been able to be a bit more lenient, because the money is all going back in to the club anyway, so it is just a matter of shuffling the money around to cover what is happening at the moment. Our landlords are benefactors, it's the 7 trade of Aberdeen so they are a benefactor group of people who support small businesses that benefit the community. So, they are our landlords, and they have been really good and have let the club have a delayed rent and things like that. I couldn't tell you exactly what the deal is as I'm not on the committee, but I know that they have been very good and very helpful over the period to help to make us not too worried.

AW: It sounds as though due to the good programme in place it was quite easy for everyone to get on board?

HL4: Yes, everyone felt like they were pitching in together because we knew it was all for the good of our horses.

AW: Do you feel that your yard handled the pandemic well then?

HL4: Definitely, I don't know how else they could have handled it. They have done a great job. They have even been doing things like having training nights to raise extra funding. So, they have been having zoom training sessions so, someone would be teaching something about bitting, I did one on management of equine emergencies and equine first aid. So initially they were doing it for funds for the club, and then she learned about the BHS hardship fund – that they were raising money- so once they had set that up, the money that we raised has gone into that. So, they have actually raised b=money for the BHS hardship fund, because we were in a, not too horrendous position, but obviously smaller yards would be struggling a lot more than we were. So, we have been, not only looking after us, but also raising money for other yards as well. She did Zoom pub quiz nights so that we could all keep in touch with each other- because we are all really sociable and we are all friends. So, because we couldn't have nights out, we had virtual nights out on zoom, and just paid a tenner or something to do it. Then all the money went toward to BHS fund.

AW: Do you see people struggling with the 2-hour slots and social distancing?

HL4; Yeah, there were people saying that their hours were changing and they were working next week, but whenever they said that they have been able to swap it with someone, and the manager has been flexible so that, people who are key workers, like one of my friends who is a nurse, if her shift changed for the next week it has been okay for her to swap It with someone. A couple of folks said that they weren't telling their work they were off the two-hour rota because it suited them so well! It meant that they had that time sectioned off to go and have their horsey time! When she lifted the two hour slots and said people can come and go as long as they follow the social distancing regulations and the two metres, and one way system, then lots of people were thinking- actually I think I will just keep my slot.

AW: That is very interesting, a lot of horse owners with horses at home have said that they actually have a bit more control over their time.

HL4: I am lucky because I don't have kids at home, so I'm not doing things like home schooling, I'm sort of half working from home, but I'm lucky that I can flex that. But the manager tried to make sure that key workers got the evening slots, and everyone else had to do daytime slots. So, the keyworkers got the slots- they were prioritised.

AW: How did you find navigating vet visits?

HL4: It was alright actually. We just were distanced so the vet had PPE as well and if the vet had to come up at a time that was outside of my slot, then I was allowed to come up whenever suited the vet- so she was flexible about that as long as she kept away from everyone. Our yard is so big that it is quite easy. And because she knew at that point that there was only ever going to be four or five other

liveries up at one time, so in a yard as big as that, you were always able to ride somewhere on your own ,and tack up somewhere miles away from the other liveries. So, when two of you were on to the way to the tack room at the same time, you just stood back and waited for the other person to finish before you went in. So, because it was quite spacious, it was easy to do it like that. Because she had everyone rota's into two-hour slots at the one time. She also allocated arenas for people to ride in and let us decide who would go where. So, it was very well managed. And luckily the weather wasn't too bad either so we could use the hacking track as well when the weather wasn't bad, and it is easy to socially distance on a hacking track that big, even when there are two or three of you riding on it.

AW: So, you must not have had any concerns for horse welfare on your yard?

HL4: No definitely not, even when I have to muzzle, to save me going up twice a day, because the fields are managed the way they are, there are three of us in the same field, there is always someone there to take it off. So, I went through a phase of putting them all on in the morning, and someone else would take them off in the evening. Because my slot was in the morning, it was easy to do.

AW: Did you have to manage farrier visits too?

HL4: We have been really lucky with the farrier, he comes with his apprentice and he ahs been really good at making sure that all of the school horses were shod in a timely way, and he has kept his normal farrier slots. He comes on a Tuesday and does the school horses on a Friday, and we have had plenty of slots with him. He has been keeping coming and doing all of the shoes. He has a separate little shower bay, and parks his van outside, so he was able to keep away from us all as well. So, the horses that were caught in for the farrier would be all in one area within the yard. So, he was able to work away with the horses, without us having to be near him. It is designed for this, because it is a really new yard. We have been there just about 2 ½ years now. It used to be somewhere else, and it would have been much more difficult as everything was really squashed together, whereas here it has been designed with everything in mind, what is the most perfect yard you can think of kind of thing. It also has disability access; we have had to really think about the layout and it being really practical and useful. So, it has worked amazing well in this situation.

AW: I would imagine that the design has probably been put together with things like strangles in mind?

HL4: Yeah probably, so there are some areas that you can isolate horses.

AW: I think horse owners are very hyperaware of the spread of disease, so it is quite easy to transition into thinking about a pandemic.

HL4: Yeah, it is like farmers with foot and mouth. You just really make sure everything is cleaned and kept away from everyone else.

AW: Away from your yard, do you have any concerns for equine welfare in the region?

HL4: Well I have a friend who was at t a different yard and she only had an hour slot, and she really struggled and had all sorts of problems. She was messaging me and saying oh, I can't do this, and I can't do that. She had problems when she wasn't able to go up, someone she had asked to do something for her hadn't been able to do it as they hadn't been able to catch her horse, so it has been really difficult for some folk I know, definitely. Some have had big issues, but I think the other folk I know, a lot of them, have their horses at home so they haven't really had as many problems. But she is actually leaving the yard as a consequence of how things were handled.

**AW: Do you think that your yard was at an advantage given the links with the BHS- is that where a lot of the guidance and protocols came from?**

HL4: Yeah, probably. I think some, a lot of it was BHS driven. But actually, also because it is a gold standard yard within the area, I think the manager was aware that people would be almost watching us to see what we were doing. So, I think she felt it was important to set a good example, and to support others, and she has had very publicly put on our public Facebook page how we were managing it, what we were doing and why. So that other yards could share, she very much put herself out there and said that if anyone wants any help/ advice or whatever, feel free to phone me and I will help out as much as I can. I will let you know why we are doing what we are doing, or I can help with tips and advice and stuff. We are sort of quite in the public eye, so I think she was really kept that we were seen to be doing all of the right things. Because there is this thing in the horsey world where people do bicker behind peoples back, so I think we take the view that it is better to be more upfront. This is what we are doing, this is why we are doing it, these are the guidelines we are following and all the rest of it.

**AW: That's really interesting, it would be interesting to know if your yard owner was taken up on that offer.**

HL4: Yeah, I don't know.

**AW: My last question is just to find out if you think there is anything else that you think is important to cover in the conversation?**

HL4: The one thing that did kind of give me and I know my fellow liveries a little bit of a worry, and also people managing horses generally, was when the vets weren't able to carry out routine vaccinations. You know, on the back of all the hassle last year with the flu, the equine flu, that was one thing that potentially could have caused a massive problem. Particularly if people were then starting to go out competing and were behind with vaccinations and things because of it. So, I would say that was the only other main blip that we had in terms of a hiccup or a worry over equine welfare. You know when the vets were saying that we can only do emergencies, and the routine stuff was possibly going to go by the wayside slightly- that was maybe a worry. But certainly, in my experience, I personally haven't had any other concerns or worries- just usual stuff really! Not anything particularly as a consequence of the pandemic. Because he was poorly in the pandemic and we still managed it.

AW: Some people have voiced concern for the later stages when people have been affected by job loss. Would you say there will be impacts from that?

HL4: Yes, definitely. I think one thing that has been a bit stupid have been the rules about coaching. Like, coaches can only teach two people from two different families in a day or something, and yet other sport can return? When you think about it, it is a very socially distanced, outdoor sport. A coach doesn't have to get near a person, and you could say that it is going to disadvantage competitions, because people aren't able to get access to coaching as much. It isn't really welfare as such, but for the more competition type horses it probably is. Because they aren't having the same level of coaching as they were having before. It depends on the temperament of the horse as well. Luckily mine is such that I could leave him in a field as long and he was muzzled, and he would be much the same after 2 weeks as he would after 2 days. But yes, your fierier types, it would be really difficult to get them going again if they haven't been able to be ridden over a period of weeks. Or those who don't know how to manage that with a horse.

END---------------------------------------------------------

Interview 4

V4

AW: Could you explain how the pandemic has impacted your practice on a day-to-day basis?

**V4**: Yes, well I guess at the start, we had quite a lot of difficulty deciding what we should or shouldn't be doing, and because it was a situation that no one had faced before. The first challenge I think was saying what we need to stop doing as normal, and that decision came from the vets and not the management, which maybe isn't, so much, well everyone was just confused with what they were doing. But in the time running up to lock down we sere still out doing vaccinations and teeth rasps and social distancing wasn't a thing. We kind of started to realise that we were not really happy doing this when we were meant to be heading toward a lock down. Initially it was deciding what is acceptable to book in and what's not, then the lockdown hit and we were only doing emergency work, and that really was just- if it can't be sorted over the phone with a video and some advice then that was they only thing we were going out for. But we kind of made a list, the veterinary bodies were quite slow to issue guidance, so all vet practices were making it up on their own terms, but the neighbouring practices- after I had had a chat with them we came up with a more even plan of what we were doing and what we weren't. We were doing any kind of emergency work, so colic, lame at walk, laminitis, wounds, I'm sure there is loads of stuff that I am missing but just generally horses that ere unwell. Anything that couldn't really wait. Then it got trickier. I'd say we spent around 2 weeks actually doing that, and we were spending most of our day at home, but a lot of it on the phone. I think I was on the phone for about 5 hours one day. I totalled up the time and thought-wow- that's why I have not got much done today! We are used to working with 4-5 vets a day, then we were really down to two vets a day. And we split the on-call rota between four of us that were not furloughed, whereas normally there is 8 of us on the on-call rota- so that was quite a big change. So that was for the month of April, then the practice went down to just 2 receptionists and 1 nurse, so we just went to much smaller staffing levels. Then come May, I think we did that as well, but we were doing more work. There were four of us working but 3 of them were part time. So, we were doing slightly more urgent stuff. We stared doing vaccines again because we realised we could do that fairly effectively with social distancing and the fallout from vaccines overrunning was going to be a big issue- most horses, well all horses can really be injected themselves- so we were just doing that for vaccines and doing a little more here and there. So, we tried to group together yards and areas that needed vaccines rather than going gout every single day or to the same area repeatedly. So, we did that, and then it got more back to normal. Because a set of teeth that need rasped in March can wait until April or May, but then you get towards June and really you need to see the horse and see to the teeth. We have been publishing guidelines on our Facebook page of what we were doing each week or two weeks, But the trouble is that people got wise to the fact that we wouldn't come out to a routine tooth rasp, and they were phoning up the practice maybe saying that they has a horse that was quidding, then you would get to the yard and you think, " Yeah, there is no reason for them to be quidding, I think maybe they were just a bit sharp", but they know fine well! We had one guy who said four of the horses on his yard were dropping food and there was no way! People just know what guidelines they need to fit into o get the work done. So, we got to the stage I think that in June, all of the vets, or most of the vets, came back, and now we are back to normal in really every area. Pretty much back to normal work, whether that is the right thing to be doing or not, I don't know! Because it is going to be like this for the rest of the year isn't it.

**AW: That is interesting that you said that the decision to restrict movement and practice was really driven by the vets. Did you feel pressure to make that decision?**

**V4**: Yeah, I would say that everything was open to interpretation, which is good in some ways I suppose. I mean, we were doing vaccines, but we were worried about actually openly saying that we were doing vaccines. There are a lot of these vet forums and some people were going crazy like, "How can vets say that doing vaccines is more important than human lives!?", and you sometimes feel almost like if you did too much then you were very open to criticism from other vets. But my feeling was that if we wait until June to do all of this routine work, we are going to have so much of a backlog that we will physically not be able to keep up with that, and stay heathy, or even take the extra two minutes to put a mask and glove on. If you are under pressure things can fall by the wayside. Had we left it longer before we started back, we were only going to make our situation first. As well as that, one of our neighbouring practices spoke with us early in the pandemic and very much wanted to approach things from a united front, like, well, one in particular- I had a client phone me and say, my horse needs its hocks injected will you come out and do it? And I said, no I'm sorry I'm not coming out to do that, you know, we are in the middle of a pandemic. I am more than happy to prescribe Danilon to keep your horse happy until we are at a stage where we are happy to do joint injections. Then I text her when we were coming close to the point where she could have come into the hospital and handed (the horse) over to one of our nurses, and we would then take the horse and do the work. We were happy to do that, so I text her and she texted back saying, it doesn't matter I got another local equine vet out and he did it"> And you are just like, there goes the hymn sheet we were all singing from! I think people started to capitalise on the fact that if they did more work than the neighbouring practices then there was a big window of time to take neighbouring practices clients which is ridiculous. We were very strict and only did vaccinations for our own clients who had been registered with the practice for a reasonable time. We were not, if another practice decided they weren't doing vaccines, we wouldn't take their clients and do their work. At least that we you keep the moral high ground a little more, and you're not focusing in and stealing other peoples work.

**AW: Do you think that horse owners were well informed about how to handle the pandemic?**

**V4**: The feeling that I have got is just that people haven't taken it that seriously. You know the same people you see on Facebook saying, "Oh my god I can't believe that people are doing this (not adhering to guidance)," are the same clients that when you are there (seeing their horses) they are literally one centimetre behind you! And you are like, what are you doing? My fear as a vet is that seeing 10 different people in a day, and at times you are in close contact with them whether you want to be or not, I was really worried that if I caught it and didn't have any symptoms, then the number of people I could have been in contact with in a week is crazy! So that is why I was pretty vocal about what we should and shouldn't be doing. But, you know, I think it depends little bit on managers who were very quick to decide what to do. But because our practice is owned by a corporate, I think it took the pressure off the management because they are being guided by what is coming down from corporate, and their advice is always a few days behind anyone else's advice. You end up just like, well I did, in the end I just spoke to the other vets, we agreed what we were happy to do, I said to the manager we are not happy to keep working as normal, ad basically just compiled a list of calls we were definitely not doing- so don't pass it on to a vet because we are just not doing it- the calls that maybe we need to discuss, like a sarcoid or something, collect the details and pass the call on to the vet and then we will decide where to go from there, and then the calls that, you know, don't faff about collecting details just pass those straight through to get us on our way. So, I think we did have quite a clear structure of play, but I certainly wouldn't say that that came from any kind of, you know,

the RCV and the BVA. Those are the two governing bodies and their advice was very slow. Their list that they produced was about a week or two after ours, and it was pretty much the same as what ours was. But it was way too slow. But then, I think, can you criticise anyone when it is a situation we have never been in before? Probably not. Us making a list of what we were happy to do was fine because it was just for us, but I am sure if you are trying to make a list or guidelines for the whole UK then maybe it is a lot harder because there may be more repercussions against the decisions. But yeah, there was definitely a clear lack of guidance, and I think clients, not all, I mean I've got loads of brilliant clients that I love to bits, but people are selfish. That is actually what it shows. You would have girls, like that girl phoning three or four times a week because she thought she needed her horse's hocks medicated. And you just don't, you think we are just not wanting to do the work because we are lazy or something! There is a real feeling of, I want this done and I want this done now, I don't care that there is a pandemic, just come and do it. And I don't know if that is because they were not well informed, or whether people, well my gut feeling is that people just kind of stomp their feet and get what they want in today's society. Probably it was just an extension of that. I think the big problem as well, was that because most people weren't working, they viewed it as this brilliant opportunity to make progress with their horse and to do all these things they wanted to do like the training at home. This girl kept saying, I really want to work on things while I'm off work, and if I don't get the hocks done then I'm not going to be able to do it. And I think part of it was people on the yar. Some of the yards obviously were closed down and that was actually probably easier, and it was just the livery yard owners looking at them and they were only calling us out for something serious. But where people were still able to see their horse all the time, they were like, well I'm off work for the next two months, I want to get going! Yes- but there is a reason that your off work, you're off work because there is a global pandemic, so I don't think that perfecting your half pass is really that important in the grand scheme of things! So yeah, some people were able to spend all of their time all day with their horses, so it became even more of a focus for them, more than anything else in their life. Whereas if they had work and stuff going on, a couple of weeks wouldn't have been that long.

AW: Have you felt that people have been calling more about things they might not have usually noticed?

V4 I don't think more than they would have usually noticed, I think one big problem we had with respect to laminitis was that because the weather was good, quite a few yards- their solution to not having liveries up was just to chuck all the horses out in the field. And some horses I have seen post lock down, I have never seen them so fat, ever. Horses I have known for 6-7 years, I have said to the owners, oh my god what has happened? They have put on so much weight! And they have said, well for two months I haven't been allowed up to the yard, or I have been allowed up to literally come and pat it and check it over in the field. So, you have got a horse that is normally stood in for most of the day or night, that is then allowed out 24/7, plus it is not getting ridden, plus it is not getting checked over so much. I would say that some I have seen have put on 100 maybe 200 kilos, this spring that they maybe wouldn't have put on. Maybe 200 is a bit extreme but I am thinking about one in particular, that maybe had already wintered quite well. There was one little Connemara cross Highland that is normally sitting around maybe mid- 400 kg, a fit little allrounder pony, and it was shocking. But then what can you say to the owners, because the guy was pretty devastated, but I was like, you are going to have to do something about this now. You know what it is like, it is hard enough to shift the weight off them even when things are optimal, so to try and shift the weight off them when they have already gained that much is really hard, I guess.

**AW: Do you think it would have been better to have specific guidance for owners with obesity and laminitis prone horses?**

**V4:** Yeah, but, when we were in April / May time when we were on a more skeleton staff, we knew there was a problem with grass sickness and laminitis, because there always is at that time of year. So, we knew it wasn't great. In previous years we would have done a big post, please be careful of laminitis, please be careful of grass sickness, you know, don't do this, don't do that, but we didn't do that this year. I think if we had published something like that saying, be aware of laminitis.

*Call cut out at this point*

**AW: So overall, what do you feel has been the greatest impact on the welfare of the animals you have attended?**

**V4**: I don't think that, other that the overweight, much higher risk of laminitic ones because people were unable to ride, I don't think we really saw a huge amount of welfare problems, in that vets were still attending if there was a serious problem. I guess you could argue that somethings were left longer than they maybe should have been, but certainly in my experience if there was something that I wasn't seeing, I would have had it on painkillers, I would have still have been managing the welfare. So I think overall the biggest thing was that largely the big livery yards we dealt with were kind of closed to the owners, which meant that they were having a lot less checks done on them, but then actually, they all seemed to be okay. So, the main thing I think really was the weight gain issue. Which probably came more form the riding than the management.

**AW: And with the reduction in riding and maybe checks, have you noticed any behavioural changes in the animals that you were seeing?**

**V4:** Ehm, I don't think so, but to be fair, if they are bad, they just get some sedation so probably we don't see that as much. Some people did say that they were out of their routine, so if you were bringing them in for a vaccination they were maybe a bit anxious because they haven't been in as much and its out of their regular routine, but I wouldn't say that I have noticed that really. But then vets probably have a very short tolerance for it, you know we would just go to the car and grab some sedation and its problem solved.

**AW: So, will have been taking a lot more calls than usual, do you see yourself implementing that into your practice more after the pandemic?**

**V4**: I definitely prefer face to face visits. I think maybe there was a remit to stay, like normally all the vets would be hanging about the practice, but now if I had a spare hour I might just go and write up my notes in the house and email them in. So maybe that (will be carried on), but I wouldn't say there were any big positives to come out of it (the tele-consultation). Because trying to look at a lame horse on a video call or trying to take an owner's word for it is an absolute nightmare. You know, people will phone up and say, I think my horse is a bit laminitic, so they will phone in , but looking at it you can't really tell, is it tied up, is it a muscle issue, is it a laminitis issue- so you would want to feel the pulses to the feet and you can't. You ask the owner to and they say, I don't know how to feel the pulse, so you are talking through it on the phone! So, I think given the chance we would all rather go out and just see it. I think it has made us realise that there are maybe some things which, on a quieter day we

could be doing that at home rather than sitting in work all risking infecting each other. But really just now there are no days like that anyway! We are pretty full on at the minute. The weirdest thing was we would normally ask people to stay and hold the horse, and then during covid we were like, yeah just step away and let us deal with the horse. So, I think it was a surprise to see how easy it was to do without the owners there, but health and safety wise there were a lot of discussions over whether that was something we should actually be doing. But that is what we did. But yeah, I don't think there are many positives to come from it.

AW: And was there any point where you felt your health and safety was at risk?

**V4:** Yeah, I think quite often to be honest. You turn up at a call where the horse needs to be euthanised and every man and his mother would turn up to say goodbye to the horse. I would always wear a mask if I couldn't socially distance and to be fair, I was thinking when the phone cut out, have I actually worn a mask recently? I don't think I have but I think out of sheer luck I haven't actually been in a situation where I have been that close to anyone recently. You know, I think when you are in a situation where you just can't socially distance, or if you are in a position where you can socially distance but people are just hanging around right beside you, I think we have all probably though, we are really at quite high risk because we are in a front line position, and in contact with a lot of people in a day. But it is quite hard to maintain distancing. I think it feels a bit better now that everyone is back to work, because you feel like, we are all in the same boat now. But then maybe that is when the risk will be higher, you just don't know! But yeah with everyone being back to work the chances of picking it up are much more likely. I think a lot of the yards that closed down were really focused on the health and safety of those coming on to the yard, an only having one or two on at a time. But then that presented its own risks. I think the tricky thing is that in an emotional situation everything just goes out the window. I know that from personal experience, because I lost one of my horses at the beginning of lockdown. I had been really careful with my mum and dad and the something like that happens and it all just goes out the window. So, I think in that situation it is really hard for us to bring that up because it (social distancing) is the least of their (the owners) worries at that point. But we know we are leaving that situation to then go and deal with another 10 people that day and potentially be spreading it. So, it is kind of having to subconsciously take a step back or in the back of your mind, without necessarily vocalising it and making a bad situation worse, you just have to be thinking about it in the back of your head. You know, try to keep everyone safe. But there was one horse that needed to be put down which had gone down in the field, and the only people there were an elderly, 80-year-old couple. So, we, I think we had three vets, go there and although we were near one and other, at least we weren't near the couple because they were both shielding. Stuff like that where it just adds an extra element to the whole scenario is tricky. You get people who are taking it very seriously, and others who aren't taking it seriously at all, so you just never knew what you were going to turn up to.

AW: Did you have some sort of screening procedure in place?

**V4:** We did have a sort of screening procedure when people called in they were asked, " Are you shielding?", or whatever, but I think the trouble was that because there were only two of them working, and they were working split shifts so that they weren't overlapping in the office, then – you know, I know what its like in there sometimes when the phone starts going crazy it's just like a free for all basically they are just trying to get off one call and on to the next, so yeah I think a lot of it is just

responding to stuff as it happens. We are quick to moan about stuff but if you sit in there for two long, you are like, “Yeah, I can see why things are tricky, it’s a difficult job”. If someone phones to make a payment, I am just like, “Yeah, okay just phone back later- I don’t know what to do!”.

AW: Is there anything else you think is important to cover as part of our conversation?

V4: No not really.

Interview 4

WCM 4

**AW: Firstly, could you describe how your day to day has changed during the pandemic?**

**WCM4**: Well on an operational level, we function as a rescue centre but also a visitor centre. So, the most immediate, noticeable change is that we are closed to the public, so the place feels really different. Just under half of our team are furloughed so there is the obvious day-to-day impact practically. On the horse side of things, I feel a bit like this is the calm before the storm and we have not actually seen the impact on welfare yet. Lots of people are furloughed, or working less hours, or working from home, so everybody wants a horse. For us, there is a really big demand for rehoming, and we have actually rehomed more horses to date this year, than we did in the first 6-7 months of the year last year. So, for us, our stocking level at the start of the pandemic was something like 98-99%, and now we are on mid 70's and only one of those was a euthanasia. So, rehoming at the moment is absolutely flying. We literally just can't rehabilitate horses fast enough. We fear that this is the calm before the storm, and the pandemic hitting through a lovely warm, dry early spring- it is an easy time to keep horses. It is an easy time to get by, whereas if it had happened in November, I think we maybe would have seen a more immediate impact. Because it is a lot different slogging around in the mud and the rain, and horses are more expensive to keep in winter than in summer, so I think we are going to see a huge demand for spaces in our centres- but we haven't seen that yet. But it is coming. When the furlough scheme ends, in October here ( I don't know if Scotland, Ireland or Wales are doing anything that England is doing at the minute it is all so disjointed), I think then- when peoples financial circumstances change- there will be a huge demand.

**AW: What is the type that you expect to be rehomed?**

**WCM4**: I think, maybe I am a bit cynical as I have been in rescue for too long, but I think that horses who have higher value ( in terms of use – those who can ride and drive and carry granny)- those types of animals are lucky because they are so versatile in terms of use, not necessarily in terms of monetary value, but I think those often don't find themselves needing rescued because there is always someone out there who wants a nice riding horse. The ones who are less versatile, less valuable, older ponies, ponies or horses that have been retired from work or retired from competition with injury – you see the classic examples on social media all the time, "Free to a good home, can no longer be ridden, might be able to hack out occasionally". Those ones fall between the cracks pretty quickly. Anything that is older, unsound, injured, or difficult temperament ( an we know that difficult temperaments either means they have an undiagnosed injury or have had completely inappropriate previous training) those things can ordinarily be overcome, but they do need to go to an experienced home and there are not that many people out there who like a quirky difficult horse when they can have a nice safe one. There are some people who like a challenge, but there are not that many of them. The reality is that there are more horses than there are homes, so the least desirable ones end up needing a space.

**AW: In terms of rehabilitation, have you been impacted by the pandemic in a way that has limited your ability to rehabilitate as normal?**

**WCM4**: We haven't really. Our chief executive took the decision at the start of lockdown to stop us from doing all rehabilitation work, and that was based upon health and safety. He wanted us to be as risk averse as possible. And as a manager, I didn't want to be the person driving someone to A&E with a broken finger, or a broken toe. Because we know that the serious injuries happen and of course you have to go to hospital, but the stupid, petty injuries also happen every day, and you still

have to go to hospital. So, we elected to take all of our horses out of work, and to provide basic, primary care only. And that lasted for a period of about 4 weeks, and gradually as restrictions started to ease, we got the go ahead to begin groundwork, and basic lunging and long reining with our more established horses. Then we have gradually progressed back towards normal full rehabilitation work with all horses, but still with caution in mind. We always have caution in mind anyway, but we are just double, triple checking, that the horse really is ready for the next step- rather than saying, “Lets have a try and see how this does today”. Just being more thorough, which is no bad thing really.

**AW: That sounds great. Have you been in a position during the pandemic where a horse than normally would have been a candidate for rehabilitation, hasn’t been suitable?**

**WCM4**: No, it hasn’t. We have been really fortunate. We have certainly had delays, for example we have a stack of 18 colts which have been booked in for castration since the week before lockdown. So, it has delayed the rehoming process, but it has been in no way catastrophic, but it hasn’t forced our hand in that way at all-, but I think that is luck with the animals that we have in the centre. I can certainly imagine that that could be the case.

**AW: That must have been difficult having 18 colts on site!?**

**WCM4**: Yes! We had them all put aside for a BEVA castration course, so we had deliberately queued them all! But in the grand scheme of things, it doesn’t really matter. They are all done now so that’s good.

**AW: How do you feel like the community Is dealing with the pandemic in terms of health and safety?**

**WCM4**: I have certainly noticed on social media a feeling of… I don’t know if people think they are invincible, or if they are not understanding the severity of it. Or the attitude of, “My horse is really quiet, so I can still ride today and that is my 30 minutes of exercise”. It seems to be polar opposites here, where half the people are completely shielding, and have not left their houses, and the other half are carrying on like there is no pandemic at all.  I feel that we are sitting in the middle of the road and are trying to be really be responsible and set a really good example for the wider community. Actually, a global pandemic is much more important than whether or not Bobby goes for a hack today- but equally not being so terrified that we can’t function.

**AW: In terms of laminitis, have you been aware of an increase in cases?**

**WCM4**: We have been pretty level, I wouldn’t say that it has made a difference, YET. We haven’t seen a significant difference, but I wonder if that’s due to being where we are in Somerset, we had a phenomenally wet winter followed by a phenomenally hot, dry Spring. We were in excess of 30 degrees in April, so the grass down here just hasn’t had a chance. Even our fields, which we rotate and look after as best we can, we won’t get hay off our site this year. An that is just weather conditions driven by going from a monsoon to a heat wave. So, there is very little grass growth compared to normal. We always have a few fatties that trundle into the centre in the early months of the year, and actually they are all responding really well to normal management. But I do think that is quite localised to our little pocket in Somerset, and actually we would like the grass to grow a bit more really.

**AW: Do you think the pandemic will have an impact on laminitis cases?**

**WCM4**: Yeah, I think that anyone who has turned their horse away, who has been responsible, but has failed to understand the changes in Spring grass and workload, is potentially going to set

themselves up for laminitis. We have not seen it here, but then of course we still have the luxury of a horse walker, and 200 acres of land to play with- so it is easy for us. But the average owner who is at a livery yard, and is told, “There is your summer field and there is your winter field”; that could be really tricky if it has come in conjunction with a reduction in exercise. Because what do you do instead? You can’t ride the horse, so you have to do something to reduce calorie intake.

**AW: Is there anything else you think is important to include in the conversation?**

**WCM4**: Ehm, I just hope that in the long run, more good will come from it. I think people who have animals during the pandemic has fared better than those who don’t. Everyone who has a dog has commented on how happy they are to have a dog- the excuse to get out and about with them has been good. People who have been flat bound with a cat maybe feel more fortunate than people who are flat bound without a cat. I think we have seen the same on the horse side of things, where people really value the fact that they do have a reason to go out in the morning and poo pick. The stuff that we maybe used to see as a bit of a bind or an inconvenience, suddenly becomes the highlight of the day due to the pandemic. And I hope that people don’t lose sight of that.

Interview 5: HH5

**AW: So, if you wouldn't mind would you possibly kick us off just by introducing how you've been going about your work and your day to day horse management during the pandemic?**

HH5: Um, Oh, right. Okay, so, um, I pretty much had to shut down business from the beginning of lockdown. And it got a little bit quieter on the lead up to it and obviously, you know, social distancing and things like that started to come into play in the sort of weeks before actual lockdown. And when the day that lockdown happened, I’m asthmatic and also the my main money comes from holiday cover. So obviously no one was going on holiday. And my few regular jobs I have either got furloughed from the minute of lockdown or, and shut down the yard and sort of did the yards themselves if that makes sense. So, I just had one sort of job walking a dog. Right. And for that for the whole time for a key worker. But that was it. Yeah. So basically, I just had however many weeks it was I can't even remember, not working, which was not ideal.

**AW: Yeah, that sounds very stressful. Actually, that's the most that anyone I've spoken to has been impacted actually, by, by the pandemic.**

HH5: Oh, really? Yeah. So, the other problem was that I only started up my business in 2018-19. And I only had less than six months booked and so the money that I got from the government was less than a month's wage for the three months, I wasn't working, if that makes sense. So yeah, it was, it was a bit a bit tricky.

**AW: Have you got horses of your own that you need to look after as well?**

HH5: Yeah, so I have three at home. And luckily, they're at home. And so actually, you know, in terms of sort of my well actually my mental state when I wasn't working, they definitely kept me above board and kept me busy and things like that. I have done a lot of groundwork with the youngest, because he needed to be backed and things like that. So that was that from that point of view. I carried on riding as well not I didn't do any jumping or anything like that, but I carried on riding. Yeah, the other two that kept me busy. So definitely a godsend having them at home.

**AW: Did you make the decision to continue riding for their mental wellbeing as well?**

HH5: So basically, for all of our mental wellbeing at the time. You know, one of them in particular well actually both of them turn into hooli Wiley's if they're not doing something. One of them just annoys the other horses in the field and you know, he's constantly chasing them around the other one just gets a bit bargy and a bit fed up. They're both, both my horses are absolutely bombproof to ride you know, I wouldn't have gotten on anything I didn't trust. And that's why I didn't get on my younger one. I just did all the groundwork, which was, which was actually great. That gave me sort of six, eight solid weeks of doing some pretty intensive groundwork, which was great for him. Yeah. But yeah, so yeah, it was for both of us. Really. Yeah. Well, all of us. Nothing spectacular, but just to keep us going.

**AW: And what type of horses do you have?**

HH5: I have got a warm blood cross and a Clydesdale cross and a full Clydesdale.

**AW: Oh, lovely.**

HH5: The full Clydesdale is four, he is the one I have just backed. So, the pandemic gave me the time to work with him. And then I event so I wanted to keep him up to sort of a level of fitness. And then the other one was just to keep them ticking over.

**AW: Have you been able to access farriers and vets and things like that as you've needed?**

Yeah, so luckily my farrier carried on working within the guidelines. So that wasn't a problem. And because, you know, I'm a very small, private, isolated yard. I was just I was pretty isolated myself. They were perfectly sort of safe to come here. Yeah. And the vet, luckily, I didn't need. But our vet was working but only for emergencies. I believe that when I think it was, you know, was being a smaller yard, it wasn't too bad. Yeah. And I didn't need any vaccinations during that time. I literally just got mine done the week before.

**AW: That worked out nicely. Did you kind of just tie your horses up and leave them to it? Or do you need to hold yours? or How did you manage that?**

HH5: I just tie them up and then sort of leave them to it. Like I say they're pretty well behaved. So, I'm quite lucky in that respect.

**AW: That's brilliant. Because that's one thing actually that's come out of these conversations that I really didn't expect, many have noted quite a change in behaviour of horses. They've been finding them a lot more difficult to handle either due to people not riding or due to, you know, yards being shut and people not being able to handle their horses as much. Is that one of the things that helped you to make a decision just to keep going with your horses' training?**

HH5: Yeah, absolutely. And I also you know, I have a lot of friends that livery all over Aberdeen and things and I know the biggest problem with the whole of this lockdown the pandemic has been that the ways the big yards have run, some of them shut completely. Yeah, you know, for four tor six weeks and the horses, you know, weren't getting any sort of daily exercise or training, particularly the ones that were out in the field and things like that. And, and I know that it's been a real problem for those people and also for those horses, you know, behaviourally bringing them back into work, keeping the weight off them, laminitis, you know, all that kind of thing, it's been a huge, huge stress. So I feel like there's been a sort of an out now divide down the line where people have got them at home, you know, we've been able to, you know, micromanage our horses, you know, really sort of do things, you know, almost spend more time with them. But then people with horses on livery have just had the complete stress of, you know, trying to manage their horses in whatever way they can with maybe only one hour a day or, you know, in some cases, you know, five weeks without even seeing them, yeah, you know, during spring time. No, no, I feel very lucky that I could, could just carry on as I was, I suppose.

**AW: I feel like you've just summarized all of the conversations I've had with people because I assumed that people who kept the horses at home would have had difficulties and you know, maybe getting people to move their muck heap, getting hay and feeding and things like that. And it maybe it has been quite difficult for them. But I don't know if that's been the case for you, but certainly all other**

people I've spoken to have said, it's been kind of great, because it's meant they could spend more time with their horses, they can do what they wanted to do.

HH5: In terms of things like that, you know, like the farmers have all been working, you know, I've had a delivery and you know, the guy just came in and threw it off the truck and, and, you know, the yard and things like that. So, it's not been difficult, certainly for myself. I think people that have been furloughed at home and things like that have probably actually been able to manage their horses better than they normally would. Yeah. Like you say, one good thing that came, you know, I didn't think I would have time this year to back my Clydesdale, because I thought it'd be too busy eventing and working and just kind of thing. So, I'm actually kind of like, every cloud, you know.

Yeah. And that's good.

AW: That's great, it's good to take the positives out of it. Have you noticed any changes in the delivery of care and management? Do you think that's been something that is a level yard issue as opposed to overall horse population issue?

HH5: Yeah, I think so. I think I mean, I think a lot of the people if they've got their horses either on smaller yards or in a field somewhere or what have you, they were they were still you know, you were still allowed to go and see to your livestock and I think people were making, you know, probably, as I say, you know, maybe possibly inverted commas breaking the rules and going to see them more than once a day and what have you and things like that. Yeah, I know, the livery yards, you were not allowed to go. I know of a couple of instances with these yards where you know the horses you know have maybe that when when it's coming to the owners being able to go back they've had their withers rubbed raw by a rug or they've had, you know, something that's not been noticed or you know, like there's been quite a few little things like that that just haven't been picked up for a number of weeks. Yeah. Because I'll do one or two people look after 60 / 70 horses you know, and I this is the bigger yards you know, like the smaller ones it's you know, the level of care can be better, whether or not it is, but it's quite tricky to look after that volume of horses to the same level as their owners would obviously but yeah. I've definitely having spoken to a few people have there have been some instances where sort of things like that have happened and it's a tricky one.

AW: Yeah. I worked on livery yards for 10 years or so. And for me, I kept in touch with the main livery yard I was associated with, and they did complete lockdown. I was thinking that if I was there, that would be great- I bet the staff are having a blast without having clients to worry about, and it probably would have been a bit easier. Then seeing the sort of impact that this is had , I've seen that maybe welfare standards have slipped at these bigger yards. Maybe without owners there, horses have not been as well looked after. It definitely made me respect the role of the owner in the yard more than I than I did.

HH5: Yeah, yeah, absolutely. It's tricky. I mean, you know, and with all the best will in the world, you know, I'm sure they tried their best, but it's just not possible, you know, always to look after that volume of horses, but only maybe two or three people or something like that, you know? Yeah. When you've got all the other things going on and you're having to do everything, like everyone's stables and manage everyone's area visits, you know. Yeah, it was it was really hard. I think it was really hard for the staff particularly have a lot of yards you know it, it doubled and tripled their workload and a lot of cases. That's a long day!

AW: So, obviously you've got quite a far reach with your work in the equine community. Were there any horses that you looked after that you thought, this pandemic is going to impact them in a bad way, that you were maybe quite concerned about their welfare?

HH5: Yeah, so, Oh, yeah. So probably, probably, again, you know, yards that shut down with friends with horses, particularly laminitics, once in all fairness. I think I think for people with any horse with EMS or laminitis, or anything like that, that really need to be micromanaged and worked. And I think for those people, and those thought that those were the ones I was more concerned about. And you know, I had a few conversations with several people during it, trying to think of ways that they could manage it differently or you know, how they can sort of work around things to try and keep the horse safe. While You know, being in a situation where they were only allowed to see it for an hour, half an hour a day and not exercise it, like you said and things like that. Yeah, that probably would be a welfare issue, if that makes sense. Yeah. You know, sort of, you know, all that one could do with it, because it's behaviour is a bit rubbish and stuff like that, but Well, I think, I think anybody who was concerned about laminitis, probably was the most stressed out during the period.

**AW: In those conversations that you had, what was the conclusion? What did you think was the best way to approach managing a horse with laminitis if you're restricted to an hour?**

HH5: Well, that's the thing. So, I mean, it was it's pretty difficult. I mean, there was one particular person who was being told by the yard people not to muzzle it yet because the grass wasn't really coming through yet. And I was like, just muzzle it. And that, you know, it was April, middle of April. It's starting to rain, you know, the rain was starting to, you know, bring the grass through and I was like, it's time to intervene. I got sent pictures of the field and what have you. And I was like, Look, she's coming in at night she's getting hay net. Yeah, put a muzzle on during the day. You know, it doesn't matter, because she had an awful bout of laminitis two years ago, and they micromanaged it last year and she was great. But she wasn't, you know, she was having eight weeks not in work. They weren't even allowed to lunge them or long rein them. And they'd been always kept as Super lean and super fit. And they were now being told, no, you can't, you know, can't do this. Can't do that. But don't muzzle her. I was like, just put the muzzle on the poor thing. You know she can have her haynet at night, and you know, she'll be able to pick away during the day, but it was just sort of things like that. I mean, that was really the only thing they could do. Yeah, really. And they weren't a Put her in a smaller park at that point, they were still in their winter phase that you know, but the grass was shooting up. So, and in that particular instance, that was all they could do really was put their foot down and can say, Look, just put my muzzle on my pony, because obviously they weren't there to do it themselves. Yeah. And I think that sort of quite sort of a difficult one.

I think I always like to err on the side of caution is like, the way with them is to manage them like they're gonna' get it tomorrow all the time. Yeah.

**AW: Yeah. Preventative definitely. And I think that's I hadn't considered that but actually, that must be a really difficult conversation for liveries to have with livery yard owners or managers who they probably have quite a lot of respect towards. And then to have to force them to say, this is what I want done with my horse, I'm paying you, you need to do it. It's quite a confrontational situation to be in that people may be worried about and not really emotionally prepared to be put into.**

HH5: Yeah, yeah, I'm particularly, you know, like I say, in these instances when they're worrying about the horse welfare in particular. And earlier on, you know, when it first locked down, and everyone was like, crazy emotional, and it was all a bit sort of, wow. Yeah. And as you say, you know, like, they have great respect for the livery yard owners, but sometimes you just have to put your foot down because it's your horse at the end of the day. As long as you're not creating the welfare issue, you need to be

able to say this is what I want done, and that's it. There's a lot of tricky conversations. I know quite a few people left various yards and things like that over the course (of the pandemic). But I think it was just tough for them. It's tough for everybody. From the horse owners looking in and from the owners of the yards looking out. Yeah. And what was the best thing to do.

AW: Obviously you've not been able to go out and speak with your clients? Did you end up fielding quite a lot of their concerns and helping more over the phone?

Yeah, yeah. Yeah, I spend quite a lot of time on messenger or on the phone and what have you over the period which is fine and it's good for me because it made me gave me a little bit of a feeling of worth. Without it I could feel, pretty useless.

AW: So, you're actually still providing a service. But that Yeah, you're not getting the paid for that service, which is quite a difficult situation to be in. Maybe during your usual work hours, you would you wouldn't charge for that because you know, maybe you'd be seeing the horse in a couple of weeks' time anyway, and so a short telephone call isn't a bad thing, whereas when you're kind of restricted to being at home, maybe that telephone call is actually a service really, in its own way, so that must have been difficult to navigate as well for you.

HH5: I don't mind, I do quite a lot of random telephone calls and stuff like that for free even with people that I don't see. So, it's kind of, like, quite normal. It's normal for people to just ping me for advice and sort of do bits like that, and, you know, go into bits and bobs. So it was, um, it was, yeah, it was fine. like to say it. I didn't really sort of I didn't actually see it like that, in a way, you know. Yeah. It totally does make sense. Yeah. It's not really how I saw it at the time, I would like to say it was nice to have the feeling of being needed. Well, that makes sense. Good for me as well as hopefully useful for some other people.

AW: That's great. So, we have touched on mental health a couple of times. Another thing that's coming up with these conversations is that the mental health of horse owners has been gravely impacted by restrictions. You said you've been able to work with your horses and use the companionship to keep your mental health in a good place. Have you noticed a decline and sort of mental health in the in the community?

HH5: Yeah, I think I think it's picking up again now like during certainly during lockdown and the more of the restrictions and I know some places are still quite restricted. And actually, some people's mental health is helped by competition and stuff. So that's I think that's still riling up some people a little bit. It doesn't bother me quite so much but um, during the lockdown, I was very careful had to be very careful with what I posted on Facebook, like I try not to post too many pictures of all here's me riding my pony in this range. I know that that kind of thing actually really set off quite a few of my friends who couldn't see their horse. Yeah. And some people thought, Oh, this is a nice, you know, it's an, you know, this is a nice thing to do. Everyone can have a virtual sort of pony ride, but I know that I had a few friends on group chats and what have you that that were, you know, unable to see their horses at all and they've really struggled with that in a bad way, you know, which is, which is a shame, but it's just everybody just dealt with it differently. I think it would be easier for those who at least could get half an hour but certainly the ones who got closed out for a number of weeks. In all honesty, you know, 99% are forced to leave. We have them for that reason.

And But everything going up in the air and you know, work and all that kind of stuff. I mean, I wouldn't have coped at all if my horses weren’t at home and I couldn't see them, it would have been would have been a heck of a lot worse. Yeah. However, it would have been awful, actually. So I can totally empathize with them. But yeah, I definitely think you could see people starting to crumble. Yeah. Yeah, a little bit. And luckily, the odds kind of, I think opened up about the night, you know, like, I think if some of the yards had stayed closed for longer they would have been, would have been worse. Yeah, I think it got to breaking point.

**AW: Yeah, I think that's really interesting and quite perceptive. I think you have obviously navigated that quite well because I didn't consider the difference, you know, the people who couldn't ride the horses or couldn't even see their horses. Obviously social media is such a huge platform for horse owners to put everything up. It's lovely to have pictures and videos of you and your horse doing what you love. But actually, that could be upsetting for people who couldn't see their horses, and to then have to see other people having the time of their lives. One person I spoke to had a bit of a conflict- she kept riding was riding and she was confronted by a woman who was shouting at her from the car that she shouldn't be riding and that she was being irresponsible. She was just taking our frustration on or maybe and she was just upset. But yeah, I think there's been about it seems as though there's been a bit of a debate between the two groups.**

HH5: Absolutely. Early on, it was early on, as I say, again on social media. And again, another reason why I didn't post anything about me riding. Yeah, was that there was a very strong and, and sometimes fairly aggressive divide between the riders and the non riders. And which was quite interesting and interesting to see who, you know, and actually, a lot of the, a lot of people on the livery yards would actually probably have wanted to ride, but it was a lot of the people who had their horses at home and had I suppose the option, who decided not to, but yeah, I mean, it was there was you had to just be a bit canny. About what you what you've said or posted because some people felt very very strongly about the fact they shouldn't be riding yeah and which is bad enough you know like if that's how they feel but again it's, you know struggled with people really having to go and enforcing their views or shouting at other people you know? Yeah, it wasn't never made a law that you couldn't you know, it was at your own risk and I mean, I hope that people weren't you know, doing anything too stupid. I mean, you never know what the horse when might come off it but I mean, there's ways of limiting the risk and like I didn't go galloping around the countryside for the first you know, wee while and stuff like that. So yeah, the for the month, I walked out hacked around, you know, and that was it, and did a little bit in my field here. But um, I mean, everyone was different, but I can't believe she got shouted out her window.

**AW: I think it was first week as well. She'd spent a long time managing his weight and really didn't want to lose that, especially when, yeah, sort of spent such a long time getting on top of it. So, I think she felt a bit attacked and it doesn't sound. It doesn't sound like the nicest situation.**

HH5: Well, I mean, that's it. I mean, I, it makes me wonder as well, you know, how many people have gotten decked, getting going up a bit of pace? How many people was caught back to getting back on their horse after, like, two months of not doing anything? You know what I mean? Yep. Yeah. I think I it's just a tricky one. It was just a tricky one. But I just again, and it's, you know, social media, or this woman at least did a face to face I suppose, it was a very strong divide and people felt very strongly, about it. There didn't seem to be a huge amount of middle ground for people to be left to their own devices.

**AW: And obviously the main reason was this idea of limiting the sort of pressure on the NHS. So, if something happened, you would not want to be adding to the chaos there. But then, really, with the concern over second waves and things like that, and with the knowledge know that it was going to be quite a long time that people couldn't ride for, maybe the advice should have been more structured and taken into account each individual animal rather than just blanket don't ride?**

HH5: Yeah, I think it would have been, I think, I think it would have been better if we'd have had stronger guidelines, you know, like maybe a stronger guidelines. I think, because it's all been a bit wishy washy, everybody's taken it, you know, perceived it in their own way. And, and it, you know, nobody was wrong, and nobody was right, if that makes sense. And there was no, you know, we advise you don't ride, but we're not saying you can't ride, but it said that, you know, but with but you know, they didn't say yeah, as you're saying like, but if you have a horse that has a condition that means they have to be ridden or bah, bah, bah, bah. It's just it was it was just very grey as was the, you know, the guidelines towards livery, you know, it was very much, quite rightly, in some ways left up to the livery yard owner, but it made it again, very difficult. So, you know, that's for them to decide what was right or wrong. Yeah, and it might have been easier to, but then I suppose there's so many arrangements and so many different situations. How do you know just one? You know, one way of working that works for all of them. You know, you've just got livery yards with 10 which are quite different to one that's got 60. Yeah, no, yeah. I don't really know how they could have addressed it better. And at the end of the day, you know, in any situation, people are always going to argue with each other. Yeah. It doesn't, and people are always going to break the rules, even if it is a rule, you know. So, a lot of there is a few people, you know, obviously traveling several miles to go hacking during actual lockdown and things like that. Yeah. Which, you know, cause it, it's not something I would have done, but yeah, there was a lot of there was a lot of grey area.

**AW: Who do you think would have been the best serve body to issue guidance? Do you think there's a particular body or group or organization that was in the best position to do that?**

**HH5:** I mean, I suppose the best Well, you know, I suppose the BHS is the is the best known? I would say they're probably the widest known one. Yeah. Everybody. And, and, you know, probably the wider sort of respected one. By all by all disciplines and all, you know, sort of across the whole board. Yeah. You know, would have doing things differently have made it any better? I don't really, I don't really know. I don't really know how I don't really know what would have made it any better. No. It was just a rubbish situation that no one ever expects to be in, you know.

**AW: Well, that's it. That's all the questions that I would like to ask. Do you have anything else that you think is important to include in our conversation about welfare or changes that have happened during the pandemic?**

**HH5:** Not massively, I mean, I suppose the next sort of wave of problems we're going have will be a lot of people are getting laid off now. Yeah. Which is a you know, a lot of the furloughed workers and things like that are starting to I'm seeing are starting to lose their jobs over the next month or two. And that I think, will be the next. The next problem I know, I know a couple of people who've moved yards, you know, to a cheaper yard and things like that recently because I know that about be made redundant and things then. So I think, Yeah, that'll be the next thing that pops up that that could potentially cause the sort of wave of problems within sort of the echoing community around here,

particularly around Aberdeen, you know, because of the, the oil and things like that. And, yeah, so about something I'm just sort of tentatively waiting for, to sort of kick off a little bit, but hoping that, that people will, you know, everybody's really supportive and hopefully, it'll be manageable, but I'm a little bit concerned for these people. Yeah. You know, now facing this. Yeah, potentially losing jobs. I think that's probably the next thing we are going to see.

**AW: That's a really good point- I hadn't even actually hadn't even considered that our location might be at the forefront of being put in that position because obviously as you say, the oil and gas industry has been quite impacted by it. Do you think in your view, re people on the whole likely to kind of prioritize rehoming their horses?**

HH5: Yeah. And you know what people would rather, you know, go without for two months rather than the horse go without food. So I'm really I think, you know, that welfare of the horses will be will be paramount generally. And you hope that if any of them ever got point of being really stuck, they either ask for help or decide, right? No, you know, I can't manage it anymore. I'm just gonna' have to, you know, rehome or sell it, what have you, but I think, yeah, that will probably be the next Yeah, hope. Like I stated. We generally put the horses always first, as far as we can, and so on. Hoping, you know, it won't become a welfare thing. But I think that's probably the next thing that might, that might sort of cause a few issues. Particularly I mean, you know, if it's someone who maybe isn't oil and gas or something like that, and they haven't had a huge amount of money anywhere and they've lost their job or something, you know, things like that. I think that's the one we kind of got to look out for a wee bit more.

.

**AW: Yeah. Yeah. That's really insightful. Thank you so much for all of I really appreciate your help there. That's been a great conversation, tons of really useful information. Really good. And I'll keep you if you're interested. I will keep you up to date with any sort of publications or anything that we produce from this study. And yeah, have been brilliant and we can stay in touch and I really hope that things are things sort of picking up for you.**

HH5: I've gotten dribs and drabs. I'm not sure I think I'm tentatively looking out for the normal, normal, inverted commas normal job. And really, yeah, I've been self-employed two years last year had an operation. So, I had two months off unpaid. And then this year, I had a pandemic. So I've had like, three months off unpaid, and I'm a bit like, so I don't know unless, people maybe he'll start going on holiday soon, but I'm not sure I'm gonna' have keeping an eye out for that a normal person job. Yeah, see what happens within a month. I'm getting enough to tick over at the moment anyway, so Oh, that's good.

AW: I really hope that things pick up for you. And you know, if they do, and I'm sure you'll be in a good position to start. I get the impression that you're very highly regarded in the industry anyway. So, I think you'll have plenty of assistance with people working for you.

END-----------------------

Interview 5

HL5

AW: Could you outline how you keep your horses and your day to day management of them?

HL5: Yeah, so normally through the summer they would mostly be out. One of them has chronic EMS / laminitis which has gone on for like a year a year now, she is currently in pretty much full time, because she had quite a bad flare up like 6 weeks ago.

AW: Do you use livery services to look after them?

HL5: Yeah, so they are on grass livery at the yard I work at, so that makes it a little bit easier. I am furloughed just now so I am stuck to a morning time and a night-time. But normally I would be there all day with them.

AW: Have you found that your management of them has been impacted by the pandemic?

HL5: Yes, in terms of us all having time slots to be at the yard, so I am stuck to fairly early in the morning and late at night.

AW: Is that allowing you to do everything you want to do with them?

HL5: Ehm, it is, it is a little bit, like the one that is in I would much rather give her smaller amounts of hay during the day, rather than giving her one larger hay net in the morning and leaving her to night.

AW: Have the yard been quite flexible in allowing you to look after your horses, especially managing EMS?

HL5: Yes, [the yard] have been pretty good, especially in terms of having a lot of vet visits and stuff so I have needed to be up at random times for vets and things, so they have been quite helping.

AW: How do you find vet visits with social distancing?

HL5: Well it was actually pretty easy; they know my horse quite well and they know she is not really a people person. So even outside of lock down they are always quite quiet and distanced. So, it was quite normal.

AW: Do you feel like you have had enough information dropped down to know what you're doing with regards to social distancing and hygiene?

HL5: Yes, but I don't think all of the liveries have had access to all of the information that I have had. Because I work there, I have the chance to talk to the owners more and ask more. No one has been stuck or confused, but they probably could have had more information.

AW: Does that mean that everyone has been doing different things or working to different rules at times?

HL5: Well, everyone is kind of, some people took all their tack and feed home and removed everything they could from the yard, whereas others have just been more careful like making sure we don't have two people in the yack room at once. So, it has kind of varied in terms of personal preference, I think.

AW: Have you been exercising your horses during lockdown?

HL5: Yes. The EMS mare – this is her first time back in proper work in a year, so it is nice to have her going again. It is a little bit hard trying to fit in riding two in one evening.

AW: Do you prioritise your riding?

HL5: Yes.

AW: I suppose with you working on the yard you may have predicted the need for the horses to get exercise. In some cases, horse that have been causing problems- is that something you considered?

HL5: Hem, not so much with us. We evaluated how sensible all of the horses were. A couple of the school horses have been kept going quietly, but anything that is a bit sharp has been turned away. Also, we don't have an awful lot of grazing on site, so we have ended up renting fields in different places, so it has been quite well managed in that the fat horses have a different field from the lighter ones.

AW: Has keeping those fields been difficult?

HL5: Yes, it has been difficult in terms of people feeding horses. Our fields are easily accessible. So, the EMS mare could probably be turned out for a short time, but I am wary of people bringing her apples and carrots. We are being very careful with her; soaking hay nets and she is in all of the time. I am scared to let her out, even for a couple of hours with people. I would maybe put her out or for a couple of hours early morning, but not while people are there feeding them.

AW: The feeding sounds like a big problem just now?

HL5: Yeah it is, we have got signs and we have tried to explain politely to people not to. Some of them listen and some of them don't. We had one of our liveries who was away at the vets with quite a bad colic. Obviously, there is no proof of what could have caused that. But one of the big things was, what have people been giving her?

AW: With riding, do you usually ride along public footpaths?

HL5: Yeah, a lot of them. Initially they were quieter, but then there was a sudden surge of people all the time.

AW: Has that been restricting access for people to ride there?

HL5: Yeah. Some people probably have been put off from riding. Because I tend to ride quite late, it is probably quitter by the time I am there. But a lot of people have been either not hacking or have been looking for different hacking.

AW: At the beginning of the lockdown, did you have any plans that you were going to implement to minimise the impact of the restrictions?

HL5: Yeah, so potentially we didn't know if we would have any access to the yard, so I looked into a field for if I needed to turn one of them away for a few months, that she could have gone out safely there. So, we did have a bit of a back-up, but luckily it didn't come to that. We wrote up care plans as well, so we have a folder for each livery horse with details so that if someone had to self-isolate for a couple of weeks.

AW: Were the liveries receptive to the rules on the whole?

HL5: Yeah everyone was really good, we just have a small group and a lot of them are doctors and former nurses. So, everyone took it really seriously. The yard owner, and one of the liveries are both asthmatic, so we had to be more careful about them anyway, so everyone has been really good.

AW: Were things set up to handle farrier visits quite easily?

HL5: Yeah, our farrier is very good. So, he is quite happy to just do horses tied up. Most of the horses are pretty good. We have quite a lot of outdoor tie up space so you can tie up the horse and stand back. It's been really easy. So often for the school horses the farrier would have gone and taken them out himself, but instead we have just brought them to him, so he hasn't had to touch anything other than the feet really.

AW: Have you noticed an increase in obesity in the horses that you are around?

HL5: Yeah, it is worrying. We had two come in with fairly serious laminitis in the past two weeks. One which we think is an EMS case, but the other has never had it in his life but is pretty much off his feet. Yeah, and I think the biggest thing is that it hasn't even been the ponies that we have expected. You always have some that you watch a bit more closely. So, they all seem to be fine, and it is the ones that we thought would cope with more grass, and that they would be fine, that are not fine.

AW: In your opinion, what is it that you think triggered that?

HL&: Just that most have had to be turned away. Our school horses get turned out in groups in the indoor school overnight, so they tend to live on hay – but with 16 hours turnout a day. Because we

have had to furlough a lot of people and just keep one person working, a lot of them have had to be turned away onto grass- probably for the first time in a couple of years for some of them.

AW: So, the main reason has been the staff members being lost, and things being too busy?

HL5: Yeah. We normally rotate them and give them all a break each year, but your more laminitis prone ones get a break over winter and we would watch who is turned out in the spring. But now they have all been turned out onto rich grass.

AW: Were you were concerned when you knew they were going to be turned out?

HL5: Yeah, but again, the ponies that we were worried about we turned out onto different fields. So, it is the ones that we weren't worried about that are now having problems.

AW: What would you say have been the main challenges to you during the pandemic?

HL5: I think just trying to, because we have such a big number of ponies, trying to come up with the most appropriate plan for all of them. Obviously, that hasn't worked in all of the cases, but a lot of time and effort went in to trying to secure the right grazing and the right herds.

AW: Would you say the impact has been greater on bigger yards?

HL5: Yeah, or maybe because we are mostly riding school with only 8 liveries. I don't know if it is different for us but being a big yard has meant it has been a lot more complicated.

AW: Is there anything else that you think is important to include in the conversation?

HL5: I know at the start we were concerned about whether horses could spread it. So, if someone touched a pony in a field, could the owner then get ill from it. I think I haven't been aware of there being evidence that it would be a thing, but that was a big concern at the start. We were kind of wondering how safe it was for people to be allowed near them.

AW: So, do you think it would have been good to have more information on biosecurity?

HL5: Yeah, there is so much for hard surface, and disinfecting wheelbarrows, but there was no consideration of – if you have to move someone else's pony back at the gate; do you have to disinfect the pony?! There was official guidance that cam out to say that people don't need to keep their cats indoors, but I haven't heard of anything official about horses.

AW: So, the information that came out then didn't really cover the concerns of people who had their horses at grass.

HL5: Yeah, it was all just about it being on hard surfaces; gates and wheelbarrows and stuff, but nothing really about horses.

AW: Were there any equine specific bodies that you were looking to for advice?

HL5: A lot of ours has been Horse Scotland. The BHS advice we would read, but it was quite often conflicting with the horse Scotland advice. So, we would go with horse Scotland over that.

AW: Were horse Scotland quite good?

HL5: I think as a riding school we have to use them, so that is maybe why we are more aware of what they are saying. In terms of what is allowed for coaching, it has been changed every 10 minutes. But they have been our most reliable source.

AW: Would a more collaborative approach would have been easier to follow?

HL5: Maybe more collaboration would have been useful. Definitely. We have been reading it all from Facebook, but it has just been a case of the bhs saying one thing, and then the next day horse Scotland say, actually no you can't do that. Which probably is most to do with coaching, but it is hard to know who you can trust when some people are saying you can ride indoors, and other groups are saying no you can't.  But yeah, Facebook is mostly where we have been looking.

AW: Did you feel it was difficult to trust the governing bodies before the pandemic, or have you lost trust during the coronavirus?

HL5: Yes, I think we have lost trust in the BHS to be honest, we have definitely kind of, like, a lot of what they have said, they aren't really in a position to say. With regards to coaching and stuff, and a lot of their advice was conflicting. It went overnight from we are advising everyone that its not appropriate to ride, and then they said they were actively encouraging people to ride again- in the middle of the pandemic?! I've been riding the whole way through, but I think actually encouraging people when you are not the official governing body for the sport in the country is not idea.

I know we were really struggling to find guidance on how many people could be on the yard at the one time, because people were telling you to be careful of how many people you could have on the yard- but there was no guidance that said- if you have this much space, you can have this many people. We just went with what we felt was safe, but there was nothing official on that.

Interview 5

V5

**AW: Could you please explain the main ways that your work has been affected by the pandemic?**

**V5**: Yeah, so obviously we are down to a skeleton staff, large animal, we are a mixed practice, so we do large, equine and small animal, so we were essentially trying to work from home where possible. It was quite difficult. So, reception would call, and you would possibly call back – horse owners in particular- obviously because farm work was still going ahead as normal. So, we were trying to do the whole, tele-medicine thing, and triage across the phone, and see if it was a genuine emergency or something that couldn't be put off for three months. So those were the two criteria; genuine emergency and something that couldn't be put off for a long period of time. That was basically how we were working, out of hours was working just as normal, because that is what you would do anyway. But with the working from home there were less people in the building, less people available to do calls, and it just made it that much busier, I guess.

**AW: How have you found consulting over the phone?**

**V5**: Yeah, so to be fair, I think that was more relevant for small animal, because you don't get the same sort of phone calls about horses, generally people aren't- its quite expensive, so people aren't calling you out willy nilly for the horses, So weren't having too big an issue with that. It was much easier, it was more, what's wrong, is it bleeding, is it lame, how lame, how long has it been. Whereas it was more difficult for small animals because it was a lot more details, and they are a lot more used to coming in for minor things, than the horse community were. So, I don't think that part was too bad because it was exactly what we would do for out of hours, just all the time. So that part was fine. So, we were probably quite used to it. And to be fair, our clients weren't' calling us for stupid things, we had a few, can you do a blood tests for strangles because I want to move yards in lockdown, and in that case, we were like, no. Its just a premovement test we are not doing those.

**AW: So, were the calls you were receiving sometimes worded in a way to catch attention?**

**V5**: Yes, well we didn't have that a lot, but I personally did have one horse person. It is a well-known client and when we went down the route of, no it is not an emergency dental, we then changed up the story to – she is having difficulty eating, she's not right, you know. Not many people have done that but there have been a select few. And I guess it is like if you are phoning the doctor, there are buzz words that mean you have got to be seen. I think people at the start didn't want us to come out, as there was definitely a bigger fear of coronavirus at the start and we were much quieter during the first three weeks of lockdown. But gradually each time lockdown was loosened, people have become braver or more sick of it, and have gradually increased phone calls for non-emergency things that they weren't bothering about before.

**AW: Okay, and with regards to laminitis, have you seen a change in the way that is managed during the pandemic?**

**V5**: To be honest, which I find quite bizarre, I would say we have seen less cases this year than we did last year. Because last year was insane, it was like a bonanza for laminitis and EMS. I don't know

whether it is because people aren't phoning us, or, I can't understand why there would be less, because people have been riding less. But I definitely think we have had less cases of laminitis this year.

AW: That is very interesting. Is it a concern that people might be holding off from addressing things that actually do merit veterinary attention?

V5: Possibly. I feel a little bit like that with laminitis anyway, because it is always obese little ponies and that is not something people are often willing to take on board. You know, EMS and things like that are just a case of dietary management as well as initial pain relief. But I think I have definitely seen obese horses this year I would have said. Because there are a few that have been turned out and not done much. I would say there are a few that I have been out to and said, "Oh my god that didn't look like this last year!", but yeah, I don't know if it is that people aren't phoning. Maybe money is tighter because a lot of people will be out of work and not getting paid as well.

AW: That is interesting how varied the observations of the number of laminitis cases have been.

V5: Yeah, well I mean last summer I would say without a word of a lie we were probably going out to a severe case of laminitis every three days at the least. Last year was just mental we couldn't get enough Styrofoam blocks. But no, I can think of only a handful of cases this year, which is good for the horses. And generally it is difficult to know for sure because we have been on a skeleton staff and we haven't seen each other, But I tend to keep a tab on the horse stuff going on so even if I haven't been out to it myself, I would have read about it or have discussed it with somebody- so I can just think of a few severe cases we had, but nothing like last year.

AW: And with the severe cases that you had this year, were you able to manage follow up visits as usual?

V5: No so we basically, probably did slightly less follow up visits, but we probably… the most recent one was a huge 700kg cob x, and we were out maybe 2-3 times to start with. And once we had it stable enough that we were happy we just got the owner to update us. Perhaps we might have gone out more usually, but I don't think a lot more. It warranted us going out and the guy was happy to give us space so yeah.

AW: Have you found yards on the most part good at providing you with what you need to work safely?

V5: Yeah, it is probably just up and down. One place you go to, and I don't think its anyone trying to defy the rules, but I think some people just forget, and then other places are quite OTT- which is good, I guess. But yeah, some places people are just milling about as usual. And you just try to skip around people when they are trying to stand a chat. But to be fair most people have had horses tied up and things like that. We have, where possible, and it has been difficult, but we are flexible enough that we have a few people on standby that we could call in if things were busy. And what we have been doing is that if it is anything beyond, sort of, routine vaccinations, generally we have been trying

to take somebody else with us so that we don't need to have any reliance on the owner being with us, or at least they can keep the 2 meter distance from us.

AW: It sounds like a really tricky problem the social distancing because you only have so many hands.

V5: Yeah, well with large animal it has just not been possible, with calving's or caesareans keeping the distance is just not possible.

AW: Do you think the guidelines addressed these sorts of things that you were going to need to know?

V5: I think probably we have been making our own decisions based on common sense, and we have definitely been happy to increase what we are doing so long as we can increase social distancing or use masks. And as a practice I don't think we see that there is any issue with carrying on almost as normal. Because now you are getting all of the things coming out of the woodwork that was postponed earlier, so now that we are a few months in it is busy. The small animal as well is absolutely mental with all of the vaccines.

AW: Did horse owners seem like they had enough information to make informed decisions?

V5: Yeah, I do think so. I think that maybe shows in the way that we weren't getting the same phone calls, they would still phone for advice now and again if there was something that they weren't 100% sure of. But I think based on the fact that we had very few horse calls, although lots of people were obviously irate about the vaccine situation to start with, but then. I think the fact that people weren't phoning as they would have and we were definitely less busy and we weren't inundated with people phoning asking can you come and do this is it okay if you do that, or whatever, So I think that hopefully means that people were understanding what was going on and were following their guidelines.

AW: So, all in all do you feel like things were handled well?

V5: Yeah, I think so, yeah. We were certainly writing happy as a practice with what we were doing. I think the horse guidance was okay, I think the small animal stuff was diabolical, but again I think it is probably easier because you're not having questions like, do we still need routine flea and wormer. You know, people are more used to treating horses for minor ailments anyway.

AW: Is there anything that you have noticed that you worry about in terms of the impact of the pandemic on equine welfare on the whole?

V5: I think probably the lack of exercise for some horses. It did kind of worry me – although I agreed with it – was that places were stopping people from riding or exercising their horses. I agreed with it in terms of the pandemic, and to protect the NHS, but I worried about it in terms of the obese ponies that were probably just out on grass now. And I think in terms of lameness and ailments people were phoning appropriately- I don't think anyone was leaving ponies unnecessarily suffering or anything –

apart from the obesity. But then people don't really associate that with a welfare issue unfortunately. Whereas we obviously would, but a lot of owners don't. And that is across the board.

AW: That is very true and a tricky one to get to the bottom of. Do you think some sort of traffic light guidance based on an animal's characteristics should have been implemented to encourage people to consider exercise on a more individual level?

V5: Yeah, I don't know. It's hard to say because I have always had horses and even the best horse in the world, you can have an accident with. All it takes is something to fly out of the grass and you are underneath it. So yeah maybe with youngsters and more dangerous horses then that might have been a good idea. But, I think, you know, you've got horses, and anything could happen at anytime so that is tricky. But maybe more in hand or lunging or something like that. That would be fine. IF yards were promoting no riding at all, I wouldn't have a problem with that, but maybe ensuring more lunging or long reigning or something.

AW: Do you think there is scope for guidance on preparing a pasture for maintaining a horse during the pandemic for horse owners that might have helped?

V5: Yeah, of course, I think that would be good. But you know, you can give people as much information in the world and they will just do what they want anyway. I was thinking of one yard in particular, I spent a year getting this pony, you know she had horrendous EMS and chronic laminitic case, she could barely move and had 12 – 13 degrees of rotation in her front feet. So, I spent a year getting her back on her feet and I saw her not that long ago and she was just turned out in this lush grass paddock. And I just thought, "Oh no, what are you doing". And they knew because we had her out in the school- a no grass paddock for so long – and …. I think that would be a good idea, but again, people will use it, or they won't. I think it is probably difficult for people who have just a little bit of land, or their have horse on a yard and they don't have control over where they go. And they would have to pay more to have them separated in a starvation paddock or whatever. So, I do appreciate that there are lots of different factors rather than just, this is what I want to do. Some yards are obviously a lot more flexible than others, and easier to ask to implement changes.

AW: Is there anything else that you think is important to include in the conversation about the pandemic and its impact on horse welfare in general?

V5: I don't think so. I mean, I don't think horses will have, overall, done badly out of it. Having maybe a year off won't have been a bad thing for most, but I just think the management while they are off is just a wee bit of a concern.

Interviewer: Ashley Ward (AW)

Interviewee: HH6

Date and time: July 10th , 2020, 10:00.

Location: Aberdeenshire. Remote

Recording was poor quality on this interview: Notes were taken throughout- direct quotes will be used to supplement the transcript.

AW: Could you describe how you manage your horses on a day to day basis?

HH6: We are on a working farm, with four ponies.

Actually, quickly after I stopped riding, my highland developed a very severe bout of laminitis.

AW: Do you feel that the reduction in exercise contributed to the laminitic episode?

HH6: Well, I think it was a combination of things, yes, the reduction of exercise, as well as a bit of taking my eye off the ball. At the beginning of spring we had lots of warm days, and frosty mornings. I was more concerned about the other ponies than mine developing laminitis. The farrier was due just at the beginning of lockdown, and so their trims were put off. The Connemara has shoes so it was shod, but we decided that the others could wait a couple of weeks- in line with not doing any unnecessary shoeing. So they weren't trimmed. And so their feet were long for them, longer than they would usually be.

AW: Did you have the vet to attend to the laminitis?

HH6: Yes, we couldn't actually move the horse, so we needed the vet to come to give him pain killers and the rest. The vet was happy to follow the farriers advice and we all had quite an extensive conversation, before we decided that the horse would be trimmed 4 -weekly, and I kept him on a very deep bedded paddock without grass. Actually we added shoes for more support a little after that, but we had a flare up again. He is only just now getting back to normal work and seems to be staying sound. After this I just got the others back in work again, because I thought- I can't have that again and restrictions were lifting by that point.

AW: How did you make the decision to stop riding? Was that based upon government guidance or upon equine specific guidance?

HH6: That was from equine specific guidance. I am a nurse, so I was very aware of the pressure on the NHS and wanted to minimise that as much as I could- so I decided it wasn't worth it to ride. Would I have kept them in work in hindsight? Maybe, I'm not sure. You know, I think it was important to not put yourself at risk, and we didn't know what was going to happen. So if some horses end up with problems because they weren't ridden- realistically that might just be something we have to deal with. It's a full blown pandemic- and they are animals. My opinion is that they will survive – as long as they are safe and healthy, they can come lower on the scale of priorities.

AW: Do you think that the guidance given covered the risks of not riding, or of changing the horses routines?

HH6: More specific guidance would have been better. I think their could have been more guidance from equine specific bodies, and more details would have helped with making decisions.

AW: Which of the equine organisations do you think would be in the best position to issue this guidance?

HH6: I think the BHS, I mean, most people are members. That is ranging from people with animals as pets all the way up to those affiliated in competition. They are the most visible. When I looked up information on the web I then saw the British Equestrian Federation I think. They were giving advice but I hadn't really heard of them before.

AW: Do you have any concerns for overall equine welfare in the NE region?

HH: I would say the lack of access that some people were facing during the lockdown. With livery yards closing their doors, I don't think that was a fair thing to do. As well, probably a lack of regular farrier care. With farriers maybe visiting less often-I think a lack of trimming might be cause for concern.

AW: Is there anything else you think we could include in the conversation that you think is important?

HH6: I think just that clearer guidance would have been useful. The vets were quite quick to get back to normal so that was good. But one body, giving clear advice would have been helpful. I have been really lucky to have horses at home. But I also think that starting horses back after a period of time off might be quite dangerous. These animals in some cases haven't seen others for a long time, you know once they all get back together there might be some concern over rideability.
